# Trauma of the midface

**DOI:** 10.3205/cto000121

**Published:** 2015-12-22

**Authors:** Thomas S. Kühnel, Torsten E. Reichert

**Affiliations:** 1 Department of Otolaryngology, Head & Neck Surgery, University of Regensburg, Germany; 2 Department of Maxillofacial Surgery, University of Regensburg, Germany

**Keywords:** rhinology, trauma, midface fractures, orbit, ESS

## Abstract

Fractures of the midface pose a serious medical problem as for their complexity, frequency and their socio-economic impact. Interdisciplinary approaches and up-to-date diagnostic and surgical techniques provide favorable results in the majority of cases though. Traffic accidents are the leading cause and male adults in their thirties are affected most often. Treatment algorithms for nasal bone fractures, maxillary and zygomatic fractures are widely agreed upon whereas trauma to the frontal sinus and the orbital apex are matter of current debate. Advances in endoscopic surgery and limitations of evidence based gain of knowledge are matters that are focused on in the corresponding chapter. As for the fractures of the frontal sinus a strong tendency towards minimized approaches can be seen. Obliteration and cranialization seem to decrease in numbers. Some critical remarks in terms of high dose methylprednisolone therapy for traumatic optic nerve injury seem to be appropriate. Intraoperative cone beam radiographs and preshaped titanium mesh implants for orbital reconstruction are new techniques and essential aspects in midface traumatology. Fractures of the anterior skull base with cerebrospinal fluid leaks show very promising results in endonasal endoscopic repair.

## 1 Introduction

The present review article on lesions of the midface places the focus on some important aspects, however, the description in other contexts remains limited. We tried to take the incidence of scientific assessment into account as well as the partly controversial discussions in literature indicating the current interest of our disciplines in certain topics. The main focus is placed on bony injuries. Lesions of the soft tissue are mentioned only if they are important for the respective pattern of injury. Of course, it is very important to treat them appropriately because even in cases of perfectly reconstructed bony skeleton, scars may lead to deformities and dysfunctions that can only be corrected secondarily with significant difficulties [1], [2].

Trauma of the midface regularly lead to lesions of soft tissue, teeth, and bony structures of the skull including the maxilla, the zygomatic bone, the naso-orbital and naso-ethmoid (NOE) complex as well as supraorbital structures. Not rarely, those lesions of the midface are combined with injuries of other parts of the body [3]. Patients with midfacial fractures who do not undergo successful or appropriate treatment may suffer from significant long-term consequences such as disfiguring scars, bony deformities, or even loss of vision [4]. Relevant emotional and psychological problems may result from trauma [5], [6]. The successful treatment and rehabilitation of patients with lesions of the midface requires a profound knowledge of the anatomy, fractures, and techniques of osteosynthesis. Additionally, special knowledge in the field of occlusion, physiology of the eye, and skull base surgery are essential.

## 2 Basics of traumatology of the midface

The etiology of midfacial lesions has changed during the last three decades and still continues. Patterns of trauma differ regionally [7], [8]. Disorderly conduct as origin of midfacial trauma are especially dependent from the region. In the last 10 years, on the one hand increasing trauma of the midface was observed because of domestic accidents as consequence of an ageing society in the western industrial countries, on the other hand, sports accidents are found more often in younger people [9], [10].

Injuries of the lateral midface (63%) occur more frequently than central ones, males are clearly affected more often than females. There is a peak age in the 2^nd^ and 3^rd^ decade of life. Street accidents occur more often than sports accidents [8]. In 43% of patients, cranial nerve disorders are revealed. Sensitivity disorders or the infraorbital nerve are most often, followed by lesions of the facial nerve [11].

For midfacial fractures, the fracture should be treated within the first two weeks. Afterwards the beginning bone absorption at the fragment surfaces and the beginning callus formation leads to difficult reposition to the anatomical correct position. After an interval of 2 weeks, the treatment is considered as delayed and is based on the principle of secondary posttraumatic treatment. Primary care for fractures should be performed as soon as the general condition of the patient allows therapy. The limiting factor for immediate treatment of the fracture is mostly not the fracture itself but the patient’s general condition.

Independent from the severity and the fracture type, the basis of successful therapy of midfacial fractures is the restoration of the supporting pillar of the midface, the bony prominences, the bone cavities (e.g. orbit), and correct occlusion [12], [13], [14]. Definitive surgical therapy aims at an exact three-dimensional reconstruction of the skeletal structures in order to restore the face with its original width, height, and sagittal projection [13].

Since the introduction of plate osteosynthesis via approaches with optimal overview, the treatment results of craniofacial fractures have been improved significantly. For therapy of pediatric trauma, adequate procedures have to be applied. This means in particular that the extensive detaching of periost from the bone should be avoided as far as possible because otherwise growth disturbances might occur. On the other hand, it is a principle for children as well as for adults that the reposition in cases of relevantly dislocated fractures has the priority. Greenstick fractures do not require necessarily osteosynthetic stabilization after reposition. Absorbable osteosynthetic material is an alternative to titanium [15]. 

### 2.1 Anatomy

**Figure 1:**
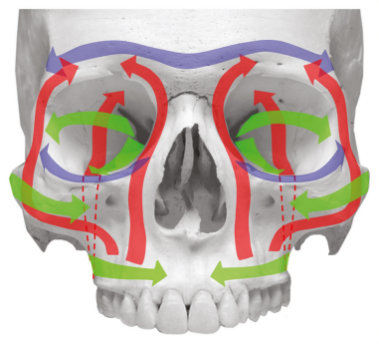
Course of the vertical and horizontal trajectories of the midface

The midface consists of the following bony structures: nasal bones, lacrimal bone, ethmoid, sphenoid, maxilla, zygomatic bone, and palatine bone [16]. The mentioned bones merge to the facial skull in a particular kind of lightweight construction with typical framework construction and reinforced trajectories (Figure 1 (Fig. 1)). The vertical trajectories, i.e. the supporting pillars of the midface, are responsible for transmitting the masticatory forces to the skull base and the bony structures of the neurocranium [17]. The first major force line draws from the alveoli of the frontal teeth and canines via the delineation of the piriform aperture to the frontal process of the maxilla and to the frontal bone. The second major force line draws from the area of the premolars and the first molars via the zygomatico-alveolar crest to the zygomatic bone and from there to the frontal bone via the lateral edge of the orbit. The third major force line draws from the distal maxillary molars via the maxillary tuberosity and the pterygoid massif to the skull base [16], [17]. The transversal trajectories of the midface are formed by the supraorbital and infraorbital bone margins and the alveolar process of the maxilla [18].

The sites where vessels and nerves emerge are of major importantance in traumatology, as they pose structural weak points that influence fracture lines. In the area of the anterior skull base the following foramina are concerned:

- The foramina of the cribriform plate where the olfactory fibres emerge.
- The pathway of the anterior and posterior ethmoid arteries.
- The optic canal.
- The superior orbital fissure with the nerve responsible for oculomotor functions, that however plays only a direct role in extreme injuries.
- The infra- und supraorbital foramen with its respective portions of the trigeminal nerve. Fractures affecting those foramina indicate surgery based on clinically apparent hyp- or anesthesia.

### 2.2 Classification

Today, fractures are mainly classified based on their radiological presentation. Because of the immediate functional clinical significance, the classification of midfacial fractures according to Le Fort is still applied and will be described here. Classifications according to Esher, Wassmund, Gonty [19] and Ernst take predilection sites such as the cribriform plate, the sphenoid sinus, the frontal sinus, and the ethmoid roof into account [20]. However, their importance is continuously decreasing.

In a broader sense, also fractures of neighboring regions will be described. Those are frontobasal fractures, fractures of the nose and the naso-ethmoid complex, fractures of the orbit and the zygomatic bone and the zygomatic arch, and midfacial fractures such as the various Le Fort fractures. 

Impact to the midface results in typical fracture types due to the particular anatomical structure and construction of the facial skull [17]. The classifications based on these observations allow scientific investigations as well as statistical assessment and comparison of patient populations. 

**Figure 2:**
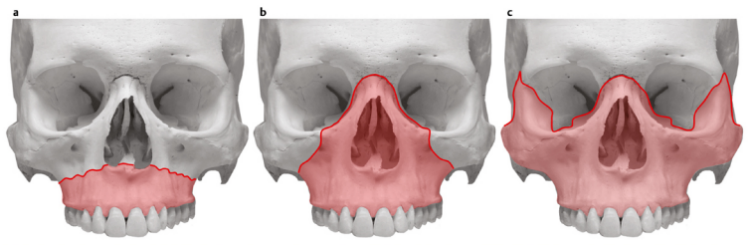
Le Fort fractures

The best-known classification of midfacial fractures is the classification according to Le Fort [21] (Figure 2 (Fig. 2)). In the context of his studies, René Le Fort (a French surgeon, 1869–1951) identified typical fracture lines in the area of the midface and the maxilla (Le Fort 1901). These observations led to a widely acknowledged classification of midfacial fractures. Fractures may occur uni- or bilaterally or in different combinations.

#### 2.2.1 Le Fort I fracture

The maxilla is separated from the facial skull in a horizontal plane above the teeth apices and the hard palate. The fracture line extends from the piriform aperture through the zygomatico-alveolar crest to the maxillary tuberosity and into the pterygopalatine fossa. From there, the fracture line returns via the dorsal wall of the maxillary sinus in the medial basal wall of the maxillary sinus to the aperture. The nasal septum is caudally fractured and often the inferior part of the pterygoid process is separated.

#### 2.2.2 Le Fort II fracture

This type is characterized by a dissociation of the maxilla, the nasal bones, and the nasal septum from the cranial skull and from the lateral midface. The fracture line extends from the nasofrontal suture via the fronto-maxillary suture through the lacrimal bone to the floor of the orbit. From there, it extends through the infraorbital margin via the facial wall of the maxillary sinus to the zygomatico-alveolar crest. The further course of the fracture line extends around the maxillary tuberosity into the pterygoid process, from there through the perpendicular plate of the palatine bone and the medial wall of the maxillary sinus via the ethmoid to the medial orbital wall into the nasion. From the nasion, the fracture goes through the nasal septum in caudal direction and ends at the posterior edge of the vomer. The integrity of the orbit is destroyed in the context of such a pyramidal fracture [16].

#### 2.2.3 Le Fort III fracture

The facial skeleton is separated from the cranial skull. The fracture line extends from the nasofrontal suture via the medial wall and the floor of the orbit to the inferior orbital fissure. From there, is fracture line extends through the lateral orbital wall to the zygomatico-frontal suture and through the zygomatic archs. From the nasofrontal suture, the fracture line draws inside through the ethmoid and the lamina perpendicularis of the palatine bone to the pterygopalatine fossa. The pterygoid process is also fractured and the vomer can be severed at the transition of the sphenoid bone.

In the context of Le Fort II and III fractures, the cribriform plate might be injured because of fractures in the area of the ethmoid bone leading to a possible CSF leak. The frontal and sphenoid bones may also be affected.

### 2.3 Approaches

**Figure 3:**
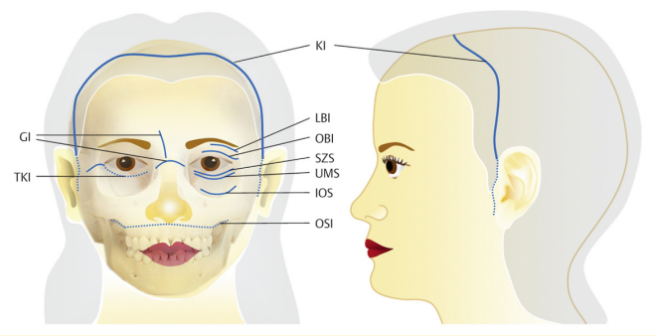
Surgical approaches to the midface. The definitions of the incisions are listed in Table 1. Modified according to [13].

The individual approach depends on the underlying fracture: a transoral approach, transconjunctival incisions, an intranasal approach, or a transcutaneous approaches may be appropriate [13], [18] (Figure 3 (Fig. 3)). 

The choice of the operative approach to the craniofacial region and the anterior skull base depends on the location and the extent of the midfacial lesions. The access is chosen for optimal overview to facilitate reposition and osteosynthesis. At the same time, the incisions should be performed in an esthetically discreet way (e.g. parallel to skin lines and in consideration of esthetic units) and include the option of expansion [13]. It is crucial to consider special motor and sensitive conditions of innervation in the face. Especially in recent times, it is one objective to avoid extensive transfacial or coronal approaches and to use less invasive and probably invisible surgical accesses. Those approaches are mainly the transconjunctival and intraoral incisions that are sometimes technically challenging and need more time than the traditional transcutaneous and transfacial incisions [13], [22] (Table 1 (Tab. 1)).

The broad acceptance of rigid endoscopes for visualization of complex anatomical relations, especially in sinus surgery of inflammatory diseases, led to basic changes of the approaches and the surgical techniques also in traumatology. The objective is always to achieve at least equivalent results of reconstruction with simultaneously lower morbidity. Hence, today the endonasal endoscopically assisted approach is widely accepted for fractures of the ethmoid and anterior skull base. Especially in the context of fractures of the sphenoid sinus, it is nearly exclusively applied [23].

Simultaneous, interdisciplinary interventions of a team of neurosurgeons, rhinosurgeons, and maxillofacial surgeons in cases of extended injuries mostly require an open approach. The coronal incision is favored because of its excellent overview and the possibility of gaining vascularized flaps (galea periost) for reconstruction of the skull base. A disadvantage is the relatively large surgical trauma, a long scar that may be eye-catching in bald patients, the risk of sensitivity disturbances at the forehead, and in rare cases alopecia along the incision.

In recent times, transorbital approaches become more and more important which is described in the paper by H. Gassner in this issue.

In exceptional cases, the subcranial approach is justified. It provides a good overview of the anterior skull base and helps avoiding trephination if a fracture can be treated in an extracranial, extradural way. It has its main importance, however, in tumor surgery. If this approach is not pursued via a coronal incision but via a combined bilateral incision developed by Killian via the nose bridge, an unpleasant scar results (incision according to Siebenmann).

The approach according to Killian is used for naso-ethmoid fractures if the coronal incision is not possible and transconjunctival approaches do not lead to sufficient overview. It is not needed to approach the ethmoid and the sphenoid sinus.

### 2.4 Symptoms

Clinical evidence of fractures are sensitivity loss of the skin, step deformity, diplopia, emphysema, epistaxis, and hyposphagma. In two third of cases with symptom of a black eye, a fracture is found in the CT scan [24].

In a prospective study of 2,262 patients, Yadav et al. investigated the predictive value of 6 clinical symptoms in order to evaluate the indication for CT diagnostics. Among the symptoms that were all weighted equally the following were found: flexible rim of the orbit, periorbital emphysema, subconjunctival bleeding, pain when moving the eye, motility impairment, and epistaxis. Only in 15.9% of patients with blunt trauma of the orbit, a treatment-worthy fracture was found. Patients who had none of the 6 symptoms, however had radiologically confirmed fractures in 6.3% [25].

Epistaxis is a regular symptom of facial injuries caused by forces from the frontal direction. Diffuse bleedings from the nasal mucosa or bleedings from circumscript lacerations of the mucosa can typically be treated by conservative measures such as packing or bipolar electro-coagulation under local anesthesia. Lacerations of the piriform aperture and the caudal septal mucosa occur frequently and may lead to significant bleeding from the nose. The afferent vessels can be attributed to the area of distribution of the facial artery on the one hand and on the other hand to the ethmoid artery. Threatening bleedings from the dorsal parts of the nose originate from the external carotid artery and concern the terminal branches of the sphenopalatine artery. Bleeding events in the context of midfacial injuries may not only occur immediately but also after significant time intervals. Late vascular complications are described by Newman et al. in 1–11% after blunt facial trauma [26]. If severe epistaxis occurs even several years after injury of the cranial skull, it may be a first sign of a covered aneurysm [20]. In the context of Le Fort II and III fractures, there is a high risk of blunt carotid artery lesion. Regarding those (and other high risk) injuries, Biffl et al. revealed in 41% of 249 patients a vascular involvement and recommended specific diagnostics [27]. Those diagnostic measures consist of contrast enhanced CT scans, MR angiography, or catheter-angiographic procedures [28], [29], [30]. Fractures that extend through the canal of the carotid artery may lead to pseudoaneurysm, dissection of the vascular wall, thrombosis with consecutive blindness or stroke, and carotid cavernous sinus fistulas [31], [32]. Malposition of the eye and pulsating exophthalmos are clinical signs of carotid cavernous sinus fistulas [33], [34], [35]. In rare cases, pressure-induced paresis of the abducens nerve may occur [36]. The therapy consists of radiological intervention and depends in detail on the size of the hole that connects the internal carotid artery in is cavernous segment with the cavernous sinus. Small holes are closed with coils, larger ones with endoluminal balloons. In a series of 32 patients, Malan and co-authors could preserve the ipsilateral internal carotid artery in 66% [31].

Intraorbital vascular lesions may be the reason for an orbital compartment syndrome (see also chapter on traumatic neuropathy of the cranial nerves). They appear iatrogenous after ophthalmological, rhinological surgeries and after traumas. Beside the relief surgeries of the orbit, pressure reducing measures are performed and the drainage via catheters is recommended [37]. The concerned blood vessels are mostly part of the choroid blood supply that is fed by the ophthalmic artery. Rupture of the ophthalmic artery is very rare and can only be found in most severe midfacial injuries. It is always associated with blindness.

Bleedings from the internal carotid artery may be fatal. If a severely traumatized patient receives help in time, packing is indicated. The effort may be undertaken to reduce the blood flow by temporary pressure from exterior at the neck. Catheter angiography with endoluminal occlusion will follow.

### 2.5 Radiological diagnostics

Conventional radiological procedures do no longer play a relevant role in the imaging diagnostics of factures of the facial skull [38]. 

More than one fracture was found 33.9% of 1,144 patients with midfacial fractures by Büttner et al. The courses of the fractures were mainly located in the lateral midface. Mandibular fractures are usually associated with other bony lesions. Isolated fractures of the mandible, however, were only found in 1% of cases in the context of this investigation.

The indication to perform CT scan in low-grade cranio-cerebral injuries (definition of low-grade cranio-cerebral injuries: unconsciousness, amnesia or disorientation, and a score of 13–15 on the Glasgow Coma Scale) is discussed controversially and the management is heterogeneous. There is a clear tendency to extend CT diagnosis although only rarely treatment-worthy neurological deficits are observed in this patient group. The “Canadian CT Head Rule for patients with minor head injury” proposes reasonable indications. High risk patients are characterized by the following symptoms and should receive CT scan: less than a score of 15 on the Glasgow Coma Scale 2 hours after the event, suspected open or impressed fracture of the skull, clinical signs of skull base lesion, vomiting (more than twice), age of 65 years or older. A moderate risk of cerebral lesion is expected in case of the following symptoms: retrograde amnesia of more than 30 minutes, dangerous mechanisms of the accident (those are for example the collision of a pedestrian with a car, fall from a height of more than one meter, and being ejected out of a car) [38].

Patients having had an accident always receive cranial CT scan (CCT) in the emergency unit if a skull lesion must be considered. 12% of those cases also suffer from midfacial fractures. Only in 16% these lesions are satisfactorily assessed in that way that in the further course of treatment no additional CT scan is necessary. So it is appropriate to indicate also CT scan of the midface during initial imaging diagnostics in order to avoid later time and cost intensive diagnostic procedures [39].

### 2.6 Principles of surgical treatment

The surgical reposition of fractures and osteosynthesis of patients with midfacial fractures is typically performed under general anesthesia. Only a closed nasal bone reposition or the treatment of an isolated zygomatic bone fracture can sometimes be performed under local anesthesia, if needed with sedation [40], [41], [42].

For therapy of central midfacial fractures with fractures in the area of the nasal skeleton and the mandible with teeth, the decision where to place the tube for intubation anesthesia may be difficult [13], [43]. On the one hand, the nose has to be freely accessible for reposition, on the other hand the control of occlusion is essential for correct reposition of the mandible. Often a mandibulo-maxillary fixation is performed at least for the duration of surgery in order to secure the occlusion. So it may be necessary to change the position of the tube during surgery from a nasal to an oral position or vice-versa. Other possibilities consist of positioning the tube behind the teeth or perhaps even through dental gaps, to create a submental exit, or to initially perform tracheostomy [13], [44], [45], [46].

The treatment of a patient’s fracture with a midfacial trauma is usually performed in supine position [22]. In cases of intraoperative navigation, fixation of the head may be necessary [47]. For intraoperative radiological diagnostics, the application of a carbon head cup is recommended.

In the context of surgical therapy of midfacial fractures, standard surgery sets are used. They are completed by specific osteosynthetic material [13], [48]. It consists of typical mini- and micro-plates of different shapes with according osteosynthesis screws of different length [49]. The plates and screws consist of titanium and are stored in sets that can be re-sterilized. Beside the traditional plates, also titanium meshes or pre-shaped plates such as the 3D titanium orbital plate are applied in special cases [24], [25]. Especially for the reconstruction of the orbital floor, also other alloplastic material as polydioxanone (PDS) or porous polyethylene are applied [50]. Absorbable osteosynthetic material is used in children because of possible growth disturbances and migration of the plates [19].

For naso-ethmoid fractures, absorbable materials are not established up to now. There is currently no literature allowing a comparison to titanium systems. In pediatric fractures they are applied in selected cases [51]. The arguments against absorbable materials are well known and yet undisputed: The material thickness is too high in the central midface, the infectious rate is higher compared to titanium, and the duration of surgery is longer [49], [52], [53].

Generally, the bone fragments should be repositioned in their anatomically correct position and secured safely. The objective is the reconstruction of the shape and function of all structures of the midface, as atraumatic as possible [12], [13], [14].

The reconstruction of the correct occlusion is one of the most important objectives of surgical treatment of midfacial fractures affecting the teeth-bearing parts of the midface [54]. Hence, in many cases at least a temporary mandibulo-maxillary fixation (MMF) via orthodontic archwire plastic splints or MMF screws is required to secure the occlusion. In the context of fracture treatment, all fractures must be identified before starting with the first osteosynthesis [54]. Otherwise fixation of fragments in wrong position might happen. The number, size, and position of the single plates depend on the anatomical and biomechanical properties of the individual fracture situation. Basically, however, the plates are positioned alongside the horizontal and vertical pillars of the midface. For osteosynthesis, generally mini- and micro-plates are applied that are fixed with monocortical osteosynthetic screws.

The reposition and osteosynthesis of an extended midfacial fracture generally starts with the reconstruction and securing of the occlusion as a reliable reference for all further steps of fracture treatment [54]. Afterwards, the treatment starts in the superior level in reference to the skull base and to intact bone structures, the fragments are repositioned and fixed with mini-plates. Then the reposition continues one level more inferior (“top down” procedure) [13], [18], [54]. By means of this procedure, an outer frame of the midface consisting of the zygomatico-frontal transition, the zygomatic bones, and the zygomatico-maxillary transition is established in first place [13]. After that, after mandibulo-maxillary fixation of the correct occlusion, the central parts of the midface (naso-ethmoid complex, Le Fort I and II level) and the orbit are treated [13]. Each wrong positioning of the bony structures in the area of the midface leads to inharmonic appearances of the facial soft tissue and thus to poor esthetical results. Additionally, massive functional disturbances such as impaired occlusion or vision may be the consequence [54].

### 2.7 Antibiotic prophylaxis of midfacial injuries

The anti-microbial therapy in case of proven infection is not dealt with in this paper. In the following, only statements on the prophylactic application of antibiotics in case of facial lesions are described. Finally, it is the question to what extent prophylaxis is useful in order to avoid infection on the one hand and on the other hand not to cause germ selection and avoid side effects of antibiotic therapy [55].

The perioperative application of antibiotics corresponds to current treatment standard for most surgical interventions in ENT and maxillofacial surgery. For treatment of midfacial fractures, at least 2 of 3 indications are fulfilled that are described in the AWMF guidelines on perioperative antibiotic prophylaxis [55], [56]. It is considered as proven that antimicrobial prophylaxis applied 2 hours before intervention leads to a lower infection rate in the surgical field than applying the drug off this time slot [57]. In order to guarantee the effect of the antibiotic, the first application has to be performed as early that the therapy effective tissue level is achieved before beginning of surgery up to the end. For interventions up to 2 hours, a single dose is sufficient [55].

A current investigation performed by Lauder and co-authors shows that the additional application of antibiotics outside the perioperative time span does not reduce the rate of postoperative infections [58]. Even in cases of extended soft tissue lesions and multiple open fractures, a continuous prophylaxis is not appropriate. The possibly immediate application of a cephalosporin is useful to reduce the infection rate, further medication does not contribute advantages [59]. Those results are confirmed by other authors. Soong et al. found no difference in one-day or 5-day application of antibiotics with 625 mg amoxicillin and clavulanic acid for one day compared to the 5-day therapy so that the authors recommend perioperative antibiotic therapy.

### 2.8 Interdisciplinary cooperation

The vast majority of lesions of the midface and neighboring structures such as the anterior skull base and orbit content have to be cared for by more than one medical discipline [60]. Regarding diagnostics and therapy, the expertise of radiologists, anesthesiologists, neurosurgeons, intensive care specialists, ophthalmologists, maxillofacial surgeons, and otolaryngologists is required in order to achieve an optimal outcome of trauma treatment [13], [61], [62]. A consecutive procedure strictly divided according to the different disciplines is not helpful for patients with extended midfacial trauma and involvement of neighboring crucial structures. Unnecessary multiple anesthesia, timely delay, and relevant impairment of the course would be the consequences. Instead, the team responsible for the patients should consist of all experts that are necessary for comprehensive treatment. Only in this way, the individual and optimal treatments can be correctly planned and performed. The interdisciplinary coordination regards the necessary diagnostics, the surgical approaches, the provision of instruments and additional technical devices, the sequence of fracture therapy, and the postoperative care. For best possible patient’s care and a smooth management, comprehensible and clearly defined procedures are necessary. They should be appropriate and simple, in particular, there should be no space to enlarge the own discipline in the disadvantage of neighboring disciplines.

## 3 Central midface

### 3.1 Fractures of the nasal bone, the nasal septum, and the naso-ethmoid complex

#### 3.1.1 Fractures of the nasal bone 

Injuries of the external nose and nasal bone fractures are the most frequent lesions of the midface [63], [64]. The gender ratio is 12–37% of female and 63–88% of male patients with a median age of 27 years [65], [66]. Assault and battery is the most frequent reason of fractures in 80% of cases [66]. The fracture line mostly extends vertical to the nasal axis and is often (in 78.8%) associated with fractures of the bony and cartilaginous septum [67]. Septum fractures for their part, are the origin of nasal septum hematomas that may result in severe complications. They have to be considered as surgical emergency therefore [65]. Based on hematoma, infections lead to septal abscesses. Rapidly, necrosis of the cartilage may develop, a possible sequalae in such case is a saddle nose deformity with functional impairment [30]. In order to avoid those complications, the drainage should by performed within 24 hours [67], [68]. A broad spectrum antibiotic drug is applied for anti-infectious therapy until the microbiological findings are at hand. The most frequently found germ is *Staphylococcus aureus* followed by *Haemophilus influenzae*, *Streptococcus pneumoniae*, and β-hemolytic streptococcus of group A. As antibiotics, penicillin and clindamycin are recommended [69]. If necrosis of the septal cartilage is already found, an immediate reconstruction with autologous concha cartilage should be performed in order to avoid deformity of the external nose [65], [70].

Even in simple fracture types exact diagnosis poses a challenge. The clinical examination is of major importance. Typical symptoms are epistaxis, soft tissue swelling, hematoma, and nasal obstruction. Especially in cases of slowly developing nasal obstruction, septal hematoma or septal abscess must be suspected early. Palpation helps to reveal classical fractures. If swelling impairs finding an abnormal mobility and crepitation, the examination has to be repeated after local decongestion [67]. Lateral radiography does not provide exact results and is not recommended [68], [71], [72]. Pediatric nasal injuries are assessed by the same procedure. Also in this context it is important to rely on the exact clinical examination and not only on radiological imaging [65], [73], [74].

The intensive, also radiological examination is undisputed in case of severe injuries. It is more difficult to make a decision in cases of small head injuries (defined as Glasgow Coma Scale 14–15, unconsciousness of <5 min and/or amnesia) that might have led to nasal bone fractures because apparently the benefit of the examination and the radiation exposure have to the weighed out [75]. Vestergaard and co-workers analyzed the management of all hospitals in Denmark that treat pediatric trauma. None of the institutions performed routine radiological examinations [75], [76], [77].

Even for injuries of the pediatric midface that seem to be less important, septal hematoma, septal fracture, and septal abscess must be ruled out – as children with even mild traumatic brain injury may develop a post-concussion syndrome or other neurological complications such as concentration dysfunction, personality disorder, or educational problems in 10–20% of cases [78]. In pediatric noses, the muco-perichondrium only adheres loosely to the cartilage so it may easily be lifted by bleedings. Álvarez observed smaller complications at the nasal septum and the appearance of the external nose in 37.5% of septal hematomas, severe complications in 62.5%. However, following low-grade injuries, septal hematoma or abscess developed in 53.2%. Only 12.5% of the examined children had a septal fracture. All children were treated surgically, in the group of severely injured children more than one intervention were required. Beside esthetical deficits, nasal obstruction is the most frequentlcomplication [79], [80].

Nonetheless, in cases of suspected nasal bone fracture, a lateral radiography is performed in a high number of cases in order to avoid medico-legal problems (Table 2 (Tab. 2)), although the medical colleagues know well about the limited diagnostic value of this examination [81]. Additional examinations are necessary in 52% of simple nasal bone fractures to ensure diagnosis [66]. Other authors report about a rate of 50% of false-negative findings in the lateral radiological imaging [65]. Computed tomography is required to diagnose septal hematoma or to exactly classify fractures [63], [67]. The task force on osteosynthesis does not list radiological diagnostics for simple nasal bone fractures in their recommendations [82]. The significance of careful primary treatment becomes apparent regarding the high rate of secondarily necessary revisions, e.g. rhino- or septo-rhinoplasty. According to the authors they vary between 9 and 50% [64].

The closed reposition from exterior is recommended only, if the longitudinal axis is deviated exclusively, if the fracture is fresh, and the fragments do not overlap. Any other fracture needs to be repositioned in two planes. Septal fractures have to be repositioned openly [74]. Access and surgical technique correspond to septoplasty. The surgical steps are performed in the following order: external nose in first place followed by the septum. In pediatric patients, the objective is to preserve the connection between the vomer and the cartilaginous septum in the axial as well as frontal level in order not to touch the growth zones of the inner nose [83].

Nasal packing and nasal protection meshes are used for redressing and protection of the wound. They remain for variable durations. The recommendations extend from one to 14 days for packing and up to 3 weeks for external nasal protection. Ointment stripes [84] are no longer mentioned in current rhinological literature. As the soft tissue swelling may increase again after manipulation, special attention must be paid to the external nasal dressing not leading to pressure damage of the skin.

#### 3.1.2 Naso-ethmoid and naso-orbito-ethmoid fractures

The naso-ethmoid regions structurally consists of horizontal and vertical pillars. In the cranial and horizontal regions, the frontal bone provides the shape, in the caudal-horizontal region parts of the maxillary bone and the zygomatic bone are forming and giving stability to the orbit. Medial is the insertion of the canthal tendon at the bone is of central importance. It provides support to the eyeball, the eyelids, and the orbicularis oculi muscle and is the border to the lacrimal sac. The bony base is the “central fragment” and represents a particular challenge for reconstruction [7], [85], [86]. The lateral pillar consists of the zygomatic, frontal, and maxillary bones. From there, surgeons usually start to reconstruct the midface for fracture reposition. Based on this reference, the intervention proceeds in medial direction.

Frontal trauma of heavy impact (3.6–7.1 kN) [87], [88] breaks the nasal bones and the frontal processes of the maxilla as well as the ethmoid cells. In contrast to frequent nasal bone fractures, naso-ethmoid fractures occur rarely with 5–15% of facial fractures. They belong to the most complex cranio-maxillofacial fractures and every insufficient treatment may lead to severe esthetical and functional impairment. They rarely occur as isolated fractures – they are mostly part of pan-facial fractures [85]. 

The naso-ethmoid complex is shifted in posterior direction and the nose loses its projection. Because of the shear forces to the orbit, simultaneous damage of the orbital cavity is likely. If the canthal ligaments are detached from their periostal base or severed, there is at least a cosmetically disfiguring result of the orbital septum (telecanthus) or even a functional deficit of the eyelid system [89] which may become obvious as impaired eyelid closure or disturbed lacrimation. The small lacrimal ducts can be injured by direct trauma, the big lacrimal ducts are mostly injured by indirect trauma [90].

The classification established by Converse classifies trauma according to the direction of impact. Grade I means that the nasal bones are hit by an impact from the frontal inferior side and move in dorsal and lateral direction cutting the canthal ligaments. The fragments are pressed into the interorbital space. In case of grade II, the impact comes from a frontal direction. The canthal ligaments still adhere to one fragment and are drawn by muscular traction of the orbicularis oculi muscle in a lateral direction. The widely accepted classification according to Markowitz is based on the condition of canthal tendon mainly [88]. The difference is still made between uni- or bilateral fracture and regarding an extension of the trauma into other regions (Table 3 (Tab. 3)) [74], [91].

As for other types of fractures, all classification systems have particular advantages, however, they often do not exactly meet the individual fracture mechanism. In cases of naso-ethmoid and naso-maxillary fractures the possibly most exact description of the fracture course based on CT diagnostics was established. For example, naso-maxillary fractures concern the same anatomical region as the traumas classified according to Markowitz, but the canthal ligament is intact [92].

The symptoms of naso-ethmoid fractures include significant swelling of the soft tissue envelope, saddle nose deformity, epiphora, olfaction disorder, epistaxis, monocular or binocular hematoma, hyposphagma, CSF leak in case the cribriform plate is affected, diplopia, and nasal obstruction. Infection in the further course may develop as well as atypical facial pain [18]. The swelling makes diagnosis based on clinical criteria difficult so that injuries may not be diagnosed to their full extent.

Clinical examination reveals the abnormal mobility of the bone fragments, the focus hereby is placed on the central fragment. Rounded canthi (“cow’s eye”) are a first sign of a dislocated fracture. The integrity of the canthal ligament may be tested by pulling the eyelid in a temporal direction and checking wether or not the central fragment is mobile [93]. If the intercanthal distance exceeds 35 mm, rupture or dislocation of the canthal ligament must be taken into account. For the exact presentation of the fracture courses, computed tomography is essential. With slice thickness of 1 to 2 mm, the reconstructions of the 3-dimensional conditions allow evaluation and planning of surgery [85], [89]. Only if fragments are not mobile on palpation and no dislocation is found, conservative therapy may be performed. All other fracture types require open reposition and fixation. The choice of access depends on the extent of the trauma. Up to 4 incisions may be needed to achieve a satisfactory overview: the coronal incision, the sulcular vestibular incision, the approach to the orbital floor, and if needed a small access medial to the nasal dorsum [94]. This medial incision at the radix allows a easier reposition of the central fragment for wire osteosynthesis than the coronal incision [95]. The wire osteosynthesis is preferred in comparison to plate osteosynthesis because the central fragment is small and mobile [96]. Engelstad presented a method of reconstruction that provides several advantages in case of unilateral trauma. The canthal ligament is taken with a wire or a non-absorbable suture and fixed to the medial orbital rim by an osteosynthesis plate [86], [97]. If the tendon of the canthal ligament is completely detached from the bone or if the bone fragment is too small to be taken by wire osteosynthesis, a suitable bone fragment is chosen that can be brought into contact with the supra- and infraorbital bone bridge [85]. Prior to reconstruction of the naso-ethmoid complex, the frame consisting of the frontal and maxillary bones is reconstructed [88], [89].

The treatment with external redressing of the fragments that was formerly applied, was replaced by internal fixation [52], [93], [98].

In cases of less extended fractures in the central midface, individually designed local approaches may be sufficient. These options are discussed when already a traumatically caused access is present. In order to treat canthal ligaments that are detached from the bone or extended fractures with dislocation in a functionally and esthetically satisfying way, mostly an approach via coronal incision is recommended [2], [93], [98]. The soft tissue envelope especially in the area of the supra-alar crease is plugged in its original position by pressure bandage [85].

The drainage pathway of the frontal sinus can be impaired by dorsal dislocation of cranial fragments. Disturbed drainage pathways are the sequel which is preferably treated by median drainage of the frontal sinus [74]. The transposition in a posterior direction of the nasal bones is associated with a dislocation of the quadrangular lamina and the bony-cartilaginous connection at the rhinion [99]. If a comminuted fracture is found of which the fragments cannot be assembled in a stable form, the reconstruction with bone transplantation from the skull is a safe measure to avoid soft part shrinking of the nose. This would then be secondary and only difficult to treat [94]. Beside cranial bone, also transplantations from the rib are appropriate. In the field of rhinoplasty, much experience could be gained from this procedure, but rib transplantations are more likely to deform [100] and cannot be screwed as easy at the nasion as “calvarian cantilever grafts” taken from the calvaria [88].

### 3.2 Injuries of the frontal sinus

#### 3.2.1 Epidemiology, pathogenesis, classification

Injuries of the frontal sinus contribute to maxillofacial trauma, with 5–15% [101], [102]. They occur only after impact of significant energy (1–2.17 kJ) or a force of 3.6 to 9.0 kN [103], [104], [105], [106] because the bone of the anterior wall represents a strong horizontal barrier. (*The values given in the literature can only be transferred to the SI system in a limited way. The units in the primary literature are sometimes chosen unconventionally.*) Its thickness is up to 12 mm, the posterior wall is relevantly thinner with 1.9 to 4.8 mm [107]. The most frequent origin are traffic accidents (31.7%), sports accidents (28.0%), work-related accidents (20.1%) and violence (3.7%). Predominantly young men aged between 20 and 30 are affected years [62], [108], [109], [110], [111]. Pollock found in an investigation of 154 patients in Kentucky who had an accident that the origin was a traffic accident in 50%, 81% were male patients, the median age was 34 years [112]. 66% of the patients had additional craniofacial fractures, 35% had accompanying orbital fractures [113]. External injuries are observed in 50%, in 25% of the cases an impression is visible in the acute stage. Concomitant symptoms are epistaxis, impaired vision, edema, paresthesia, and pain on a regular basis [103]. Because of its stability, the anterior wall of the frontal sinus resists better than every other craniofacial bone to external forces [62], [105], [114]. In one third of cases it is the only bone concerned. In only 5–11%, the posterior wall is solely fractured [105]. In 38.4–80% of cases, the posterior wall and the frontal recess are affected as well, in 70.7% the drainage pathway is concerned whereas in 67% obstruction is observed and in 16–30% additional CSF leakage occurs [48], [101], [110], [112], [115], [116]. Trauma to the nasofrontal duct was found in 29.2% of cases by Dalla Torre et al. [109]. If complications occur, they concern the frontal sinus drainage pathway in 95% [116]. The ratio of fractures through the lateral floor of the frontal sinus compared to fractures through the medial floor of the frontal sinus is 3:1. Pollock recommends high resolution parasagittal CT scans for diagnosis of the drainage pathway and the floor of the frontal sinus [112].

A series of classifications was suggested, however, none of them could really be established [19], [117], [118], [119]. In contrast to merely anatomically oriented classifications, systems are preferred that contain information about the therapeutic procedure [119]. Fractures of the floor of the frontal sinus and the fronto-ethmoid transition are radiological hints to involvement of the drainage pathways.

#### 3.2.2 Diagnostics

The clinical diagnosis of frontal sinus fractures remains incomplete and is additionally impaired by initial swelling. Among the symptoms, the following findings are observed: epistaxis, anesthesia and dysesthesia in the field of the first branch of the trigeminal nerve, rhino-liquorrhea, subconjunctival ecchymosis, orbital emphysema, soft part defects, and bone dislocations. In order to be able to decide about an appropriate therapeutic regime, the anatomical aspects must be examined based on CT diagnostics [105], [111], [113], [120]. The standard of imaging diagnostics is computed tomography. Whenever possible in high resolution and submillimeter technique to enable multiplanar reconstruction [103]. The rhinosurgeon is used to evaluating CT scans of the paranasal sinuses and would apply the same technique as for inflammatory diseases [121]. Patients with multiple injuries undergo spiral CT examination that is standardized for trauma patients [113], [122]. If sections of less than 2 mm are acquired and 3 planes are provided, a correct statement about the injury of the drainage pathway can be made in 96% of the examined cases [123]. The integrity of the frontal recess, the anterior wall of the frontal sinus, the posterior wall, and the dura are in the focus of interest. The diagnostics of suspected or existing dura fistula with CSF flow is described in one of the following chapters on frontobasal injuries.

#### 3.2.3 Indications of surgery and contraindications

The objectives of surgical treatment are, according to their significance, the therapy of dura fistulas and thus the separation of the neurocranium from the nose and the facial skull. It is important to avoid early and late complications, especially complications of the central nervous system. The outline of the forehead should be reconstructed simultaneously with taking care of the physiological function [106], [111].

The recommendations were adapted to modern criteria of frontal sinus surgery. The technical conditions have changed fundamentally due to advances in endoscopic sinus surgery [124]. Since 1987, obliteration as primary intervention for frontal sinus fractures has been questioned [117], [125].

In every case, the wound has to be cleaned, foreign bodies must be removed and cerebral lesions have to be treated [110]. Obliteration and cranialization of the frontal sinus are still a frequently performed therapy of fractures of the medial floor of the frontal sinus and of complex fractures that also concern the posterior wall [111].

In order to avoid mucocele formation, surgical interventions need to be accurately planned and executed in case of traumatized naso-frontal transition [62], [109]. Kalavrezos, B. Strong, and Koento recommend obliteration of the sinus cavity in case of fracture and obstruction of the frontal recess, whereas Smith et al. and Rice et al. as well as other groups consider wait-and-see strategy [102], [106], [111], [125], [126], [127], [128]. Xie et al. evaluated 4,000 patients’ charts with frontal bone and cranial fractures over a time period of 30 years. As described by Wilson and co-workers, reconstruction was the better alternative compared to obliteration and cranialization [129], [130]. The probability that the natural drainage is reestablished or can be reestablished by endoscopic surgery in case of complication is high. Precondition for such a procedure is a sufficient compliance of the patients who would present for CT diagnostics immediately in case of complaints in the area of the frontal sinuses. Patients have to be informed about sinogenic orbital complications and sinogenic meningitis or cerebral abscess. The minority of mucoceles and pyoceles of the frontal sinus, however, are due to trauma [19].

Obliteration would then be reserved to cases where endoscopic procedures fail or the primary damage is so extensive that anatomically correct reconstruction seems to be impossible [110], [127]. Further complications are chronic sinusitis, meningitis, and cerebral abscesses [101].

Fractures of the anterior wall of the frontal sinus are treated only in case a cosmetic deficit must be expected [111]. As of a dislocation of 4 mm, Kim et al. consider the situation as being treatment-worthy [50], [131]. Strong et al. recommend therapy already at a dislocation of 2–6 mm in order to avoid cosmetic deficits later on. Since the morbidity of the treatment via coronal incision or pretrichial approach [106], [126] may exceed the immediate trauma sequel, an endoscopic approach should be considered in these patients. Also the trauma access must be checked because in half of the cases a soft tissue injury is found [103]. There is no indication for obliteration in those injuries [112].

Generally, the external force leads to plastic deformation of the bone. It may be impossible to arrange the fragments in their anatomically correct position [23]. The edges of the fragments are grinded in order to facilitate reposition. For osteosynthesis, titanium plates of different thicknesses [48].

Endoscopic procedures are applied to reduce the considerable rate of complications that are associated with the coronal incision. Especially scars, paresthesia, and alopecia must be taken into account. The approach corresponds to the one of endoscopic brow-lift. Incisions of 2–5 cm in the area of the hairy scalp, 3 cm way off the hairline, are performed to insert the endoscope and the instrument for dissection [101], [102]. The fragments are mobilized via an additional percutaneous access (stitch incision), retrieved via the endoscopic access, and grinded. At the table they are fixed on a microplate before being repositioned. The screws at the skull are tightened percutaneously [108].

It is technically simpler to perform secondary reconstruction of the front with endoscopically inserted transplants made of polyethylene or titanium to bridge the defect. A self-tapping screw fixes the implantation at the bone.

Egemen describes a technique where the visualization occurs via the endoscopic brow-lift and fragments are repositioned via a stitch incision transcutaneously. A screw facilitates the manipulation [115]. Reposition of the fracture in this way can be technically challenging, especially when the fragments are found laterally [103] so that the secondary improvement of the outline is recommended as alternative [127]. Dislocated fragments of the anterior wall can also be treated via a miniaturized access at the eyebrow. The cosmetic outcome is described as satisfactory [132], [133]. Blood loss, long duration of hospitalization, and neurological deficits as they are observed after coronal incisions can be avoided [23], [134].

Molendijk describes another minimally invasive procedure for the treatment of fractures of the anterior wall with 2–3 bigger fragments: osteosynthetic screws with a diameter of 2 mm are screwed transcutaneously into the fracture fragments and manipulation at the screw heads leads to reposition [103].

Extensive reconstructions are possible via a coronal incision with exposition of the whole forehead. The fragments are put into a position that corresponds to the contour of the contralateral side. As frontal force leads to compression of the bony tissue, the fragments can hardly be repositioned and fixed without prior processing for adaptation. Plastic measures at the fragment edges facilitate reposition. Defects that have to be covered in order not to risk deformed frontal contours by sinking soft tissue can be bridged with titanium meshe. Hydroxylapatite is not recommended because of the risk of infection [101].

Fractures of the posterior wall of the sinus are often associated with dural lesions and CSF leak and require special attention. The probability of dural lesions increases with the dislocation of the fragments. If the anterior and posterior wall of the frontal sinus are fractured, in 86.7% also endocranial lesions must be suspected according to Molendijk et al. [103]. In 25% of the patients with frontal sinus fracture, there is a dislocation of more than 5 mm. Hereby the probability of CSF leak is clearly higher than in cases of minimal dislocation [109]. Day and co-workers consider therapy through the cavity as being insufficient and recommend neurosurgical craniotomy [48], [135]. As described in the chapter on fronto-basal lesions, also in the context of ethmoid and frontal sinus fractures the necessity should be discussed to treat concomitant CSF-leakage. Bradley Storng and several other authors recommend conservative therapy of CSF leak for 7–14 days and observe the situation. In 50% of the cases, they found spontaneous closure which they considered as sufficient. Pollock achieved closure of the leak in 38% of his patients by a wait and watch strategy, the other 62% were treated via a coronal incision. Complications of cranialization, which is performed in 35%, amount to 6% [112].

Only in cases of persisting leakage, he performs duraplasty together with obliteration of the sinus. If the dislocation is larger than the wall thickness, the sinus is also obliterated when no dural lesion is present. If the defect expands to more than 25–30% of the posterior wall, cranialization should be discussed according to these authors. Blocking of the drainage pathway that is required in these cases. Various materials are discussed while there is no superiority of one of these. All authors agree to the recommendation to completely remove the mucosa from the cavity and to invert the mucosa of the drainage pathway and to push it in nasal direction. As material for the barrier in cranial direction, bone, bone chips with gelatin, a pedicled periostal flap, or muscle plugs are suggested [112], [136], [137].

The long tradition of aggressive surgery of frontal sinus fractures is based on procedures that were established in the first part of the last century. The main reason to readily obliterate the frontal sinus was the fear of complications such as fulminant sinusitis, development of celes and cerebral abscesses originating from the sinusoidal mucosa [112]. In case of fractures of the posterior wall, it was suspected that the epithelium grows into the endocranium and that mucoceles may develop there. However, it turned out that that mucocele formation did not increase due to damaged drainage pathways [138].

The development in the field of endoscopic sinus surgery questioned the indication of primary obliteration and cranialization. The success of secondary surgery is an important reason why surgeons are more reluctant to fundamentally change the anatomy of the frontal sinuses today [106], [113], [124], [139], [140].

**Figure 4:**
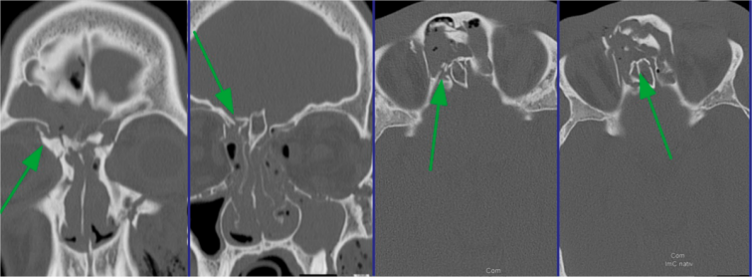
Frontal sinus fracture. From left to right, the arrows mark: the central fragment with base of the canthal ligament and the trochlea, involvement of the drainage, posterior wall of the frontal sinus.

Many authors take their decisions based on the type of lesion of the frontal sinus drainage. In case of radiological evidence of impaired drainage pathway, obliteration or cranialization may be considered rather than preserving the ventilated sinus [116], [123], [126], [141]. If 2 or 3 of the following criteria are fulfilled in CT diagnostics, the probability amounts to 81% that the drainage pathway of the frontal sinus is damaged: 1. Fracture of the floor of the frontal sinus, 2. Fracture of the medial part of the anterior wall of the frontal sinus, and 3. Obstruction of the drainage pathway. In those cases, Yakirevitch argues for obliteration and cranialization [142], [143], [144]. Rodriguez performed follow-up examinations in 857 patients where he had found a complication rate of 9% for obliterated frontal sinuses and of 10% for cranialized frontal sinuses [145], [146] (Figure 4 (Fig. 4)).

The access via an incision medial to the medial canthus with different lengths in cranial and caudal direction is a standard approach to the frontal sinus since the beginning of the last century [147], [148]. Because of the poor view on lateral direction and the risk of scars obstructing the frontal sinus drainage, however, it plays a minor role in the treatment of frontal sinus fractures nowadays.

Strong recommends trephination in order to identify hematomas, fractures, and dura fistulas [101], [126]. One might return that hematomas are not relevant, fractures can be assessed by CT scans with sufficient safety, and CSF leaks cannot be safely excluded.

**Figure 5:**
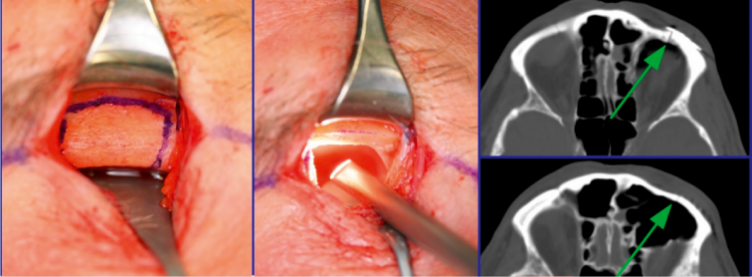
Lateral transorbital osteotomy of the frontal sinus

The coronal incision with partial removing of the anterior wall represents the procedure providing the best overview and highest safety. However, also in this context the negative consequences of scars, risk of infection, alopecia, and longer wound healing must be mentioned in comparison of endoscopic procedures. Fractures and dural lesions near the frontal sinus outlet are covered endonasally according to the standard procedures of functional endoscopic sinus surgery. Since it is not possible to reach fractures in cases of small frontal sinus ostium (<6 mm) [149], lateral of the pupillary line and in the cranial parts of a pneumatized frontal sinus, miniaturized external approaches are described that allow the use of endoscopes and the according instruments (Figure 5 (Fig. 5)).

Small defects no bigger than 5 mm areas within reach can be treated endoscopically. Many authors consider cranialization as not being necessary if the CSF leak is just due to the fracture of the posterior wall of the frontal sinus. The defect is closed by bath plug technique for example. Permanent closure rates of 86–100% are achieved with minimal morbidity.

The data on the number of cranialized frontal sinuses vary according to the reports. Donald considers it a necessary procedure in 13.7%, Pollock performs it in 35% of his followed-up patients [102], [146], [150]. It would be interesting to know if rhinologists, maxillofacial surgeons, and plastic surgeons prefer different procedures that are due to their special training [112], [116], [127].

For obliteration, different materials are recommended. Besides autologous bone [151], [152], [153], e.g. as calvarian bone dust, fat of different origin, temporalis muscle [154], [155] also alloplastic material such as hygroxylapatite [156] and demineralized, attenuated human bone are used [145], [152]. Strong recommends fat taken from the abdomen [101], [126]. Most reports are published for this material and it is the material with the longest tradition. Weber and co-authors report a case series of 59 patients who were followed-up over a period of 12 years. They found mucoceles in 10%. In more than 50% of cases the fat transplant decreased to 20% of its initial amount. The median half-life of the implanted fat was 15.4 months [157], [158]. The best results are reported for autologous bone [159], [158]. Inflammatory foci may hide in transplanted fatty tissue [144], [145]. The complication rate for sclerosed frontal sinuses is stated with 10.4% by Rodriguez. The percentage of obliterated ones amounts to 9%, the percentage of cranialized frontal sinuses amounts to 10%. In his case series, fat and osteoneogenesis as principles of obliteration had the highest complication rate with 22% of the ones obliterated with fat and 42.9% of the frontal sinuses that underwent osteoneogenesis [116], [144]. Complications following frontal sinus injuries occur in 15.2% of cases after 12 months. However, there is a life-long risk of complications if a frontal sinus had to be reconstructed [106], [128], [155]. The highest rate is observed with simultaneous intracranial lesion [109]. IN 11% of the cases, a post-traumatic pain syndrome must be expected [110].

Local osteomyelitis and cerebral abscesses can be a consequence of a lesion of the frontal sinus [19].

Since obliterated frontal sinuses tend to surgery-worthy complications in 5–30% of the cases independent of the primary surgical indication [116], [144], [152], [160], [161], [162], special strategies of endoscopic revision surgery were developed [18], [163], [152]. The new indications for surgery were mainly based on mucoceles, followed by complications due to bone wax, connective tissue, and polypoid mucosa [152], [160]. In this context, the paper by Weber in this volume is referred to.

In the more recent literature, the trend goes to therapy preserving the frontal sinuses [110], [164], [165], [166], [167]. Clinical and radiological controls in narrow intervals are recommended in order to early detect the development of complications and to react accordingly. Even if the complication rate after a very long time is still to be investigated, especially for minimally destroyed drainage the results of conservative therapy seem to be comparable to more aggressive procedures with obliteration and cranialization [113], [125], [168], [169].

#### 3.2.4 Summary

Until the contrary is proven, a dural lesion should be expected in all frontal sinus injuries with fracture of the posterior wall.

The injury of the drainage pathway is considered as central criterion for the indication of aggressive surgery, if required with obstruction of the sinus. 

Even if the rapid development of the sinus surgery provides possibilities of functional preservation of the frontal sinus after trauma, the traditional procedures of obliteration and cranialization are still widespread procedures of primary therapy. Especially in the rhinological literature there is a clear tendency to perform therapy with preservation of the frontal sinus [170]. In patients with reliable compliance, it is recommended to react on complications when they occur [127], [128]. The cranialization of the sinus is reserved to neurosurgical intervention.

### 3.3 Ethmoid and sphenoid sinus fractures, fronto-basal injuries

#### 3.3.1 Epidemiology, pathogenesis, classification

Isolated injuries of the ethmoid sinus are rare [171]. Fractures of the ethmoid cells without concomitant injuries are so rare that data about their incidence and pathogenesis are not found in the literature. If isolated fractures of the ethmoid cells are diagnosed, wait-and-see strategy is justified. Surgical intervention is limited to the treatment of inflammatory complications and marsupialization or resection of mucoceles in the postoperative course [172]. The situation is different if the fronto-ethmoid or maxilla-ethmoid complex is involved. In this context, the preventive surgery may be indicated in individual cases in order to secure the drainage of the frontal and maxillary sinuses [173].

Ethmoid bone fractures typically occur together with extended fractures of the facial skull and are treated in combination with the orbit, frontal sinus, sphenoid sinus, and especially fronto-basal fractures with dural lesion.

Ethmoid bone fractures with involvement of the orbit are usually medial blow-out fractures. When the orbital lamina is fractured, most often also a fracture of the orbital floor is observed. A clinical hint is given when the ab- and adduction of the affected eye is impaired. Often, however, there are no clinical symptoms in the acute phase [30]. An orbital emphysema may develop via the ethmoid sinus, less probably the maxillary sinus. If it appears, van Issum et al. recommend to wait for 10 days before performing surgery so that the ophthalmological diagnostics can be performed in a mostly decongested eye [172].

The most frequent lesions in the area of the ethmoid sinus leading to direct clinical complications are structural disturbances at the anterior skull base. Gjuric and co-workers found in patients of the University Hospital of Erlangen, Germany, the most frequent injuries of the ethmoid roof after trauma, followed by iatrogenic injuries [174]. Other authors confirm this distribution even more clearly (80–90% of traumatic lesions) [175].

**Figure 6:**
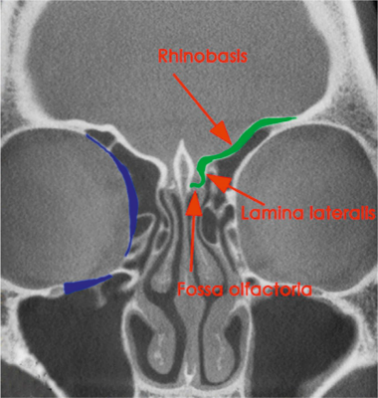
Frequent locations of fronto-basal fractures with CSF leak (green) and medial orbital fractures (blue)

In the following, the ethmoid sinus lesions with involvement of the anterior skull base are discussed. The special focus is placed on lesions with CSF leak. This is defined as an open connection between the subarachnoidal space and the mucosa-bearing parts of the nose and paranasal sinuses [175] (Figure 6 (Fig. 6)).

Injuries of the skull base occur in 43% in the context of complex midfacial fractures [20], [176], in the context of centro-lateral fractures in 25%, of lateral midfacial fractures in 13% [18], and in the context of cranio-cerebral lesions in 21% of cases [177]. Le Fort I fractures are associated with skull base fractures in 1%, Le Fort II fractures in 37%, and Le Fort III fractures in 10% of the cases [178]. One third of the patients with fronto-basal fracture also suffer from dura fistula. The ratio of male to female is 3:1 [179].

Fractures of the anterior skull base primarily concern the cribriform plate and the lateral lamella of the cribriform plate– the thinnest parts of the skull base [18], [31], [180] – and the ethmoid roof. A weak point in the static is the entrance of the ethmoid arteries. The orbits and the adjacent paranasal sinuses are affected more rarely [181].

Fronto-basal dura fistulas develop as sequel of cranio-cerebral injuries with fractures through the anterior skull base mainly in the area of the cribriform plate and the lateral lamina [18]. Here as well, the course of the ethmoid arteries are areas of minor resistance. Since the dura is very thin in the area of the anterior skull base and firmly attached to the bone, CSF fistulas develop easily in this area [182]. There is risk of ascending infections, of pneumocephalus as well as brain tissue herniation into the nasal cavity or the paranasal sinus system.

#### 3.3.2 Diagnostics and surgical indications

##### 3.3.2.1 Clinic

Leakage of cerebrospinal fluid is evidence for fronto-basal injury. The side where the liquor exits, however, does indicate where the actually leak is [183]. Pre-clinical tests are not safe and of low significance. Valsalva or Queckenstedt manoeuvers could increase the CSF flow by the higher intracranial pressure. Valsalva manoeuver should be avoided by all means though as there is risk of pneu-encephalon [184].

Even for experienced rhinosurgeons, the diagnosis of dura fistulas remain challenging. Independent from the etiology, the bacterial meningitis represents the main risk [179]. The endonasal therapy of a CSF fistula has to be postponed until a nearly normal intracranial pressure is found. In order to avoid pneumocephalus, the patient must not blow his nose and nasal cPAP therapy needs to be discontinued. Fatal complications are described in this context. This situation must be discussed with the anesthesiologist with regard to introduction of anesthesia.

**Figure 7:**
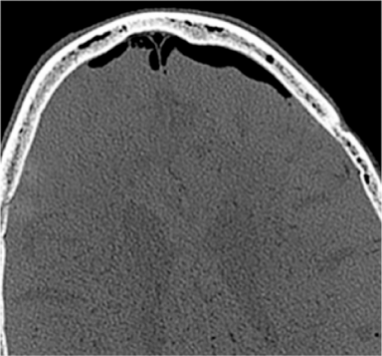
Pneu-encephalon with Mount Fuji sign in the axial CT scan

Increasing headache or neurological decline following an accident iatrogenic dural lesion may indicate epidural air with valve mechanism. Tension pneumocephalus is an acute life-threatening situation. The suspicion should lead to cranial CT scan. Special attention must be paid to the Mount Fuji sign. The subdural air dilates the poles of the frontal brain and widens the interhemispherical gap. The horizontal CT image reminds of the silhouette of Mount Fuji [185] (Figure 7 (Fig. 7)).

##### 3.3.2.2 Radiological procedures

The standard diagnostics in cases of craniocerebral injuries is computed tomography. For examinations in the context of fronto-basal fractures, the coronal CT scan in sections of 1–2 mm is particularly appropriate. Classical radiological procedures do not play a major role [30], [122], [174]. In the acute treatment of patients, the ENT specialist and the maxillofacial surgeon take care that besides the presentation of the endocranium also a high-resolution CT scan of the midface, the skull base, and the orbit is performed to assess neurosurgical aspects. From those original data, all necessary planes may be reconstructed [15], [18], [31], [122], [179], [186], [187].

During routine diagnostics, often fractures of the anterior skull base are overlooked. Perheentupa et al. could reveal overlooked fractures in 23% of the cases in a retrospective analysis of 27 patients with frontobasal injuries [188]. So they recommend a strict algorithm for evaluation that should allow primarily identifying 93% of the fractures.

The posterior wall of the frontal sinus, the lateral and dorsal wall of the sphenoid sinus are best assessed in the axial section. Fractures of the cribriform lamella, the lateral lamina of the cribriform lamella, and the foveolae of the frontal bone as well as the sphenoid planum are most safely diagnosed in the coronal section. Meco also developed a diagnostic algorithm. For traumatic lesions at the anterior skull base, the authors recommend performing a high-resolution CT and the β-trace or β-transferrin test. If the blood testing is negative to cerebrospinal fluid but the radiological diagnostics further support the suspicion of dural leak, the fluorescein test is performed. In a positive case, the proof of treatment-worthy lesion is given, in the negative case, the degree of fracture dislocation gives a hint on the probability of dural lesion. With a dislocation of 3 mm and more, it is expected to be high. In 8 of 12 patients, the authors found a dura fistula intraoperatively. If a pneu-encephalon is found, it is confirmed [179].

Radiological procedures such as cisternography are reserved to exceptional cases and play a subordinate role. Cisternography with contrast agent or radioactive markers is performed as CT examination and allows the presentation of the leakage pathway. It is an option when persisting CSF flow is observed or if the flow can be provoked by Valsalva manoeuver. The examination is associated with the risk of a complication and it is available only in very few centers. For MR cisternography, contrast agent is not necessary. In a strictly T2 weighted examination, an attempt is made to identify the CSF leak [189]. In some cases, rapid spin echo sequences with fat suppression are applied [190]. Both procedures are characterized by weak local resolution [187]. Cisternography with radioisotopes (indium^111^) only have historical significance [122], [191].

##### 3.3.2.3 Confirmed CSF leak and staining

Even though test on glucose is still mentioned in the literature, especially to preclinically prove a CSF leak, its significance is not sufficient to provide safe diagnosis. The glucose concentration in CSF is higher than in nasal secretion so concentrations of 40 mg/dl in the secretion raises suspicion of underlying CSF leak [18], [122].

Eljamel gives the risk of meningitis with 0.62% in the first 24 hours, 9.12% within the first week, 18.82% up to the end of the second week (cumulative values), and 7.2% per week within the first month [192]. 

The indication for surgical treatment is simple when the CSF flow is clinically apparent or if specific CSF protein is found [179]. (*β trace test: The nephelometrically detectable β-trace protein from the prostaglandin metabolism is about 30 times higher in the CSF than in serum. With levels of 6 mg/l and more, CSF is suspected.*) In those cases, a fistula has to be searched and a closure in one of the described techniques is pursued. If the proof cannot be made, either because no nasal secretion can be collected or if the proof was not successful, a dura fistula cannot be excluded [122]. However, even for experienced rhinosurgeons it is not always easy to intraoperatively identify the CSF leak. The situation is particularly difficult when the CT diagnostics reveal endocranial air, but no CSF flow is apparent [20]. The traumatic edema of the mucosa can block a leak so that CSF flow may still occur after the first stage of wound healing [74], [179]. Air bubbles in the endocranial epidural space are a relative indication. If no dislocated fracture is found and the control of the initially negative CSF finding after decongestion of the nasal mucosa remains negative, a surgical exploration is not performed. Intracerebral air, however, confirms a defect of the dura. To find this leak, may be difficult if computed tomography does not reveal a fracture. In those cases, a wait-and-see strategy is justified. If no clinical nor blood testing on CSF is revealed after two weeks, the intrathecal application of Elliot’s reagent of buffered fluorescein solution is an efficient method to stain CSF [61]. Often it is possible in this way to not only confirm a CSF leakage but also the location of the defect.

The procedure was introduced by Kirchner et al. in 1960 [193]. Fluorescein is applied without preservatives. The substance is prepaired prior to each application and buffered in the correct pH area. In literature, there are different data on the concentration, total dose, and the best time of application [179], [194], [195], [196] (Table 4 (Tab. 4)).

As there is no approval for this substance as drug for intrathecal application neither in Germany nor in the US, informed consent has to acquired very carefully. The rules of medical curative trials are applicable. The complications mentioned in literature are headaches, radicular symptoms, temporary pulmonary edema, cerebral insult, hemiplegia, and death. In an investigation of possible complications, Keerl et al. revealed a complication rate in 420 applications from 1969–1997 of 0.1%. Meco et al. performed 900 fluorescein tests with 0.5 ml of a 5% sodium fluorescein solution and observed no complications [179]. The most severe complications are most probably due to incorrect application and do not limit the use of the procedure [196]. Endoscopic examination of the nose is repeated in intervals of 2 hours at least twice after maximal decongestion. In order to be sure not to misdiagnose pseudo-rhino-liquorrhea, the tympanic membranes are examined also. To the author’s opinion, the sometimes recommended application of filters [187], [196] is not necessary because the staining is obvious.

**Figure 8:**
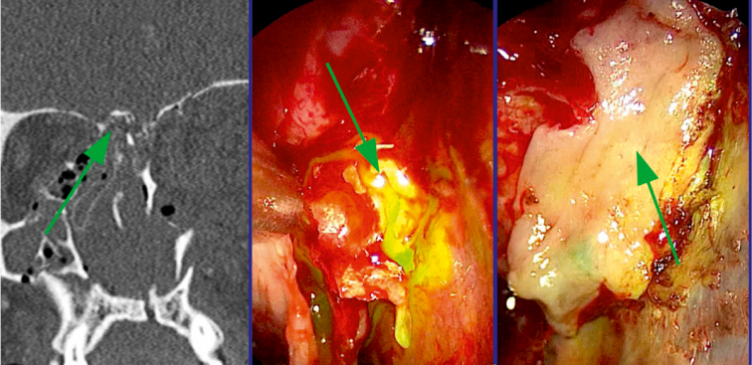
Rhinobasal fractures. Coronal CT scan, CSF leak (fluorescein stain), cartilaginous underlay, mucosal transplantation.

Visible fluorescein at the skull base helps the surgeon to identify the CSF leak in difficult cases. Furthermore it helps to prove wether or not the defect closure is “watertight” [187]. However, a considerable rate (26.2%) false negative results must be expected [194]. Wether this rate is due to too short-term application of the dye prior to surgery, is difficult to assess. With regard to the dynamics of liquor circulation, fluorescin application 24 hours prior to surgery helps to improve results by almost [196] 100% (Figure 8 (Fig. 8)).

#### 3.3.3 Therapy

After craniocerebral trauma with dura fissure, CSF flow usually occurs within 48 hours [197]. Within the first 3 months after injury, 95% of CSF leaks become evident. The clinical diagnosis of CSF leak may be impossible since patients with severe craniocerebral trauma mostly arrive at the hospital intubated, nose and midface are relevantly swollen, and coagulated blood additionally obstructs the nose [18].

70–85% of the defects close spontaneously or after decreasing the CSF pressure by external lumbar liquor drainage, elevation of the upper part of the patient’s body, and other measures to avoid increased CSF pressure [184], [192], [198], [199]. Several authors accept conservative therapy if it is successful within a maximum of 2 weeks. For fractures with proven CSF leak of the skull base in the frontal sinus, Torre and co-workers consider wait-and-see strategy as being justified. The short follow-up time of 12 months is considered as a limiting factor of the study [109]. The clinical and laboratory confirmation of stopped CSF flow is understood as therapeutic success [112], [171], [175]. Since the leak is only closed by a connective tissue layer that is not very resistible or by mucosa and since the dura does not regenerate, ascending infections may cause meningitis in the long-term in 30–40%. 10% of the patients suffering from meningitis die of the sequelae [200]. A secure data situation about the long-term courses of CSF leak does not exist. The difficulty is obvious considering the latency of 2 decades when meningitis can still be associated with trauma [198].

##### 3.3.3.1 Closure of rhinobasal defects

Defects of the anterior cranial fossa with CSF leak have to be considered as possibly life-threatening because of the risk of meningitis. In order to meet this risk, surgical closure is recommended. There is wide agreement that endonasal endoscopic treatment of the lesion is suitable for reduction of the trauma-related rate of meningitis [174], [186], [201], [202], [203], [204], [205]. In this context, the hygienic aspects during surgery at the skull base should correspond to those of neurosurgical interventions. The perioperative application of antibiotics is recommended as explained above (see chapter 2.7) [187]. The endonasal access is considered as safe and therapy of choice. It is equivalent or even superior to other approaches with regard to permanent CSF tightness [205]. A permanent closure is achieved in more than 90% of the cases [171], [186], [194]. For the open access, the closure rates amount to 70–80% [4], [202]. Nonetheless, the open approaches with endocranial, sometimes intradural defect coverage are indicated if comminuted fractures, multiple defects, or severe deformities of the base are found [187].

There are three different methods of defect closure: the sandwich technique, the bath plug technique, and the technique of tissue reinforcement.

Successful defect closures were described for a multitude of applied transplants. Location and size of the defect determine the choice of the method as well as the individual experience of the surgeon [206]. Free mucosal grafts are harvested from the inferior [14], [130] or middle turbinate, the lateral nasal wall, or the nasal septum. The mucosa of the middle turbinate is thin and delicate, it is only suitable for very small and non-dislocated fractures. Mucosa of the inferior turbinate is sufficiently resistible and can be gained in the usually needed quantity [174]. In this context, care must be taken that it can only be put into a planar surface by filleting. Free transplantations form a firm connection with the underlying bone one week after surgery and are replaced by connective tissue after about 3 weeks. Since they shrink, their size is is to be calculated larger as really needed at the time of surgery [174].

Collagen tissue coated with fibrin glue allows tight closure without causing a defect at the turbinates, the lateral nasal wall, or the septum which would be needed to gain a free mucosal graft. Other materials are applied with the same success rates [46], [186], [207]. Small defects up to 1 cm in diameter can be closed effectively with onlay technique. The mucosa must be removed completely from the surface in an area of 5 mm around the defect, in order to create a favorable site for the graft [149], [208]. Autologous and alloplastic materials are at disposition.

**Figure 9:**
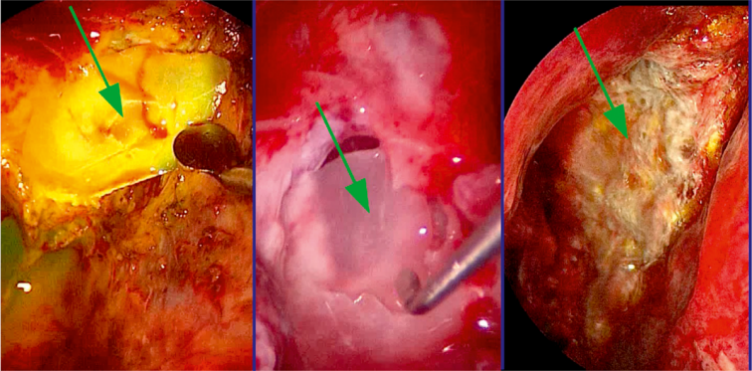
Cartilage chip as underlay, bone chip as underlay, during insertion. Covering with collagen patch.

Middle-sized defects are closed in combined technique: an underlay is put in between dura and bony base, the defect is then covered with an overlay. A discus-shaped underlay cut from septum cartilage is perfectly appropriate. Its edges can be shaped wafer thin with a scalpel so that they easily fit into the defect. The elastic cartilage expands intracranially and thus already allows a tight closure that is additionally secured by an overlay of collagen tissue (Figure 9 (Fig. 9)).

Bone taken from the perpendicular lamina of the septum is also suitable as underlay, however, it is significantly more difficult to position [208].

Injuries of the lateral lamina of the cribriform plate represent challenging technical difficulties for the surgeon. The base has an angle that may make the positioning of a firm underlay impossible, be that cartilage or bone. Hereby, the method presented by PJ Wormald using a fat plug is useful (bath plug technique) [187], [209], [210]. A possible homogenous fatty plug is adapted to the diameter of the defect and cut to a length of nearly 2 cm. An absorbable thread that is inserted along the axis of the transplantation allows compressing the graft intracranially and to create a tight closure even in difficult regions.

The gasket seal technique consists of covering the defect with a soft adaptable material such as temporal fascia or fascia lata and to push it endocranialy [211]. The probability to cause secondary damage seems to be significant. The other, above-mentioned techniques allow a safe closure without putting endocranial structures at risk. We never use the gasket seal technique at the skull base up to the sphenoid planum.

For large defects though, fascia lata is the “workhorse”. It can be applied in single or several layers as intracranial, intradural, extradural, or extracranial graft. It can be harvested in any size needed. Via the same approach fat can be taken for sealing purposes (this fatty tissue, however, is of inferior quality because of its rougher structure compared to fat taken from the earlobe. On the other hand, it can be taken at any quantity). The nutritive situation of the first layer seems to be secured by CSF. In case of multilayer closures, a vascularized transplantat from the nasal septum is helpful. The naso-septal flap pedicled at the posterior nasal artery, a branch of the sphenopalatine artery, has become very popular recently. It can be circumcised anterior to the columella, basal via the nasal floor, if needed even via the lateral nasal wall, and cranial up to the nasal roof. In this way a graft is created that seems to be suitable for all defects [212]. In literature, its application is described as standard procedure for the treatment of frontobasal defects [213] which led to an interesting discussion. It is stated that other procedures are not subordinate, the permanent closure rates are equivalent. A series of situations can be imagined where this flap is inappropriate. Among those, there are pathological processes that include the dorsal septum and the dorsal lateral nasal wall and closures that have to be performed in the anterior cranial area of the base. Furthermore, Solyar recommends careful attention with the use of terms such as “standard” that may have direct medico-legal implications [206].

Since the septal mucosa in the cranial and anterior parts can be very thick and is elevated with the perichondrium, it is likely to roll up. It may be difficult to extend it planar over the defect and to fix it in this position. In those cases it is helpful to delicately incise the perichondrium in longitudinal direction of the flap. This limitation leads to a long period of postoperative wound healing. The large de-epithelized surface of the lamina perpendicularis and quadrangularis needs sometimes several months until crusting decreases and functioning mucosa covers the surface of the surgically exposed septum. If furthermore radiation of the wound is required, the healing phase is again significantly prolonged. A technically simple and very effective method to meet this problem is covering the cartilaginous surface with septal mucosa of the contralateral side [206]. The perpendicular lamina of the septum and the vomer are abandoned, the mucosa is circumcised, remains pedicled anteriorl at the septum and is folded around the dorsal edge of the septum. The free edge is then sutured to the wound edge on the side of the defect.

Large frontobasal defects may lead to herniation of the frontal brain. The pulsation of the brain and the mass movement in case of cough and Valsalva manoeuver would then press against a soft graft and probably detach it from the underlay [187]. The application of rigid grafts such as bone lamellas from the perpendicular lamina is discussed in order to support the soft tissue transplantat. If such a bone bridges the distance between the orbits, an improved stability of the closure can be expected. The use of fibrin glue is recommended by some authors in order to contribute additional tightening, however, Gassner et al. could not confirm any advantage in their case observation [40], [214].

To achieve a tight closure by application of the endonasal procedure, it is required to carefully prepare the underlay for the graft after identifying the CSF leak [187].

The surgeon has to anticipate the shrinking of the graft. The graft can shrink of up to 30% and then with a long delay lead to CSF leakage. For those cases, procedures with pedicled flaps from the forehead and free microvascular transplantats are described [215], [216].

Beside the standard external approach via a coronal incision and elevation of the frontal brain, the anterior skull base can be reached through a subcranical access [61], [217], [218]. Since hereby a broad access to the base is created, also reconstructions after trauma can be performed beside tumor surgery without causing neurological problems because of manipulation at the brain. Especially for multi-fragment fractures and injuries involving nerves, Perheentupa promotes the indication of an open approach [31], [219]. 

Injuries of the roof and the lateral wall of the sphenoid sinus are predominantly observed in cases of severe trauma to the midface [209]. Far laterally located fractures with CSF leak possibly cannot be reached via the transseptal, transnasal, or even transethmoid approach in cases of extended pneumatization. Here, it is possible to reach the target structure via the transpterygoid access [220], [221].

In case of fractures reaching the canal of the carotid artery there is always the risk of developing a carotid cavernous fistula [222].

Iatrogenic dural leaks are technically treated in the same way as other traumatic defects. Their treatment belongs to the repertoire of experienced sinus surgeons. The special situation, also in medico-legal regard, requires special attention. The necessary steps are described in the chapter on complication management.

##### 3.3.3.2 Nasal packing, postoperative care, success rate

In order to press the transplantat to the wound base, to avoid bleedings, and to avoid pneuencephalon when incidentally blowing the nose, packing, absorbable or fragmenting substances or balloons are applied [171]. No scientific evaluations exist for none of the procedures in the context of traumatology. For more details, the paper on R. Weber in this issue is recommended.

In about 90% of the cases the closure is successful after the first endonasal approach [171], [223], even 97% including revision surgeries. In a meta-analysis of 1,778 surgeries of dura fistulas, Psaltis and co-workers found a complication rate of only 0.03% [204]. Another meta-analysis published by Hegazy et al. reported about a very low number of complications. Meningitis was observed in 0.3%, brain abscess in 0.9%, subdural hematoma in 0.3%, disturbed olfaction in 0.6%, and headaches in 0.3% [171]. The complication rate for the endonasal approach is lower than for the transcranial one with 3.2 vs. 12.9% [40], [187], [205], [207]. Recurrences are expected after a median follow-up time of 4 years [40]. The special situation in children was evaluated by Di Rocco in 2010 [14], [188].

Traumatic encephaloceles and meningoceles are rare. In the area of the anterior skull base, the surgical treatment is technically easy. Here, the diagnosis is the relevant step for therapy. Celes may be easily misinterpreted as lesions of the ethmoid sinuses. If they are not diagnosed correctly, a usual ethmoid sinus surgery may lead to complications. In cases of defects of the bony base always a cele must be suspected. The glioma tissue in the encephaloceles never has function and is removed with the cele sac. The surgical principle for both pathological lesions is the same.

##### 3.3.3.3 Antibiotic therapy

The perioperative application of cephalosporin e.g. is a widely accepted procedure [187]. In a meta-analysis Hegazy et al. found the perioperative application of antibiotics in 94% of the duraplasties [171]. Furthermore, the local application of clindamycin in the nose during surgery is recommended. For long-term application of antibiotics for prophylaxis, different time spans are mentioned in the literature [224], [225]. However, meanwhile there are more doubts regarding prophylactic application of antibiotics [226] so that it is no longer recommended [226], [227].

##### 3.3.3.4 External lumbar CSF drainage

In order to reduce the CSF pressure on the transplantation after frontobasal duraplasty and to thus favorably influence the healing, different authors recommend external lumbar CSF drainage [171]. Other authors do not see any difference in the rate of inconspicuously healed duraplasties [175], [223]. A clear definition of the indication was not established up to now, moreover the application seems to depend on the preferences and the experience of the surgeon. An indication that has a clear logic beyond vague recommendations such as “larger defects” or “difficult graft site” are cases of hydrocephalus. This diagnosis seems apply for unsuccessful defect covering and benefits from CSF drainage [171].

CSF should be drained with 5–10 ml per hour. The position of the drainage sac must be carefully controlled. If it is too high, air may ascend retrograde and a pneuencephalus develops. If it is too low, too much liquor is drained and pinching may occur. Some authors recommend drainage for 120 hours. However, there seems to be no clear advantage of drainage lasting for more than 24–48 hours. Strict bedrest should be kept so that no CFS pressure peaks occur [187].

Acetazolamide reduces the CSF production by 48% and so it is an interesting pharmacological option to reduce the CSF pressure. If this possibility is an alternative to lumbar CSF drainage that should be considered seriously in the patients described here, has not been examined [228].

#### 3.3.4 Summary

Skull base fractures with CSF leak pose a high risk of ascending meningitis with neurological defects. Even after up to 20 years, cases of late meningitis after frontobasal fracture are described so that the indication to surgery can also be made if CSF leak spontaneously stops.

Iatrogenic dura leaks are technically treated in the same way as other traumatic defects. Their treatment belongs to the repertoire of an experienced sinus surgeon.

### 3.4 Orbital fractures

#### 3.4.1 Epidemiology, pathogenesis, classification

Fractures of the orbital floor occur either in the context of fractures of the zygomatic bone complex and other midfacial fractures or as isolated findings. In case of an isolated fracture of the orbital floor bony fragments are dislocated caudally into the maxillary sinus [13]. In case of relevant defects of the orbital floor, periorbital tissue prolapses from the orbit into the maxillary sinus. Because of the loss of periorbital tissue, the bulb sinks back into the orbit. In case of bigger defects of the orbital floor, even dislocation of the bulb into the maxillary sinus may occur [229]. 

#### 3.4.2 Clinical symptoms

Clinical symptoms of orbital fractures are hyposphagma, epistaxis, sensitivity disturbances in the area of the infraorbital nerve, enophthalmus, hypophthalmus, diplopia, disturbed eye motility, rounded canthus (cow’s eye) in case of ruptured canthal ligament [18]. The symptom of black eye can be caused by conducted bleedings from the depth of the orbit or by superficial ecchymosis of the eyelids. The symptom alone is not a direct hint of a fracture. However, in 68% of the cases, a radiologically detectable fracture must be expected. 

The following symptoms and situations lead to an emergency situation: partial or complete loss of vision, massively increased intraocular pressure, exophthalmos with hint of acute tumorous lesion within the orbital pyramid (retrobulbar hematoma, emphysema), severe dislocation of the orbital content (e.g. into the maxillary sinus), pinching of the ocular muscles (especially in children, “trap-door” effect).

#### 3.4.3 Radiological diagnostics

For radiological diagnostics of fractures of the orbital floor 2-dimensional radiographs were performed traditionally. Prolaps of orbital fatty tissue into the maxillary sinus is visible as hanging drop in typical imaging of the paranasal sinuses. In order to asses the exact extent of a defect, multiplanar CT scan or cone beam imaging is recommended [54]. 

#### 3.4.4 Indication, therapy

Fractures without dislocation are only treatment-worthy when there is a hint of space-demanding intraorbital bleeding or compression syndromes of the nerves. A fracture can lead to an orbital compartment syndrome with vital threat to the eye even without dislocation. The temporary compression of the 2^nd^ branch of the trigeminal nerve or the optic nerve can cause temporary or persistent damage [54], [230], [231]. If these symptoms are missing, inspection of the orbital floor is not performed so that risks of surgery do not materialize. In this context, especially the damage of the infraorbital nerve and the optic nerve must be mentioned as well as persisting edema of the lower eyelid, impaired vision (occurrence of diplopia caused by bleeding or swelling), ectropion or entropion, and even blindness [20], [30], [232], [233]. The decision of conservative therapy is made on a safe diagnostic basis because secondary corrections of consolidated contour or volume changes in the area of the orbit are difficult and often they are not completely possible [13].

In case of impaired vision due to intraorbital increase of pressure, an immediate intervention is required. The first measure is lateral canthotomy and cantholysis. If the pressure relief is insufficient, additional measures must be taken [234], [235].

Bleedings and edematous swellings within the orbit that is encompassed by the orbital septum in anterior direction lead to disturbances that mostly proceed according to the following rules: disturbed red color perception, loss of vision, restricted visual field. Furthermore the exposition of the orbital floor is indicated in cases of functionally significant defects, mechanical impairment or pinched optic muscle, enophthalmos, and persisting diplopia. Indications of immediate surgery are: enophthalmos, sunken bulb, oculo-cardial reflex that does not improve, and white-eyed blow-out fracture. Burnstine recommends surgery within 2 weeks in cases of diplopia with positive force-duction test, symptomatic enophthalmos, radiologically proven pinching of the orbital content, and fractures that exceed a size that enophthalmos becomes likely [18], [232].

The surgical access to the orbital floor is usually performed in a transconjunctival or transfacial way in the area of the lower eyelid. By means of this incision, a good exposition with the possibility of extension and favorable postoperative scarring is possible. In rare cases, it is helpful to expose the orbital floor via the maxillary sinus [12]. The endoscopic diagnostics is applied in order to identify the dorsal edge of the fracture of the orbital floor, to position a balloon transnasally, to reconstruct the medial wall with a transconjunctival approach, and to reconstruct transantrally the orbital floor with titanium meshes, polyethylene, or absorbable sheets. The most frequently used access is performed via the oral vestibule [23], [233].

After exposition of the orbital floor, the fracture is repositioned and if necessary it is fixed with an osteosynthetic plate.

In case of defects of the orbital floor, the prolapsed orbital tissue is repositioned in first place. Hereby the peculiar anatomical shape of the floor has to be taken into account. In a parasagittal plane, the contour of the floor describes a typical S shape. This course of the floor must be reconstructed in order to correctly reestablish the anatomical shape and the volume of the orbital skeleton [54]. The support of the orbital floor with a balloon inserted from the maxillary sinus (Milewski antral balloon) or with consecutive ointment packing [84] is no longer recommended.

In the AO Surgery Reference, the special significance of the transition zone from the orbital floor to the medial orbital wall and the most distally located parts of the orbital floor are emphasized [54]. In some cases decompression of the infraorbital nerve is required to free the nerve from dislocated fragments.

A variety of materials are recommended for reconstruction of the orbital floor, [12], [50]. Among those, there are autogenic bone, alloplastic materials such as sheets made of polydioxanone (PDS), polyglycol polylactid and porous polyethylene as well as titanium mesh [13]. For very large and complex defects, also pre-shaped titanium mesh is applied. Their design is based on average measured values of the normal population. If they fit well, they may reduce time of surgery significantly.

Fractures of the orbital roof usually remain without consequences unless bone fragments pierce into the orbit. However, cases of oculorrhea are described where liquor is pressed from the subarachnoid space of the frontal brain via a dural fistula into the orbit and causes a pseudo-meningocele in the eyelid. In rare cases, liquor may find its way via the conjunctiva and thus appear as tears [203].

Fractures of the ethmoid sinus and the orbital lamina may lead to diplopia at temporal gaze by pinching the medial rectus muscle. They are identified by radiography and forced-duction test. Surgery is indicated [236].

In any surgery of the orbital floor, the forced-duction test ensures that no tissue incarcerations are overlooked. Pre- and postoperatively controls of vision are recommended.

Injuries of the ocular bulb as well as direct lesions of the external eye muscles are not focused on in this paper. They are dealt with in ophthalmologic literature. Rhinologists and maxillofacial surgeons caring for trauma patients, however, have to know about the different lesions that may follow even in cases of externally intact eye. Beside luxation of the lens [237], in particular also retinal detachment must be mentioned [238].

## 4 Le Fort fractures

### 4.1 Epidemiology and pathogenesis

Le Fort fractures belong the central (Le Fort I and II) and centro-lateral (Le Fort III) fractures of the midface [13], [18], [17]. Isolated Le Fort I, II, and III fractures are very rare [54]. The classical description of Le Fort fractures is based on a symmetric arrangement of the fracture lines on the right and left side. In reality, however, the various fracture patterns occur in different combinations. They can also appear as isolated findings or in combination with other bony lesions of the skull which lead to different fracture patterns. If sagittal or transversal fractures of the maxilla are observed additionally, the fracture patterns become even more complex [13].

The reason for central or centro-lateral fractures is a direct trauma with high impact to the maxilla (Le Fort I fracture) or to the whole midface in most cases (Le Fort II/III fractures) [117]. Etiologically, traffic and sports accidents as well as interpersonal violence are on top of the list of different trauma situations [111], [118], [119].

### 4.2 Clinical symptoms

Unspecific clinical symptoms that are observed in all three Le Fort fracture types are lesions of the skin and soft tissue, swellings, and hematoma of the midface [14]. A typical symptom is also bleeding from the nose because of involvement of the nasal septum and the mucosa of the paranasal sinuses. Another important and frequently found symptom is the impaired occlusion as all Le Fort fractures may dislocate the maxilla. The dislocation occurs in distal direction mostly often and can be even increased by traction of the pterygoid muscles [17]. Because of early contact in the area of the molars, a frontal open bite may result.

In cases of Le Fort I fractures, the maxilla is mobile typically. Pressure sensitive steps can be palpated intraorally in the area of the paranasal pillars and the zygomatico-alveolaris crest on both sides. Bleedings in the area of the mucosa of the maxillary vestibule may be another sign of fracture.

Binocular hematoma is typical for Le Fort II fractures [14]. Palpable steps are found in the area of the intraorbital rim and intraorally in the area of the zygomatico-alveolar crest. Damage of the infraorbital nerve regularly leads to disturbed sensitivity in the area of the frontal teeth, the upper lip, the cheek, and the skin of the lateral nose. If the central midface is dislocated in dorsal and caudal direction, the typical appearance of “dish face” with missing projection of the nose is found. Rhino-liquorrhea indicates involvement of the anterior skull base.

Le Fort III fractures are characterized by separation of the facial skull off the skull base. Typical symptoms are massive swelling and bleeding from the nose and mouth, binocular hematoma, flattening and broadening of the midface, disturbed occlusion, palpable steps latero-orbital and frontal as well as rhino-liquorrhea. The orbit is always involved. Disturbed sensitivity may concern all branches of the trigeminal nerve because of an involvement of the skull base [17].

### 4.3 Radiological diagnostics

Current standard in imaging diagnostics of midfacial fractures is computed tomography. Only the assessment of the fractures in all three planes exactly reveals the courses of the fractures and relevant concomitant lesions [14], [54]. The sections of CT diagnostics should be 2–3 mm or less for midfacial fractures and 1 mm for orbital fractures [54]. Cone beam tomography (CBT) is generally also suitable to accurately depict the fracture courses of midfacial fractures in 3 dimensions. However, this technique still has relevant disadvantages for an assessment of soft tissue lesions. Nonetheless, CBT is widely accepted because of low radiation exposure uncomplicated intraoperative and postoperative application [31], [139], [161].

In all Le Fort fractures, the fragments may be simply fractured or comminuted [54].

### 4.4 Surgical indications

Le Fort fractures are treated either in a closed or open way. Depending of the degree of dislocation, the extent and pattern of the fracture, the type and severity of concomitant lesions as well as clinical symptoms, the indication of immediate therapy is made. Even if no emergency indication is apparent, the surgical treatment should be performed within at least 2 weeks. The interval between 7 and 10 days is considered as optimal because at that time typically the swelling is clearly reduced [14]. Secondary corrections in the area of the midface are generally more complex and more difficult to be performed and often they do not lead to satisfactory results.

Exceptions are not dislocated, not or minimally mobile Le Fort fractures without disturbed occlusion or with toothlessness. In this context, there is no indication for surgery. However, the compliance of the patient has to allow such a procedure [54].

### 4.5 Therapy

The basic principle of treatment consists of an accurate, anatomically correct reconstruction and securing of the bony structures in all three dimensions.

In cases of closed treatment of Le Fort fractures, the dislocated fragments are repositioned without exposing the fracture lines and after reaching the target position they are fixed in their position by mandibulo-maxillary fixation (MMF) and/or wires to further cranially located stable bones [17]. Today this technique of wire suspension is only rarely applied. The closed treatment by MMF and elastic tractions, however, is regularly applied in minimally dislocated and also after reposition of stable Le Fort fractures and in patients with reduced operability. The closed reposition can also be indicated in acute situations for reduction of bleedings and CSF leaks, especially in the context of severely dislocated Le Fort II and Le Fort III fractures [54].

Hereby it must be considered that the maxilla has the tendency to deviate in distal direction because of the traction of the pterygoid muscles. Even MMF cannot safely inhibit that the maxilla is later in a too distal position leading to class III malocclusion. The muscular forces can be strong enough to move the mandibular joints dorsally into the external articular canal. After releasing the MMF and sinking back of the head of the mandibular joint, the maxilla can be in a too distal position in relation to the mandible [54]. Hence it is important to mobilize the midfacial fragment in a way that the maxilla is passively positioned in the correct position. For this purpose special instruments such as Rowe’s forceps, Strohmeyer’s hook, and mobilization instruments according to Tessier are at disposition [54], [17].

In cases of Le Fort I fracture, generally an intraoral incision to expose the fracture line is applied and the maxilla is stabilized after reposition in correct occlusion by means of 4 miniplates in the area of the anterior midfacial pillars (lateral to the piriform aperture, on the zygomatico-alveolar crest). In case of larger bony defects after midfacial comminuted fractures, sometimes bone transplantats have to bridge the defects in the area of the trajectories. 

In Le Fort II fractures, the fracture lines are usually exposed in the area of the zygomatico-alveolar crest, in the area of the orbital rim, and if needed also in the area of the nasofrontal region and fixed by means of miniplates. Intraorally, the fracture area is reached via the classical sulcus incision in the maxillary vestibulum and the infraorbital edge is exposed via different transfacial or transconjunctival incisions. The nasofrontal region can be exposed by trauma given access, an incision in the area of the glabella, or the coronal incision. The process of surgical treatment of a dislocated Le Fort II fracture consists of the exposition of all relevant fracture areas, the mobilization and correct reposition of the fragments, stabilization of the correct occlusion via MMF, and application of osteosynthetic plates in the end. If necessary, relevant areas are augmented [14]. An alternative to cover the defects and to reconstruct comminuted zones consists of applying titanium meshes [13]. Finally the correct occlusion has to be verified [54].

In the context of surgical treatment of Le Fort III fractures, the facial skull in the nasofrontal and zygomatico-frontal area must be secured to stable bony structures of the neurocranium after reposition [17]. For the correct sagittal position of the midface the reconstruction of the zygomatic bone structures is of great significance. For exposition and osteosynthesis in these areas, a coronal access may be required.

### 4.6 Traumatic neuropathy of the cranial nerves (CN)

Lesions of the cerebral nerves are mostly associated with skull base injuries. In 50%, extracranial neurological complications are found [11]. Injuries of the skull rank third in the origins of uni- or bilateral anosmia [239]. If cerebral nerves are part of the injury, most frequently the 2^nd^ trigeminal branch and the olfactory nerve are concerned, followed by the facial nerve and the nerves of the eye muscles III, IV, and VI. Those nerves are more likely to recover than CN 1 [240]. In 12.8% of cranio-cerebral injuries, olfactory dysfunction must be expected, in rhino-basal fractures with CSF leak anosmia can be expected in 27.5% [205], [241]. Trauma to occiput leads more often to anosmia than frontal trauma [242]. The reason may be the direct lesion of the olfactory bulb or the rupture of olfactory fibers at the cribriform plate, bleeding into the olfactory fibers, or contusion of the olfactory bulb and tract [20]. Even a mild head trauma can lead to a temporarily impaired olfaction [243]. Early psychometric diagnostics should be performed in order to meet medico-legal questions by reliable findings after surgery.

In 50% of traumatic dysosmia, a complete bilateral anosmia is found. In 20% the anosmia is unilateral, in the other cases, hyposmia is diagnosed. The prognosis of hyposmia is good, the one of anosmia is poor [242]. Only 11.3% and 2.3% completely recover from anosmia and hyposmia, respectively. The origin of the lesion in this context is unimportant [244].

The oculo-motor nerve is injured either within the orbit or in the superior orbital fissure. Isolated lesions of CN III are very rare in cases of closed cranio-cerebral trauma. Nonetheless, also in mild closed head injury without concussion paresis of CN III may occur [245]. MRI can sometimes reveal an enhancement and swelling in the cisternal course of the nerve [246], [247], [248]. Clinical signs are ptosis, mydiasis, and impaired motility of the ocular bulb. The following developments possible: no improvement, regeneration, and aberrant regeneration that may lead to paradox eye movements. This is typically elevation of the eyelid when trying to look downwards, further the inadequate pupil motor activity can be triggered by change of the line of sight. Sebag describes a patient who was blind in the affected eye, had no pupil reaction on accommodation or consensual luminous stimulus, and when looking downwards, myosis was triggered [249]. Penetrating lesions may directly injure all nerves of the orbit and lead to an orbital apex syndrome [250].

Isolated lesions of the trochlear nerve are rare. They mostly occur in combination with lesions of the oculo-motor nerve. The location of injury is in the temporal bone and pterygoid process or endocranially at the tip of the petrous bone. The failure of the superior oblique muscle adducting the globe in caudal direction leads to vertical diplopia. Spontaneous remission 3–6 months after trauma is described [18], [251].

The 6^th^ cranial nerve can be injured in its long intracranial course by fractures of the skull base mostly in the area of the petrous bone [252], [253]. Furthermore, lesions caused by increased cerebral pressure are possible. Epidural hematoma for example may be the reason. Nayil describes a case where an epidural hematoma at the apex leads to bilateral paresis of the CN VI [254]. Fractures in the area of the lateral orbital wall may also lead to disturbed abduction. Clinically, the damage can easily be proven by abduction deficit. Indirectly, a traumatically induced carotid cavernous sinus fistula may be the origin [36].

Lesions of the optic nerve are important for rhinologists because the optic nerve in its extracranial course can be reached by endonasal surgery. The different partly controversially discussed aspects will be described in the following. The ophthalmologic and neurosurgical aspect of this topic will not be focused on and only be mentioned where it is necessary to reasonably explain the rhinologic field of activity.

In about 5% of closed head injuries, the optic nerve is damaged. The consequence may be a permanent loss of vision. Since the pathophysiology of the lesion of the optic nerve by blunt trauma is only incompletely understood, therapeutic recommendations are based on analogies from the field of traumatology [255].

In up to one fifth of severe fronto-basal lesions, traumatic lesions of the optic nerve are observed [256]. Midfacial fractures are associated with loss of vision in one eye in 1.2% of cases [257]. An acute visual loss is found in 1.7% of cranio-cerebral lesions with involvement of the eye [258]. In half of the cases, the visual loss persists [259].

#### 4.6.1 Anatomical particularities of the optic nerve

The optic nerve is a part of the diencephalon and belongs to the white matter of the brain. Therefore it is covered by three cerebral membranes that encompass it in its course with different thickness. The optic canal itself is lined with periost that passes on at the cerebral side to the dura mater, at the orbital side to the periorbit and the dura of the optic nerve. The pia mater firmly adheres to the nerve and accompanies it from the chiasm to the entrance into the bulb. The dura mater continues in the periostal lining. Also the arachnoid accompanies the nerve in its whole length. On the way through the canal, the membranes are closely connected to each other, the subarachnoid spaces containing fluid are very narrow. Consequently, osseous deformities of the pterygoid are transmitted directly to the nerve [260].

**Figure 10:**
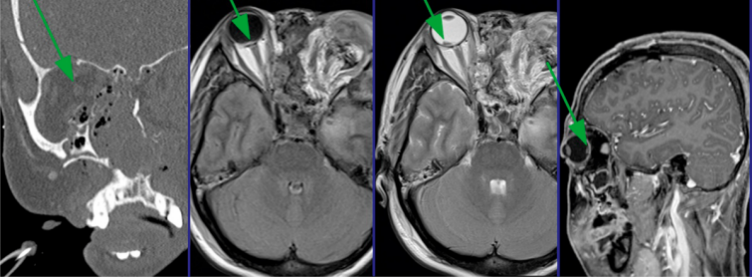
Avulsion injury. MRI: The arrows indicate bleeding into the location of rupture of the optic nerve (contralateral most severe contusion injury of the bulb).

For about 1 mm, the optic nerve has an intraglobal course. Only in cases of very intensive trauma, avulsion injuries occur. There is no therapy in those cases (Figure 10 (Fig. 10)).

More frequently, nutritive disturbances are found because of damaged ciliary arteries [258].

The intraorbital part measuring 25–30 mm, the nerve has an S-shaped course. There is some spare length that allows movement of the globe. Lesions in this area are caused either by piercing of bone fragments and foreign bodies or development of increased pressure intraorbitally in the sense of an orbital compartment syndrome.

The nerve is at highest risk in its 6–8 mm intracanalicular course. At the entrance into the canal, the nerve is surrounded by the anulus of Zinn (also known as annular tendon or common tendinous ring). In 40% the ophthalmic artery enters medial to the optic nerve into the dura canal, in 35% in the middle below the nerve, and in 25% lateral below the nerve, in 85–90% it runs inferolateral of the nerve through the bony canal, in the other 10–15% of the cases, however, it runs medial and is potentially at risk during decompression surgery. If it is transsected, vision is lost [261].

Regarding the surgical technique, the course of the optic nerve through the dorsal ethmoid bone and the sphenoid sinus is important. Only if a sufficient pneumatization exposes the nerve in the sphenoid sinus, an endonasal endoscopic intervention can be performed. Special attention must be paid to sphenoethmoid cells as in 10–15% of cases they expose the nerve [107]. 

#### 4.6.2 Epidemiology and etiology

The most frequent causes of traumatic lesion of the optic nerve are traffic accidents, to an increasing rate also bike accidents, but also violence and falls. In 50% of cases, initial unconsciousness is observed. If loss of vision occurs, it is mainly immediately together with the trauma [262]. A blunt concussion is transmitted from the cranial bone to the canal of the optic nerve. Direct intraorbital or intraocular lesions of the optic nerve are mostly a sequel of sharp injuries as for example cutting damage or gunshot wounds. Dissection of the optic nerve is irreversible and therapy is not possible. The difference must be made regarding indirect damage of the optic nerve in its orbital course caused by increased pressure in the orbital compartment. If the pressure within the orbit is greater than perfusion pressure especially of the ciliary arteries, an ischemic disturbance occurs that can be reversible if it is treated in time.

The classification of lesions according to their pathophysiology and injury patterns is based on Frank B Walsh. It provides an orientation of the therapeutic consequences [263] (Table 5 (Tab. 5)).

The traumatic optic neuropathy (TON) roughly follows this pathomechanism. The trauma causes an arterial compression or spasms leading to ischemia that results in an intraneural edema and necrosis of the optic nerve. So it is plausible to claim for decongesting and space-creating measures in order to prevent further damage [264]. 

Beside the indirect damage of the optic nerve in its canal by pressure, other trauma patterns of the nerve must be considered. An intraconal retroglobal hematoma may lead to ischemic damage of the nerve and thus to the loss of vision via the compression of the ciliary arteries surrounding the optic nerve. More frequently than traumatic neuropathy of the optic nerve, retroglobal hematomas and ruptures of the globe cause a loss of vision. The mechanism of the accident plays a more important role than the fracture pattern regarding the type and extent of the trauma [258]. 

Carta identified blood in the dorsal ethmoid cells, age greater than 40 years, and unconsciousness as prognostically relevant risk factors regarding vision [265]. Panja could reveal primarily the following factors as prognostically unfavorable: complete loss of vision, intervention later than 7 days after trauma, missing response in the visually evoked potentials, and radiological evidence of fracture fragments at the roof of the sphenoid sinus [266].

#### 4.6.3 Diagnosis

In order to diagnose afferent lesions, psycho-ophthalmological and neuro-ophthalmological procedures are at disposition that are highly reliable in cooperative patients. In case of severely injured patients, the reliability reduces to 30%. Gellrich and co-workers recommend a diagnostic algorithm that refers to the findings of visual evoked potentials (VEP) beside computed tomography [267].

Polytrauma patients generally are not able to undergo exact ophthalmological diagnostics. Two thirds of patients with TON are unconscious at the time of referral. Therefore psycho-physical diagnostic means are not appropriate. Onset of visual loss is hard to find out exactly though it is of paramount importance to estimate prognosis. Objective procedures that do not require the patient’s cooperation are not exact enough for the scientific analysis of therapeutic procedures in cases of TON.

The significance of electrophysiological diagnostics (visually evoked potentials and electroretinography) [268] as basis of surgical indication is discussed controversially even if flash VEP can be applied in patients that are not able to cooperate [269], [270].

The only prognostic criterion that receives broad consensus in the literature is the initial vision at the time of trauma.

An ophthalmologist should always be involved in the diagnostic procedures.

A possible order of clinical diagnostics may be the procedure suggested by Luxenberger [260]:

- Computed tomography, thin-layer of 1 mm, multiplanar reconstruction in the context of cranio-cerebral trauma diagnostics.
- Measurement of the ocular pressure.
- Swinging flashlight to identify a relative afferent pupillary deficit (RAPD).
- Ophthalmometry (Hertel exophthalmometer) to detect a unilateral bulbar protrusion.
- Fundoscopy: typically, the pupils and the retina are inconspicuous during the first weeks after trauma. The atrophy of the optic nerve only follows after 3–4 weeks. Circulatory disorder, however, may become apparent earlier.
- Recording of visual acuity, color vision (red is the first quality to fail), and perimetry if the patient is able to cooperate.
- Flashlight VEP (visually evoked potentials) in unconscious patients.

The best functional test in the clinic is visual control and perimetry. Whenever possible, it should be repeated in intervals and be documented exactly. The mobility of the globe and reaction tests of the pupils are also obligatory parts of ophthalmological diagnostics.

With procedures described here, mostly no sufficiently safe statement about the condition of the optic nerve can be made in polytrauma patients. In order to find an indication for such far-reaching measures such as pharmacotherapy with high-dose steroids or surgery at the tip of the orbit, at least for medico-legal reasons a possibly reliable finding of the optic nerve function is required. The recording of visually evoked potentials (VEP) can also be performed in unconscious patients and with shut eyelids so that hints on the function of the optic nerve can be gained [271], [272]. However, there are serious limitations of the procedure [268], [269], [270], [273]. Regarding the two procedures of standard diagnostics, pattern VEP must be excluded because it requires the patient’s cooperation.

**Figure 11:**
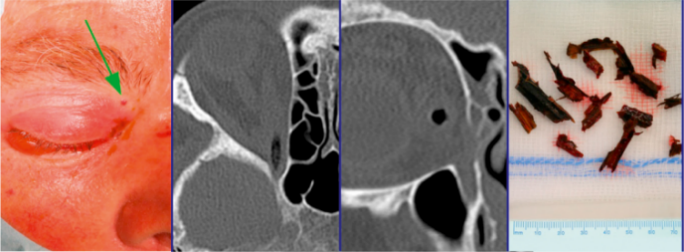
Foreign body of the orbit. In the CT scan the biological material is detected as gap. The foreign body caused a minimal wound when penetrating (arrow in the left image). It was stopped in the orbital tip. Removal via an endonasal approach.

Direct lesions, caused by penetrating foreign bodies, canal fractures, hematoma, or compression of dislocated bone fragments can be assessed by CT diagnostics [274]. MRI findings can be helpful for diagnosis of avulsion injuries and hematomas of the optic nerve sheath, axonal injury, and ischemia. They are obligatory for fractures concerning the canal of the optic nerve [30] (Figure 11 (Fig. 11)).

MRI must only be performed if metallic foreign bodies have been excluded because they might cause additional damage of the orbit because of the strong magnetic field.

CT diagnostic cannot detect fractures with sufficient reliability unless they are dislocated. The elasticity of the bone can lead to a temporary compression and immediate recovery of the tissue. So the anatomical findings at the time of CT scan do not necessarily depict the full extent of bony lesions. Hematomas of optic nerve sheaths or swellings cannot be reliably detected or excluded by MRI. An uncertainty remains in the diagnostic algorithm that must be taken into consideration when interpreting studies. CT diagnostic, however, is a relevant part in the context of surgical planning, for the reason alone to define the transnasal approach to the optic nerve.

#### 4.6.4 Indication, time of intervention

In literature, mainly three therapeutic approaches or combinations of these respectively are discussed. Corticosteroids in various dosage, surgical decompression of the nerve in its canal, and spontaneous improvement of the situation. 

There is no sufficient evidence for any of the said therapeutic regimes to be superior to others. For methodological reasons there is no change in sight [275]. Main issues in the context of a treatment concept is to define a time interval in which therapy, especially surgery, should be recommended, wether or not application of corticosteroids is helpful or even counterproductive, and if decompression of the bony canal alone is sufficient – if a benefit can be expected at all – or if the optic nerve sheath should be incised as well [276], [277].

It is general consensus that in cases of orbital compartment syndrome the indication for surgical decompression is to be made generously. Bleedings and edematous swellings within the orbit delineated by the orbital septum in anterior direction can lead to a limited vision of red or an impaired visual field and to reduced vision. In those cases, immediate intervention is required. The first measure is the lateral canthotomy and cantholysis. In cases of insufficient pressure relief, additional measures must be taken [234], [235]. As emergency procedure still in the emergency room, lateral canthotomy with cantholysis can also be performed under local anesthesia. Drug therapy must not delay surgical relief [278], [279]. Lower eyelid release indicates the correct performance of the intervention. In the further course, the endonasal technique of orbital decompression represents the gold standard and is a routinely performed procedure for experienced sinus surgeons [278], [280], [281], [282], [283]. By enlarging the volume of the orbit, on the one hand further pressure relief is achieved, on the other hand the traction of the optic nerve is reduced when cantholysis led to proptosis. The optic axis changes as an effect of this procedure and postoperative strabismus results. 

The prognosis of vision mainly depends on the dynamics of visual acuity after the trauma [262]. If it is possible to assess the dynamism of the injury, the indication should be made as follows:

In case of immediate loss of vision (i.e. concussion of the optic nerve, primary pressure necrosis, laceration of the nerve or chiasm), surgery should not be performed. Concussion of the optic nerve does not require surgery because it may recover spontaneously, in case of the other said pathologies, vision is lost. The situation is quite different when the loss of vision is delayed: if the swelling of the nerve leads to hypoxemia, an intervention seems to be promising [263].

Secondary blindness, intracanalicular lesion, dislocated bone fragments, increased pressure in the orbit (orbital compartment syndrome) should call for surgical measures.

Some authors claim that intervention should be performed within 8 hours to be successful [284]. On the other hand numerous cases are reported that recovery of vision is found even after longer intervals [283].

#### 4.6.5 Conservative therapy

Some authors decline any specific therapy but await the spontaneous course of the disease. 20–60% of the patients experience an improvement of their vision [285], [286]. Since it was not possible to find an evidence for the effectiveness or superiority of the treatment with steroids or surgery compared to wait-and-see, the risks associated with therapy should be avoided [287].

In order to create a reasonable basis for therapeutic decisions regarding vitally threatened vision, clear and understandable guidelines are needed. In a meta-analysis of the literature available up to 1996, Cook and co-workers drew the conclusion that therapy with steroids and surgery are superior to wait-and-see. However, they admitted that methodical problems do not allow a final statement. They mentioned further an international study by the same authors on the topic that was not been finished at that time [285]. With regard to this study, a bias of time between trauma and inclusion into this study must be taken into account [284] (Table 2 (Tab. 2)). Later publications do not see an advantage of steroid therapy, they rather mention the risks and recommend wait-and-see.

Corticosteroids are applied since 40 years in traumatic lesions of the optic nerve [287]. The results of lesions of the central nervous systems in animal trials were transferred to the optic nerve in humans as the ON is part of the brain as well [288]. A neuro-protective effect was expected because of the antioxidative effect and an inhibition of lipidperoxidation induced by free radicals. Due to the low pharmacological correlations and the effective inhibition of the inflammatory cascade, methylprednisolone is preferred to other representatives of corticosteroids. 

The clinical recommendation of steroid therapy is based generally on the National Acute Spinal Cord Injury Study (NASCIS II and III) [276], [286], [289]. The recommendation is given in this study that therapy should be initiated within 8 hours. The results and especially their transferability to TON, however, are still and persistently discussed controversially. A significant difference in outcome of the motor fibres was found, however, not of the sensory fibers. Furthermore, in the NASCIS study methodical ambiguities were seen (randomization, post-hoc analysis of a small subgroup for the actual statement on early treatment of spinal cord injuries) that limit the value [276], [290], [291], [292].

In a randomized, double-blind, and prospective study, Entezari and co-workers evaluated the effect of high-dose steroid therapy (250 mg prednisolone i.v. every 6 hours for 3 days, afterwards 1 mg/kg body weight of prednisolone for 14 days) compared to placebo in 31 patients with unilateral indirect TON. All patients were included into the study within 7 days after trauma. As explained, this is may pose a typical problem of studies on this topic: the time between injury and onset of therapy amounted to 6 and 168 hours. The effect of spontaneous remission could be encompassed in different measures into the one or other group. Furthermore, patients with missing light reaction were included in the same way as patients with mostly intact vision. Regardless those methodical pitfalls, the authors could not reveal a significant difference between in the therapy groups [293].

A double-blind, randomized, and placebo-controlled study on the effectiveness of mega dose therapy with methylprednisolone of cranio-cerebral trauma patients regarding their survival (Corticosteroid Randomization After Significant Head Injury, CRASH-study) should include 20,000 patients originally. After 10,008 patients had been included the ethical committee stopped the study because it became obvious that the survival in a 6-months follow-up was worse in the verum than in the placebo group. In the analysis performed by Roberts and co-workers, a higher mortality within 2 weeks after the accident was found in the group treated with methylprednisolone [294]. These results have to be taken into consideration for the frequently observed patients with cranio-cerebral injuries with TON [295].

In a review of the Cochrane Collaboration, Yu-Wai-Man and Griffiths draw the conclusion that no convincing data are present showing a superiority of steroid therapy in comparison to mere observation of the clinical course. Reviewing 247 publications, however, none was found that would have met the formal criteria demanded by the Cochrane Society. Nonetheless, some studies and case reports were discussed, among others the paper of Cook and co-workers that was already cited here. All those investigations seem to be methodically limited. For the situation of spinal cord lesions, Bracken wrote a new Cochrane review in 2012. He concludes that high-dose methylprednisolone is the only treatment option that has an impact on the remission of, however, only motor neurons [296].

Especially in intensive care patients, the side effects of high-dose or mega-dose methylprednisolone therapy must be considered [255], [297]. Beside gastro-intestinal complications, diabetogenic effect and steroid psychosis, in particular the immuno-suppressive effect with the risk of nosocomial infections must be taken seriously [298].

The case wether or not therapy with growth factors is helpful, cannot be determined at present. Animal experiments seem to indicate this direction [299], [300]. First clinical reports show positive effects, but they are characterized by the same methodical problems as other studies on said therapeutic procedures [301], [302].

#### 4.6.6 Endonasal, transorbital, or neurosurgical pterional decompression

In the thirties of the last century, Sewall, Dandy, and Pringle were the first to describe decompression of the optic nerve. During the world wars, the fronto-temporal approach became standard, only afterwards, the trans-temporal, transorbital, transfronto-intra- and extradural, transnasal, and transethmo-sphenoid approaches were developed [274], [284], [303].

The transethmoid access has the advantage of rapid wound healing compared to the transcranial access. The olfactory bulb is protected, the surgical stress is lower which is very important in polytrauma patients requiring intensive care, and no visible scars result [304], [305]. If the optic nerve can be sufficiently exposed in the ethmoid and sphenoid sinus, if no contraindications exist, and if no neurosurgical procedure actually requires the transcranial approach anyway, the endoscopic transnasal-transethmoid access is widely accepted [306]. In the same way, an orbital compartment syndrome can be relieved [260]. The surgical decompression of the nerve also provides a chance of improved vision as salvage measure if conservative therapy failed [286], [307].

Performed by an experienced surgeon, the intervention can be judged as being safe and low in risk. Beside the typical risks of endonasal sinus surgery, the patient has to be informed about a possible lesion of the ophthalmic artery, parts of the nerve by slitting the sheaths, CSF leak, and meningitis. Different authors avoid the transection of the annulus of Zinn and the slitting of the sheath of the optic nerve in order not to run the risk of causing CSF leak or damaging neural fibers iatrogenically [277], [307], [308]. If a sufficient decompression of the nerve can be achieved without opening the sheath of the optic nerve in case of present edema or hematoma, must be questioned [309].

If the course of the optic nerve is beyond the sphenoid sinus or if the roof of its canal is fractured, the neurosurgical temporal approach is chosen. After detaching the temporalis muscle, a circumscribed trephination is performed (pterional). After lifting of the dura, the nerve can be exposed in its whole extraorbital course. Even this access is characterized by a relative low trauma and rapid recovery of the patient. Mastication may be impaired [310].

The transorbital approach had been described even before the transethmoid access but it is no longer widespread. Current publications on transorbital surgery of the anterior skull base could establish a new approach to the tip of the orbit [311].

In the Cochrane review on the topic of surgical therapy of TON, the author concludes, in analogy to the investigation of steroid therapy, that there is no convincing evidence in literature on the effectiveness of decompression surgery [276]. In contrast to that, case reports give evidence that the decompression of the optic nerve led to recovery of vision in several cases [275].

#### 4.6.7 Outcome, case reports

The available data result in one simple statement: there are no evidence-based significant differences between the therapeutic options [262]. Thus on the basis of the current medical research no clear therapeutic recommendation can be given. Methodology as defined by means of evidence based medicine seems to fail in terms of acquisition of new insights to the topic [276].

The most important paper on traumatic optic neuropathy (Levin LA: The treatment of traumatic optic neuropathy: The International Optic Nerve Trauma Study) had been planned as international randomized 2-arm therapy study. Two years after its beginning, the project had to be abandoned because there were not enough patients who could be included based on the required criteria. It was continued as observational study and provides the most systematical results. The most important ones are:

- The initial vision is a strong predictor for outcome. This statement is confirmed by several other authors [257], [267], [286], [312].
- There is no proven advantage of the application of mega-dose therapy with methylprednisolone equivalents of more than 5,400 mg in comparison to low doses.
- Onset of therapy is not of upmost relevance.
- The findings of CT diagnostic do not allow any conclusion on vision acuity on the long run.
- Neither steroids nor decompression surgery is better than wait-and-see in an unselected patient group.

In the data analysis, some limitations of the results are described. So in the group of operated patients a higher number of severe cases were found. Steroid therapy was started earlier than taking a decision for surgery. A serious bias is given as only patients underwent surgery who failed conservative therapy (wait-and-see or steroids). Therefore patients with poor prognosis were selected for the surgery. Possibly canal fractures that more frequently lead to surgical indication are another bias because they seem to be associated with a poorer outcome than cases without fracture.

Based on the present case reports, it is not possible to define clear inclusion criteria, the preconditions of therapy are rarely comparable, and also the endpoints of the studies are regularly not well defined. The most difficult aspect of any study design is to assess the initial vision of the patient immediately after trauma as the most important prognostic criterion. All authors agree that the inclusion of patients with unclear vision leads to a bias of the results, independent from therapy [262], [283], [313]. Another important aspect is the beginning of therapy. The earlier therapy starts, either conservative or surgical, the higher is the chance to address patients, who would have experienced spontaneous remission anyway. 

Animal experimental studies on rats with standardized trauma of the optic nerve are only transferable to humans in limited extent. However, they help understanding general mechanisms of pathophysiology. In one publication [314] no difference was found between the various prednisolone regimes and controls regarding the axon loss. Another paper revealed a significant dose-related decrease of the number of axons with increasing steroid dose [295].

#### 4.6.8 Summary

High-dose and mega-dose therapy should be abandoned in the treatment of TON. If lesser dosages have a positive effect, is unclear. The results are inconsistent [255]. In an animal experiment, a neurotoxic effect of corticosteroids was revealed in TON.

There are patients who benefit from surgical decompression. In this context it is unclear how to select them prior to surgery. A possible group would be those patients with hematoma within the optic nerve sheath and reduced vision. Anyhow, the surgical decompression is only recommended in patients with delayed visual loss or in those who do not experience improvement of vision after 4 days. A precondition in any case is a remaining visual acuity. General recommendations for surgery do not exist. (Tab. 6)

## 5 Lateral midface

### 5.1 Fractures of the zygomatic bone

#### 5.1.1 Epidemiology, pathogenesis, classification

Fractures of the zygomatic bone complex are rather frequent because of the prominence of the zygomatic bone [111]. Beside isolated fractures of the zygomatic arch, they belong to the typical lateral fractures of the midface resulting from direct trauma to the zygoma. Among the most important origins of trauma, violence and traffic or sports accidents must be mentioned [38]. Depending on the direction of the impact vector, rotation and/or caudal and dorsal dislocation of the zygomatic bone results [18]. The dislocation of the zygoma occurs in direction of the impact and then secondarily due to traction of the masseter muscle [17]. In cases of isolated zygomatic bone fractures, the fracture line extends through the zygomatico-frontal suture along the lateral orbital rim and from there in caudal direction along the lateral and anterior orbital floor to the infraorbital edge. From the infraorbital edge the fracture line passed through the anterior wall of the maxillary sinus and in general through the infraorbital foramen to the zygomatico-alveolar crest. From there, the fracture line reaches the inferior orbital fissure cranial to the lateral and posterior wall of the maxillary sinus. The greater wing of sphenoid at the zygomatico-sphenoid suture is involved as well [13] as the zygomatic arch. The orbital floor is nearly always affected in cases of fracture of the zygomatic bone. After an impact of high violence, also a comminuted fracture of the zygomatic complex may result and parts of the zygomatic bone may dislocate into the maxillary sinus. Concomitant injuries of the zygomatic complex often affect the ocular globe and optic nerve [315].

In literature, several classifications for fractures of the zygomatic bone complex are found that could not be established for regular clinical application [14], [20], [316]. Differentiation of dislocated and not dislocated fractures seem clinically useful [54].

#### 5.1.2 Clinical symptoms

In order to correctly diagnose a fracture of the zygomatic complex, a careful examination of the patient has to be performed together with an assessment of history of the accident [54]. Possible clinical symptoms of zygomatic bone fractures are listed in the following table (Table 6 (Tab. 6)). The symptoms depend largely on the pattern and the extent of the fracture of the zygomatic complex [233].

The patient should be informed as soon as possible that he must not blow his nose because it may lead to emphysema due to laceration of the mucosa of the maxillary sinus and increased pressure in the paranasal sinuses [134]. The emphysema can extend via the neck into the mediastinum [185], [215], [317]. The emphysema is clinically relevant especially because of the massive swelling of the soft tissue that makes initial examination of the eye difficult and leads to delayed surgery. Usually it is completely absorbed during the first postoperative days though.

It may be of highest clinical relevance to early detect neurological and ophthalmological complications that require immediate surgical intervention [233]. The zygomatic complex is involved in the bony structures of the orbital floor, the infraorbital rim, and the lateral orbital funnel. Hence, each zygomatic bone fracture may damage the eye. So it is essential to perform primary careful examination of vision [154], [230], [318]. The spectrum of lesions of the eyes reaches from injury of the globe and the eye muscles to lesions of the optic nerve with associated blindness [101], [145].

#### 5.1.3 Radiological diagnostics

Conventional imaging of the paranasal sinuses and axial imaging of the used to be applied for radiological assessment of zygomatic bone fractures [156]. In intubated and polytrauma patients, however, this kind of radiological imaging cannot be performed. It is of little value, particularly the orbital floor is not represented well enough. This is why conventional imaging has lost its importance during the last years. It is only applied for overviews after zygomatic bone fractures or for controls after osteosynthesis and removal of materials [156]. As radiological standard procedure CT scan is meanwhile established for diagnostics of the midface and also fractures of the zygomatic bone complex [156], [233], [319]. With a CT examination, bony structures as well as other sequels of injury such as foreign body, hematoma, herniation, and emphysema can be well depicted. Injuries of the zygomatic complex can be assessed in axial, coronal, and sagittal levels. For very complex trauma, also a 3-dimensional reconstruction on the basis of DICOM datasets can be performed and thus facilitate the evaluation of the injuries. If the CT datasets are implemented in surgical planning software, navigated surgical control or the creation of individual grafts for reconstruction of the orbital walls can be performed [13], [24], [75].

Modern software allows the virtual planning of surgery with segmentation of the affected regions, mirroring of skeletal parts in comparison to intact bony structures as well as fusion of different data sources beside the 3-dimensional presentation of the current situation [13]. Generally, also cone beam tomography (CBT) may be appropriate for the 3-dimensional presentation of zygomatic bone fractures. The disadvantage of this procedure is the low significance regarding soft tissue structures. In this regard, CT diagnostic is superior. Also the globe as well as other relevant structures of the orbit (e.g. eye muscles, optic nerve, retroglobal hematoma) can be well assessed with computed tomography [156]. In case of orbital involvement with disturbed eye movement, en- or exophthalmos, reduced vision as well as retrobulbar pains, CT scan is indicated [156].

#### 5.1.4 Surgical indications

In case of non-dislocated zygomatic bone fractures without functional disorders, there is generally no need for surgical therapy. Functional disturbances in the context of fractures of the zygomatic complex concern the eye, the infraorbital nerve, and the opening of the mouth [233]. Frequently, fractures of the zygomatic complex lead to impairment of the infraorbital nerve which can be temporary or also permanent. The nerve can be damaged directly because of the trauma or by pinching in its bony canal when the fracture passes through the infraorbital foramen and if fragments are dislocated. Sensitivity disorders in the area of the infraorbital nerve itself do not indicate surgery. In case of non-dislocated fractures of the zygomatic bone after direct violent trauma, there is explicitly no indication for surgery because the exposition of the fracture could lead to further damage of the nerve [105], [106]. In case of suspected pinching of the infraorbital nerve caused by dislocated bone fragments, there is an indication for surgery, however, also closed reposition of the zygomatic bone complex may be possible [18], [54].

The involvement of the zygomatic arch and according dislocation in medial direction can lead to an impairment of the coronoid process of the mandible and thus to impaired opening of the mouth. In those cases, a clear indication for open or closed reposition of the fractures is given [233].

#### 5.1.5 Therapy 

**Figure 12:**
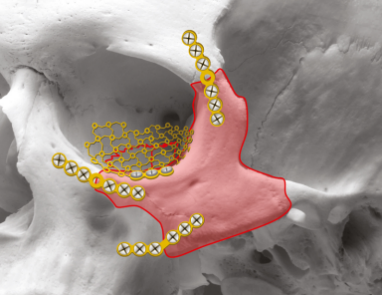
Tripoid fixation of a zygomatic bone fracture in combination with a titanium mesh for stabilization of the fracture of the orbital floor

The extent of damaged bones and the degree of dislocation determine where the fracture is exposed. By means of osteosynthesis the repositioned fragments are stabilized in order to permanently achieve an exact anatomically correct position of the zygomatic bone [13]. Generally, the objective is to perform reposition and osteosynthesis in the simplest way via limited transfacial or transconjunctival approaches [13]. For osteosynthesis, usually mini- and microplate systems are applied that are positioned over the fracture lines and tightened by screws. They are sufficiently robust to keep the fragments in the correct position. Often, a so-called 3-point fixation (“tripoid”) of the zygomatic bone over the zygomatico-frontal suture, the infraorbital rim and the zygomatico-alveolar crest is performed [13] (Figure 12 (Fig. 12)). After reposition and stabilization of the body of the zygoma accompanying fractures of the orbital floor or orbital wall are addressed (see also chapter on fractures of the orbital floor).

Another order of the surgical procedure can be necessary in emergency situations when loss of vision is immanent, in case of retroglobal hematoma or in infantile trap-door fractures with impaired eye movement [142], [156], [320].

For therapy of fractures in the area of the zygomatic complex, numerous procedures are at disposition that will be discussed in the following.

##### 5.1.5.1 Conservative treatment

In cases of non-dislocated or minimally dislocated fractures of the zygomatic bone, the indication for conservative procedure is made. Those are physical measures (cooling ect.) and drug therapy (analgesics, decongestion). Furthermore the patient is instructed to eat soft meals only to avoid secondary dislocation of the zygoma as a result of traction of the masseter muscle. In patients with severely impaired operability, conservative treatment may be indicated despite dislocation of the zygomatic bone [120]. On the other hand, orbital complications even without dislocation of the zygomatic bone complex can be an indication for emergency surgery.

##### 5.1.5.2 Surgical therapy

Surgical therapy is generally applied in all dislocated, instable, and comminuted fractures of the zygomatic bone. For closed reposition, the zygomatic bone is repositioned without exposure of the fracture gaps via a bone hook that is inserted percutaneously below the zygomatic bone. It is expected that the reposition remains stable even without osteosynthesis [205]. In cases of open reposition, the repositioned fragments are stably fixed with up to date osteosynthetic plates and screws in the anatomically correct position.

##### 5.1.5.3 Closed reposition

The exact reposition of the body of the zygomatic bone is the decisive step of fracture treatment in every dislocated fracture of the zygoma. In some cases the achieved result remains stable also without osteosynthesis so that exposition of the fracture lines can be sidestepped. Especially suitable are fractures where the zygomatic bone is broken as complete fragment and dislocated. Furthermore the fracture should be repositioned as early as possible. The two biggest problems are the missing control of fracture gaps and thus of the outcome of repositioning and the uncertainty of the postoperative stability of the repositioned zygomatic body [54]. Contraindications for closed reposition are complex or comminuted fractures of the zygomatic complex as well as doubts of the surgeon regarding the stability of the reposition. Furthermore, closed treatment of zygomatic fractures is automatically contraindicated if surgical revision of the orbital floor is necessary [54]. Advantages of closed reposition are: Minimally surgical effort, effective reconstruction of the zygomatic position and decompression of the infraorbital nerve. Sometimes not even general anesthesia is necessary for closed reposition of the zygomatic bone.

For closed reposition of the zygomatic bone, 2 transcutaneous approaches are recommended. The approach according to Gillies consists of reaching the zygomatic bone via a small temporal incision performed with the elevator [233]. It is important to incise the fascia of the temporalis muscle in order to insert the elevator directly below the zygomatic arch and bone. The second possibility is performed directly percutaneously with a bone hook. After stitch incision (about 2 cm lateral and caudal of the temporal canthus) the hook is inserted directly through the skin under the zygomatic bone and the zygomatic complex is repositioned [30]. The course of the zygomatic arch may be palpated with the hook after reposition to control the result. A less established technique of closed zygomatic reposition is the direct percutaneous insertion of the osteosynthetic screw or a “Caroll-Girard” screw into the zygomatic bone in order to pull the zygomatic bone out of the dislocated position and to reposition it [54]. A disadvantage of this last-mentioned method is the risk of damaged facial nerve [233].

##### 5.1.5.4 Open reposition without reconstruction of the orbital floor

The open reposition of the zygomatic complex is indicated when a comminuted fracture is found, or when the fracture is instable. This may be the case if initial reposition was performed with the bone hook, but then after release of the hook the zygomatic bone sinks back into the dislocated position.

The objective of open reposition is the control of fracture gaps in first place and thus the result. Second is reposition and stable osteosynthesis in order to secure the result. In case of open repositioning, a decision needs to be taken where to expose the fracture. In future, the control of fracture repositioning may be facilitated by use of 3D image converter technique in more places [13].

Location and number of exposed fracture sites depends on the experience of the surgeon amongst others [54]. On the other hand, the more complex a fracture is, the more regions have to be exposed and treated with osteosynthesis [233]. The complexity of a fracture does not only depend on the fracture morphology and the course of the fracture lines but also from the surgical access, the sight and accessibility of the fractured region and the stability of the fragments. Furthermore, the biomechanical needs of the individual fracture determine the number, position, and thickness of the osteosynthetic plates [54].

A reliable approach to adequate repositioning of a dislocated zygomatic bone is found at the transition zone of the greater wing of sphenoid to the zygomatic bone in the area of the lateral wall of the orbit [20], [318], [321]. Further areas of control for reposition of the zygomatic bone are the zygomatico-alveolar crest and the infraorbital rim. In contrast, the area of the zygomatico-frontal suture is considered as not being reliable for the visual evaluation of an exact repositioning of the zygomatic bone [20], [233], [318]. Repositioning of a fractured zygomatic arch in the context of zygomatic fractures does normally not require exposition under direct visual control.

Osteosynthesis of zygomatic bone fractures is classified in 1-point, 2-point, 3-point, and 4-point fixation. A detailed description of the procedure is provided in the “AO Surgery Reference” guide of the AO foundation [54]: 

###### c>1-point exposition and fixation

Not comminuted, simple fractures of the zygomatic complex may be sufficiently fixed with only one miniplate as long as repositioning is easy. The preferred access is via an intraoral approach with good overview and control of the zygomatico-alveolar crest and the infraorbital rim. Indications for this approach are missing separation of the zygomatico-frontal suture and intact orbital floor. The 1-point fixation is controversially discussed in the area of the zygomatico-frontal suture. From a biomechanical point of view, a fixation seems to be reasonable because it gives resistance to the traction forces of the masseter muscle. On the other hand, the internal aspect of the lateral orbit (zygomatico-sphenoid suture) must be exposed for good control of the result. 

###### 2-point exposition and fixation

Beside visualization of the zygomatico-alveolar crest, the exposition of the lateral orbital rim together with the zygomatico-sphenoid suture is useful. The exposition of those two points allows a good control of the correct 3-dimensional reposition of the zygomatic bone as well as a stable osteosynthetic fixation [233]. Alternatively to the lateral orbital rim, the infraorbital rim can be exposed as second point and stabilized by means of a plate. 

###### 3-point exposition and fixation

For 3-point fixation, the lateral orbital rim, the infraorbital edge, and the zygomatico-alveolar crest are exposed and stabilized by means of osteosynthesis. The exposition of 3 points allows a reliable control of the 3-dimensional position of the zygomatic bone and at the same time a stable fixation of osteosynthesis of all 3 fractured areas [321]. Besides a good control of the zygomatic bone, treatment of a fracture of the orbital floor may be an indication of 3-point exposition (Figure 12 (Fig. 12)). 

###### 4-point exposition and fixation

In the context of 4-point exposition and fixation, the zygomatic arch is exposed in addition to the three above-mentioned areas and if necessary it is stabilized with osteosynthesis. This procedure is indicated only rarely in cases of isolated lateral fractures of the midface and is rather applied in pan-facial fractures with comminution of the zygomatic bone and arch and loss of facial projection. Generally a coronal access (coronal incision) with its advantages and disadvantages is necessary for exposition of the zygomatic arch.

##### 5.1.5.5 Open reposition with reconstruction of the orbital floor

In many fractures of the zygomatic bone complex, the orbital floor is affected as well and requires surgical revision and reconstruction. Reconstruction of the orbital floor is always indicated if imaging reveals a large defect or herniation of the periorbital fatty tissue into the maxillary sinus. Additional reasons are a severe fragmentation and dislocation of the orbital floor, sometimes with impairment or pinching of the eye muscles.

Reconstruction of the orbital floor can explicitly not be indicated if the eye is severely damaged by trauma or previous surgery and if any additional manipulation in the area of the orbit increases the risk for the affected eye.

In cases of open reposition and reconstruction of the orbital floor, at least 2-point exposition and fixation is applied. For complex fractures with significant dislocation and simultaneous fragmentation, the 3 point exposition and fixation is the general rule.

##### 5.1.5.6 Surgical approaches for open treatment of fractures of the zygomatic complex

###### Approaches via traumatic skin lesions

Trauma in the area of the midface is often associated with soft tissue lesions. These can be used as access for fracture repositioning and osteosynthesis if they are located and sized appropriately. If their position is favorable but too small for exposition of the fracture, they may be extended via incisions parallel to the natural skin lines [54]. 

###### Transoral approaches to the zygomatic bone

In many cases the caudal zygomatic bone pillar with the zygomatico-alveolar crest is the region of first choice for exposition and osteosynthesis of a fracture [127], [150], [318]. After repositioning of the fracture, mostly a 2.0 mm L miniplate is sufficient to stabilize the zygomatic bone [233].

The transoral exposition of the zygomatic bone is either performed via a vestibular approach in the area of the maxilla or via midfacial degloving. The latter approach can be used for exposition of the whole maxilla as well as the nose and ethmoid region but is only rarely applied for treatment of midfacial fractures [54].

By a vestibular approach the maxilla can be exposed from the piriform aperture to the region behind the zygomatico-alveolar crest and cranially to the infraorbital rim [54]. For a better exposition of the zygomatic prominence, the primary horizontal incision can pass at the dorsal edge in cranial direction [54]. Especially in cases of bilateral exposition of the maxilla, sutures must counteract the contraction of the nasolabial muscles that had been detached from the periost. For this purpose, sutures are used that fix the bases of the nasal wings and join them medially (so-called cinching suture) and connect the mucosal edges in the midline in V-Y technique. A very good description of the surgical procedure for transoral approaches is provided by the AO Surgery Reference [54]. 

###### Transcutaneous approaches to the supero-lateral orbital rim

Osteosynthesis of the zygomatic complex in the area of the supero-lateral orbital rim is traditionally considered to be one of the main fixed points of a fractured zygomatic bone. Even for biomechanical reasons, a miniplate fixed there can optimally react on the muscular traction of the masseter muscle and thus avoid postoperative dislocation of the zygomatic bone [20], [30], [233]. Possible approaches are the lateral access at the eyebrow and the access through the upper eyelid (superior blepharoplasty approach).

The lateral access through the eyebrow is simple and can be rapidly performed. It allows direct sight to the zygomatico-frontal suture. The view on the inside of the lateral orbital wall, however, is very limited. Therefore control of the correct repositioning of the zygomatic bone at the transition to the sphenoid (zygomatico-sphenoid suture) is difficult [54]. In contrast, the superior blepharoplasty approach provides a significantly better overview of the whole lateral orbit and at the same time the scars remain minimal [54], [113]. A very detailed description of the surgical procedure for both approaches is found in the AO Surgery Reference [54]. 

###### Accesses to the infraorbital rim

The infraorbital rim is considered as an uncomfortable area for osteosynthetic fixation of an instable fracture of the zygomatic complex [164], [233]. Nonetheless, exposition of the fracture in this area is often necessary to reestablish the contour of the infraorbital rim. Generally transcutaneous or transconjunctival approaches are possible. The spectrum and the according advantages and disadvantages are described in the following. 

###### Transcutaneous access through the lower eyelid

**Figure 13:**
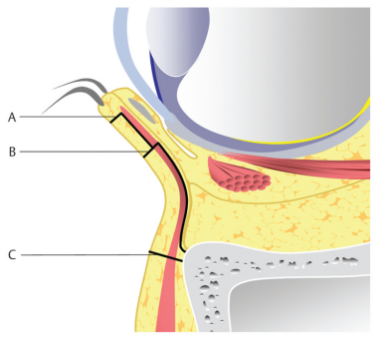
Transcutaneous approaches by the lower eyelid (A: subciliary incisin; B: incision in the middle of the lower eyelid; C: infraorbital incision)

In this context, subciliary incisions (inferior blepharoplasty incision), subtarsal incisions, and infraorbital incisions are classified [54], [124], [322] (Figure 13 (Fig. 13)). The subciliary incision is placed about 2 mm below the grey line of the lower lid. The subtarsal incision is performed in a natural skin line of the lower eyelid in the middle of the lower eyelid below the tarsus. In both incision, primarily only the skin is incised and the fibers of the orbicularis oculi muscle are not touched. The transection of the muscle is performed generally 3–5 mm below the skin incision. For subtarsal incision, the muscle can be transected at the same level or also some millimeters further caudal. The advantage of transection of the orbicularis oculi muscle on the same level is the better blood supply of the caudal wound edge because muscle and skin are not separated. The infraorbital approach directly over the infraorbital rim is generally considered as being critical [233]. The possible adverse effects are postoperative edema in the area of the lower eyelid and the unfavorable scar formation [54], [233]. The AO Surgery Reference for that reason does not specify the surgical procedure of the infraorbital approach. Generally transcutaneous approaches through the lower eyelid lead to scarring even if subciliary and subtarsal incisions are not eye-catching. Another disadvantage of transcutaneous approaches through the lower eyelid is the risk of lower eyelid ectropion [318], [322]. 

###### Transconjunctival approaches

**Figure 14:**
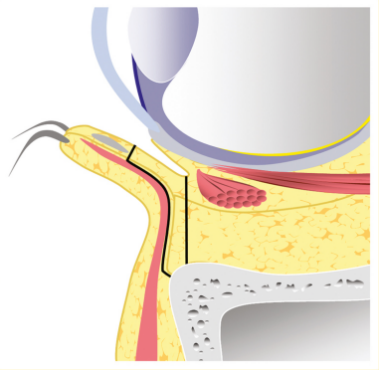
Classical transconjunctival approaches with pre- and postseptal dissection

The transconjunctival approaches were developed to avoid visible scars. Currently they are described as approaches of first choice in the literature for many cases of necessary exposition of the infraorbital rim or the orbital floor [233]. The difference is made between the classical transconjunctival approach (inferior fornix), the transcaruncular approach (medial transconjunctival), the transconjunctival approach with lateral skin extension and lateral canthotomy (swinging eyelid), the combination of transconjunctival and transcaruncular or the combination of transcaruncular and transconjunctival approach with lateral canthotomy (C-shaped incision) [54]. Beside the invisible scars, the advantage of these procedures is the possibility to extend and to combine the approaches. In terms of the classical transconjunctival preparation, the difference between pre- and postseptal preparation is important (Figure 14 (Fig. 14)). The postseptal approach to the orbital rim is more direct whereas the preseptal preparation avoids a prolapse of the orbital fatty tissue [323]. With the transconjunctival approach through the inferior fornix, the infraorbital rim, the orbital floor, and the superior edge of the maxilla can be exposed. Via a pre- or transcaruncular incision, additionally the medial orbital wall can be exposed. In many cases, lateral canthotomy is necessary for better overview of the lateral orbital wall. With this extension of the approach, even the area of the zygomatico-frontal suture can be reached. Generally, all transconjunctival approaches bear the risk of entropion development [322]. A subtle surgical technique and accurate wound closure help avoiding those complications. Generally cornea protection has to be used for transconjunctival approaches. 

###### Coronal approach (coronal incision)

If an open exposition of the zygomatic arch is needed, the direct transcutaneous approach is not recommended. The risk of damage of the frontal branch of the facial nerve is extremely high. Instead, a coronal access is used to expose the zygomatic region. In traumatology of the midface and the fronto-orbital region, the coronal approach is universally applicable. It allows exposing the complete calvaria, the anterior and lateral skull base, the region of the frontal and ethmoid sinuses, the lateral aspect of the zygomatic bone, the zygomatic arch, the superior part of the orbit, the nasal bones, the area of the mandibular joint, and the processes of the mandibular joint. The transection of the skin and the subcutaneous tissue is performed behind the hairline to avoid visible scars. For bald men, the coronal incision can be placed in the area of the back of the head. The frontal branch of the facial nerve is protected by dissecting the tissue below the superior layer of temporal fascia [324]. For exposition of the zygomatic arch, the incision has to be extended in caudal direction, either in pre- or postauricular direction. A detailed description of the surgical procedure of performance of coronal incisions is found in the AO Surgery Reference [54] and in a publication of Ellis and Zide [323].

For isolated exposition of the zygomatic arch, also a unilateral coronal incision can be performed. It allows a good overview and leads to acceptable esthetic results [87], [157].

### 5.2 Isolated zygomatic arch fractures

Fractures of the zygomatic arch that are associated with fractures of the zygomatic complex are treated in the context of repositioning and fixation of the zygomatic bone [13], [54]. Isolated fractures of the zygomatic bone have different appearances. M- or V-shaped impression. Fractures are typical due to direct trauma to the zygomatic arch. Due to the dislocation of the bony fragments in medial direction, the mobility of the muscular process of the mandible can be impaired. The clear sinking in the area of the zygomatic arch prominence and an impaired opening of the mouth are obvious. If an isolated fracture of the zygomatic arch is suspected, an axial imaging of the skull is helpful. The zygomatic arch is freely projected so that the assessment is facilitated [156].

The major part of dislocated isolated fractures of the zygomatic arch can be repositioned by transcutaneous closed procedure with the bone hook [163]. Especially in case of M- or V-shaped fractures of the zygomatic arch, a “clicking into place” of the fragments can be heard [160]. Generally those fragments remain stable without further fixation. For the closed repositioning, also a transoral (according to Keen) or a temporal procedure (according to Gillies) can be chosen [54]. If the repositioned zygomatic arch is likely to sink back into the dislocated position, an attempt is possible to stabilize the zygomatic arch in its position by means of an external splint to which the zygomatic fragments are fixed with deep sutures [159], [233]. An external splint that is fixed of the repositioned zygomatic arch also protects the fracture area [54]. It is helpful to assess the result of repositioning to perform intraoperative radiological 3D controls [325]. More complex and very instable fractures of the zygomatic arch may require an open exposition of the fracture and osteosynthesis. In this context, a preauricular approach, probably with use of an endoscope, or a coronal incision are chosen [54], [326].

## 6 Pan-facial fractures

Pan-facial fractures concern the superior, middle, and inferior third of the facial skull [327]. They are a challenge for the responsible team because often important reference points for reconstruction of the facial skull are missing and at the same time a multitude of difficult concomitant findings such as massive comminution, loss of tissue, and intensive bleedings are found. So for every patient with pan-facial trauma an individual treatment concept must be established in order to reconstruct the facial skull in its correct height, width, and projection.

The origin of pan-facial fractures is mostly high-energy trauma that consist of a primary force to the face and additionally of secondary or contrecoup forces leading to relevant injuries. According to the current literature, about 4–10% of all fractures of the facial skull are pan-facial fractures. They are mainly caused by traffic accidents or gunshots [327]. In 80% of pan-facial fractures, fractures of the joint head or the articular process of the mandible are concerned beside the midfacial fractures. Often even the dental arch of the maxilla and the mandible are affected and thus reconstruction of the occlusion and the original width of the face is particularly difficult. A sagittal fracture of the maxilla and a so-called open-book fracture of the mandibular arch with bilateral fractures of the articular processes in combination with paramedian and median fractures of the mandible lead to a loss of the transversal dimension [76].

The structure of the facial skull is characterized by the course of the vertical and horizontal trajectories (Figure 1 (Fig. 1)). The accurate reconstruction of those bone pillars is crucial for the treatment of pan-facial fractures. The following parameters must be observed [327]:

- Preservation of the projection and protection of the airways
- Preservation of the anchorage of the musculo-aponeurotical system
- Protection of important structures and functions:
  - Frontal brain
  - Eyes and vision
  - Neurovascular entrances
  - Oropharyngeal mechanisms for speaking, chewing, and swallowing
- Reconstruction of the original height, width, and projection of the facial skull
- Reconstruction of the following vertical trajectories: naso-maxillary, zygomatico-maxillary, auricular process, and ascending branch of the mandible
  - The pterygo-maxillary pillar is usually not reconstructed because of the poor accessibility.
- Reconstruction of the following horizontal trajectories: frontal, zygomatic bone, maxillary, mandibular
- The surgeon has to bear in mind that the horizontal trajectories of the central midface are physiologically relatively weak and after fracture there is the risk of losing the projection.

The following principles of reconstruction are further recommended [327]:

- Prioritization of the functional preservation of brain, eyes, and hearing
- Stabilization of open mandibular fractures as soon as possible
- Support of the pillar structures of the midface (splints, support) until definitive treatment can be performed
- Preservation of the integrity of soft tissue and subunits of the face
  - Neurovascular structures and tracts
  - Cranial nerves
  - Lacrimal ducts
- Careful planning of fracture treatment (course)
- Planning of probably necessary bone transplantation
- Final reconstruction of soft tissue

For the definite procedure of surgical treatment of fractures there are several possibilities. For many pan-facial fractures the primary reconstruction of the occlusion turned out to be useful. In the context of this bottom-up procedure, first both dental arches are reconstructed in their original width and toothing and from there in upward direction the fractures midfacial structures are reconstructed. Patients with pan-facial fractures with simultaneous fracture of the mandibular symphysis (and preserved dentition) it may be appropriate to first treat the fracture of the symphysis in order to re-establish the width of the dental arch. On this basis, the comminuted and sometimes extended arch of the maxilla can be reconstructed in the Le Fort level and stabilized with osteosynthetic plates or via mandibular-maxillary fixation [328].

In the context of the top-down procedure the stable fronto-orbital frame is taken as reference for the reconstruction of the midface in caudal direction. The advantage is that in case of fractures of the auricular processes the risk is lower to fix them in an incorrect position. Generally, it is not appropriate to stick dogmatically to one strategy. The experienced surgeon uses the existing stable bony elements as references and from there he approaches, sometimes from different sides, the comminuted or defect areas.

A typical surgical procedure for pan-facial fractures could be:

- Reconstruction of the frontal and supraorbital frame (via coronal incision)
- Reconstruction of the lateral orbital structures, the zygomatic bones and arches
- Assembling of the sagittally fractured maxilla and establishing the occlusion (MMF), if necessary the mandibular factures are treated previously
- Reconstruction of the other frame and wall structures of the orbits
- Reconstruction of the zygomatico-alveolar crest (if needed with bone transplantations)
- Reconstruction of the nasal projection

In summary, the treatment of pan-facial fractures of the facial skull still represents an major challenge and often requires an interdisciplinary strategy. The primary objective is the accurate reconstruction of the cranio-facial structures in all three dimensions and preservation of all relevant functions in this area (brain, vision, hearing, swallowing, chewing, smelling, mimic, and esthetic).

## Notes

### Competing interests

The authors declare that they have no competing interests.

## Figures and Tables

**Table 1:**
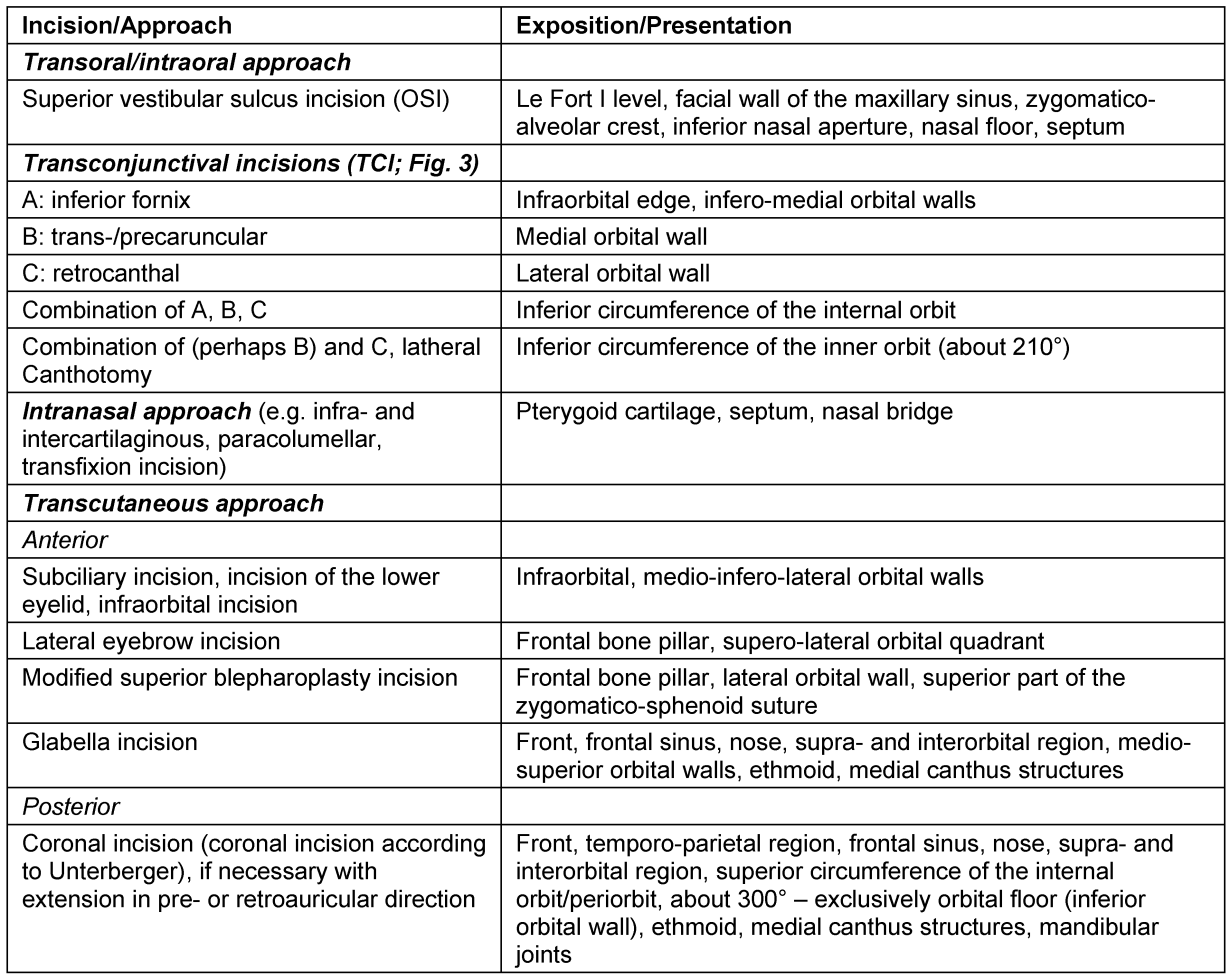
Surgical approaches to the facial skull, modified according to [13]

**Table 2:**
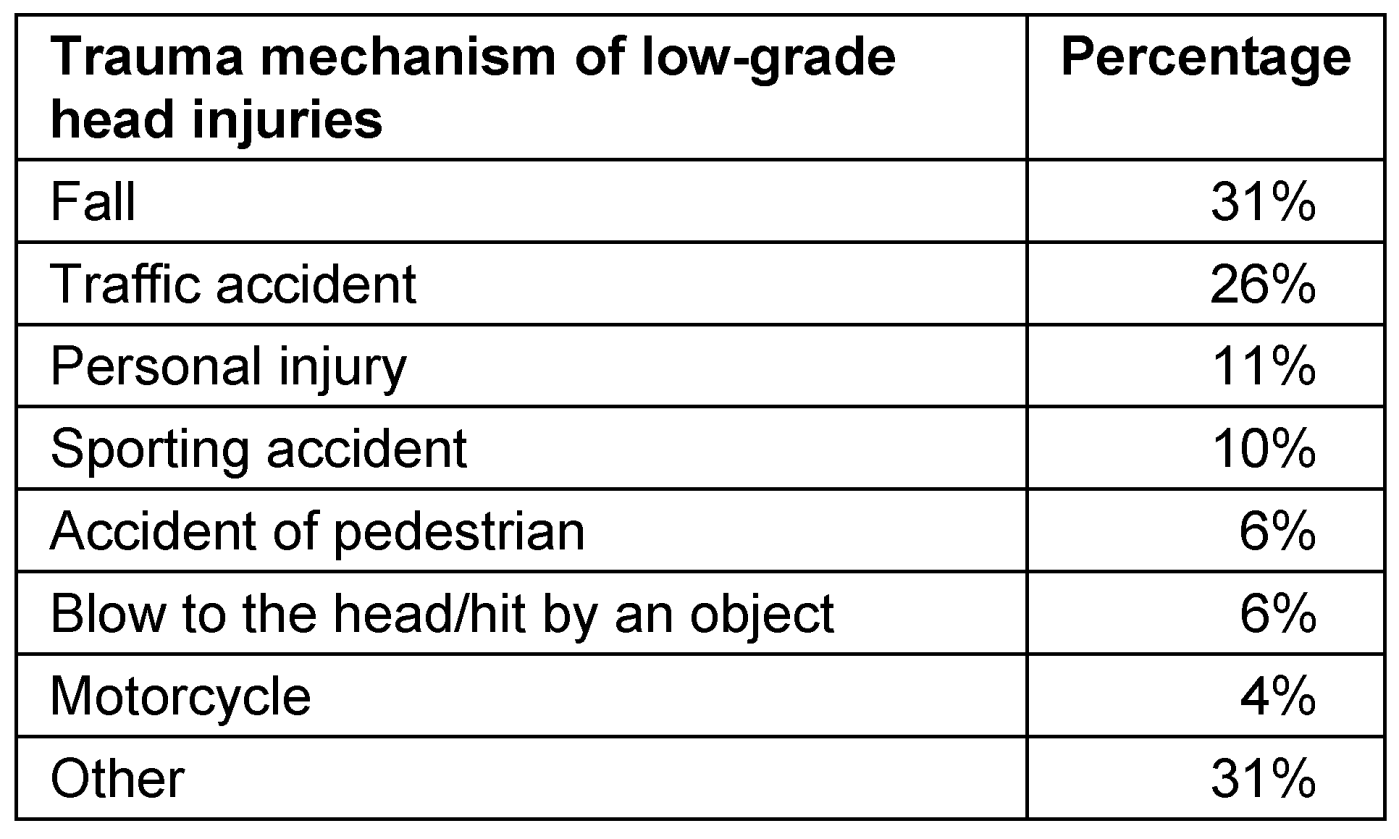
Low-grade head injuries [38]

**Table 3:**
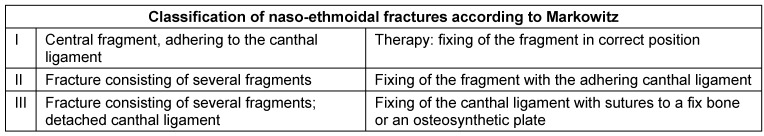
Classification according to Markowitz [91]

**Table 4:**
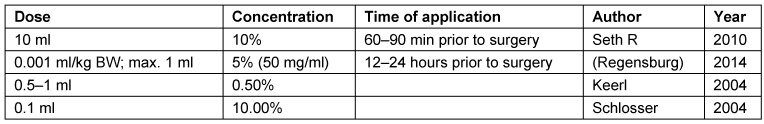
Fluorescein application for detection of CSF leaks

**Table 5:**
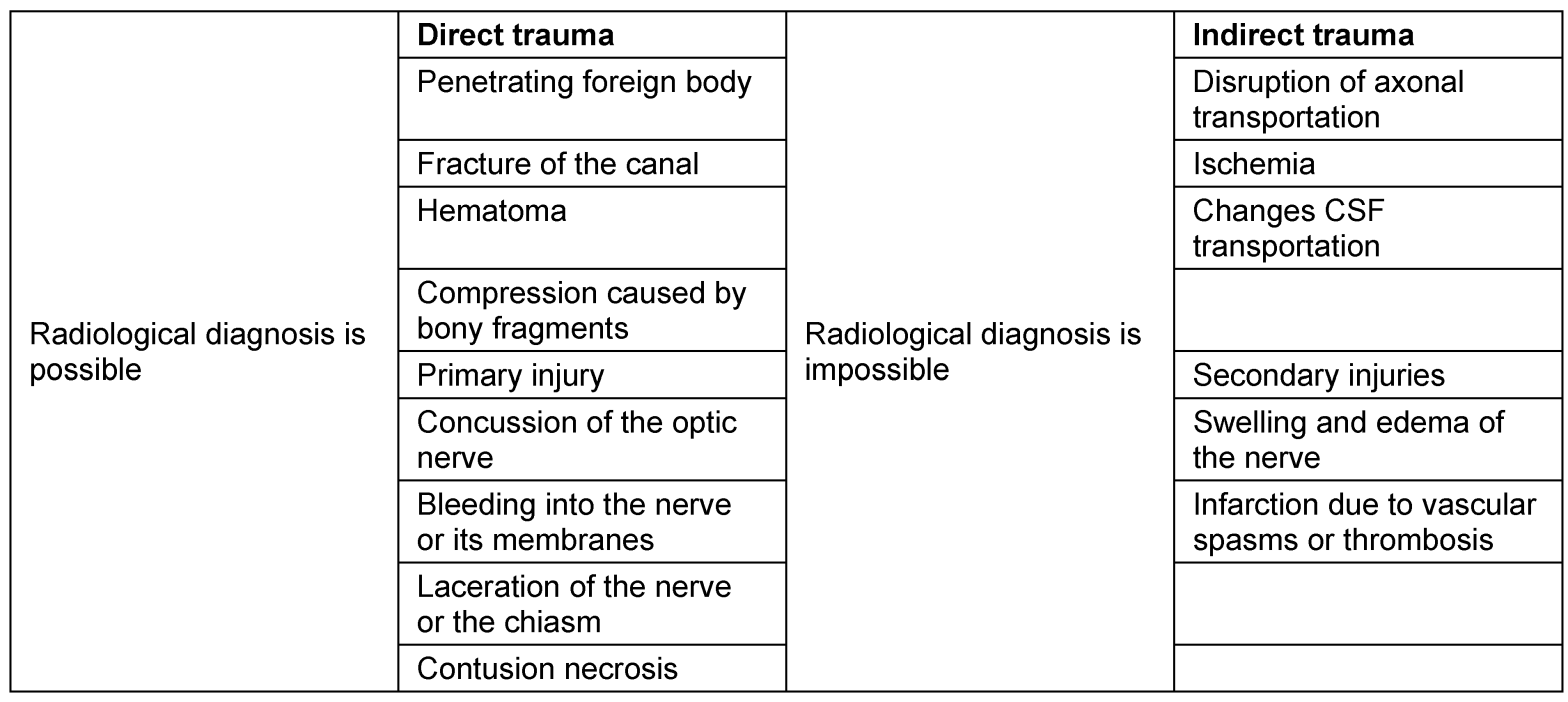
Patterns of injury of the optic nerve according to Walsh [263]

**Table 6:**
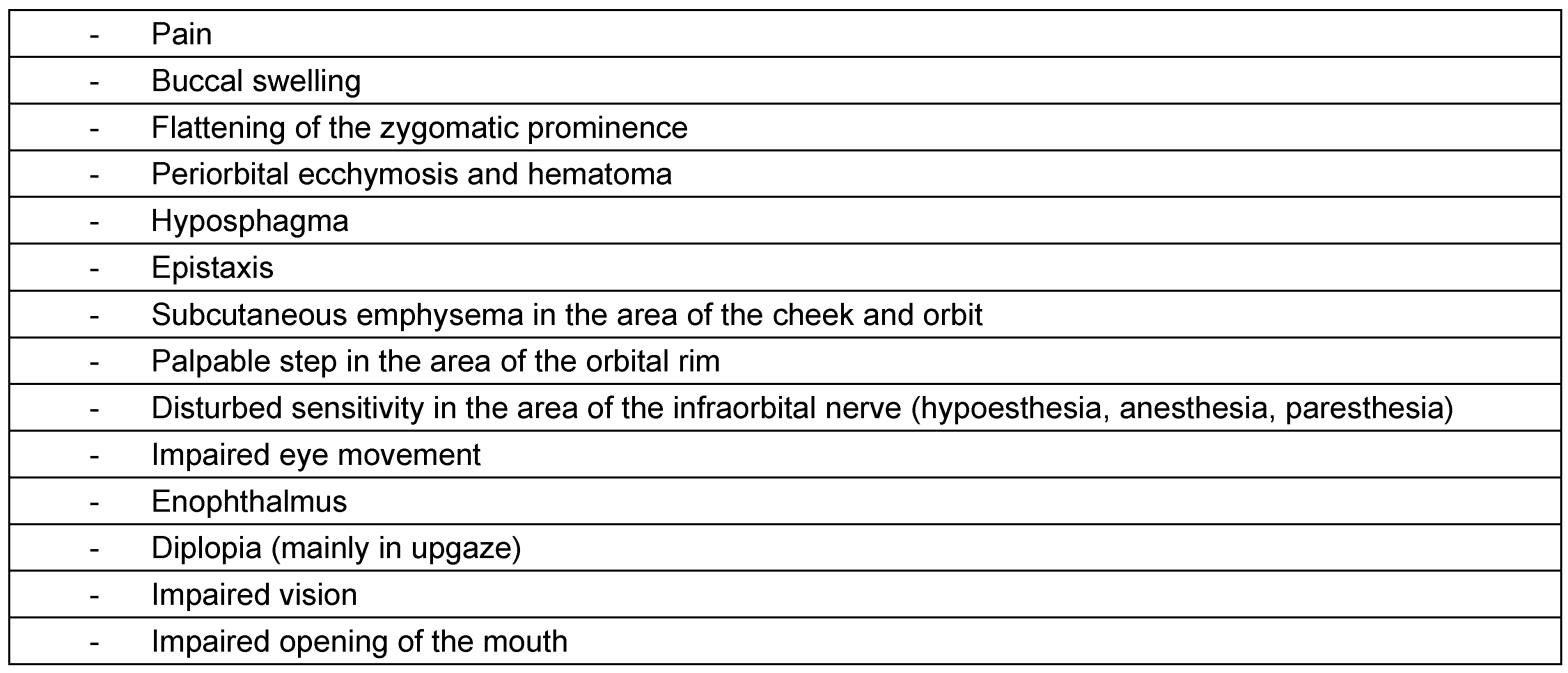
Possible clinical symptoms in cases of fractures of the zygomatic bone complex

## References

[1] Maier H, Tisch M, Lorenz KJ, Danz B, Schramm A (2011). Penetrierende Gesichts- und Halsverletzungen. Diagnostik und Therapie. HNO.

[2] Frodel JL (2012). Avoiding and correcting complications in perinasal trauma. Facial Plast Surg.

[3] Alvi A, Doherty T, Lewen G (2003). Facial fractures and concomitant injuries in trauma patients. Laryngoscope.

[4] Buchanan EP, Hopper RA, Suver DW, Hayes AG, Gruss JS, Birgfeld CB (2012). Zygomaticomaxillary complex fractures and their association with naso-orbito-ethmoid fractures: a 5-year review. Plast Reconstr Surg.

[5] Walters JT, Bisson JI, Shepherd JP (2007). Predicting post-traumatic stress disorder: validation of the Trauma Screening Questionnaire in victims of assault. Psychol Med.

[6] Bisson JI, Shepherd JP (1995). Psychological reactions of victims of violent crime. Br J Psychiatry.

[7] Motamedi MH (2003). An assessment of maxillofacial fractures: a 5-year study of 237 patients. J Oral Maxillofac Surg.

[8] Motamedi MH, Dadgar E, Ebrahimi A, Shirani G, Haghighat A, Jamalpour MR (2014). Pattern of maxillofacial fractures: a 5-year analysis of 8,818 patients. J Trauma Acute Care Surg.

[9] Martinez AY, Como JJ, Vacca M, Nowak MJ, Thomas CL, Claridge JA (2014). Trends in maxillofacial trauma: a comparison of two cohorts of patients at a single institution 20 years apart. J Oral Maxillofac Surg.

[10] van Beek GJ, Merkx CA (1999). Changes in the pattern of fractures of the maxillofacial skeleton. Int J Oral Maxillofac Surg.

[11] Bonkowsky VM, Mang WL, Wendl F, Frank C (1989). Neurologische Komplikationen bei Mittelgesichtsfrakturen. Laryngorhinootologie.

[12] Hollier LH, Sharabi SE, Koshy JC, Stal S (2010). Facial trauma: general principles of management. J Craniofac Surg.

[13] Mast G, Ehrenfeld M, Cornelius CP (2012). Maxillofaziale Frakturen: Mittelgesicht und interne Orbita. Teil 2: Therapieoptionen. Unfallchirurg.

[14] Gentile MA, Tellington AJ, Burke WJ, Jaskolka MS (2013). Management of midface maxillofacial trauma. Atlas Oral Maxillofac Surg Clin North Am.

[15] Clauser L, Dallera V, Sarti E, Tieghi R (2004). Frontobasilar fractures in children. Childs Nerv Syst.

[16] Schünke M, Schulte E, Schumacher U, Voll M, Wesker K Kopf, Hals und Neuroanatomie.

[17] Rasse M, Hausamen JE, Reuther JF, Eufinger H, Kübler A, Schliephake H (2012). Spezielle Traumatologie. Mund-, Kiefer- und Gesichtschirurgie.

[18] Hardt N, Kuttenberger J (2010). Craniofacial Trauma.

[19] Campbell CA, Lin KY (2009). Complications of rigid internal fixation. Craniomaxillofac Trauma Reconstr.

[20] Zingg M, Laedrach K, Chen J, Chowdhury K, Vuillemin T, Sutter F, Raveh J (1992). Classification and treatment of zygomatic fractures: a review of 1,025 cases. J Oral Maxillofac Surg.

[21] Le Fort R (1901). Etude expérimentale sur les fractures de la mâchoire inférieure. I, II, III. Rev Chir Paris.

[22] Kochhar A, Byrne PJ (2013). Surgical management of complex midfacial fractures. Otolaryngol Clin North Am.

[23] Mueller R (2008). Endoscopic treatment of facial fractures. Facial Plast Surg.

[24] Metzger MC, Schön R, Weyer N, Rafii A, Gellrich NC, Schmelzeisen R, Strong BE (2006). Anatomical 3-dimensional pre-bent titanium implant for orbital floor fractures. Ophthalmology.

[25] Kokemüller H, von See C, Essig H, Tavassol F, Rücker M, Schramm A, Majdani O, Gellrich NC (2011). Rekonstruktion komplexer Mittelgesichtsdefekte durch individualisierte Titanimplantate. HNO.

[26] Newman F, Cillo JE (2008). Late vascular complication associated with panfacial fractures. J Oral Maxillofac Surg.

[27] Biffl WL, Moore EE, Offner PJ, Brega KE, Franciose RJ, Elliott JP, Burch JM (1999). Optimizing screening for blunt cerebrovascular injuries. Am J Surg.

[28] Sun GH, Shoman NM, Samy RN, Pensak ML (2011). Analysis of carotid artery injury in patients with basilar skull fractures. Otol Neurotol.

[29] Wei CW, Montanera W, Selchen D, Lian J, Stevens C, de Tilly LN (2010). Blunt cerebrovascular injuries: diagnosis and management outcomes. Can J Neurol Sci.

[30] Kovács AF, Ghahremani M (2001). Minimization of zygomatic complex fracture treatment. Int J Oral Maxillofac Surg.

[31] Scolozzi P, Terzic A (2011). "Mirroring" computational planning, navigation guidance system, and intraoperative mobile C-arm cone-beam computed tomography with flat-panel detector: a new rationale in primary and secondary treatment of midfacial fractures? J Oral Maxillofac Surg. http://dx.doi.org/10.1016/j.joms.2010.07.049.

[32] Samii M, Tatagiba M (2002). Skull base trauma: diagnosis and management. Neurol Res.

[33] Aguiar GB (2014). Endovascular treatment of the carotid-cavernous vascular lesions. Arq Neuropsiquiatr.

[34] Malan J, Lefeuvre D, Mngomezulu V, Taylor A (2012). Angioarchitecture and treatment modalities in posttraumatic carotid cavernous fistulae. Interv Neuroradiol.

[35] Ruff IM, Strozyk D, Rahman C, Szeder V, Pile-Spellman J, Marshall RS (2010). Clinical reasoning: a 21-year-old woman with right eye swelling and bruising. Neurology.

[36] Sollberger M, Lyrer P, Baumann T, Radü EW, Steck AJ, Wetzel SG (2005). Isolated bilateral abducent nerve palsy due to a spontaneous left-side dural carotid cavernous fistula Type Barrow C. J Neurol.

[37] Fenzl CR, Golio D (2014). The impact of suction drainage on orbital compartment syndrome after craniofacial surgery. J Craniofac Surg.

[38] Bogusiak K, Arkuszewski P (2010). Characteristics and epidemiology of zygomaticomaxillary complex fractures. J Craniofac Surg.

[39] Salentijn EG, Boverhoff J, Heymans MW, van den Bergh B, Forouzanfar T (2014). The clinical and radiographical characteristics of zygomatic complex fractures: a comparison between the surgically and non-surgically treated patients. J Craniomaxillofac Surg.

[40] Chadha NK, Repanos C, Carswell AJ (2009). Local anaesthesia for manipulation of nasal fractures: systematic review. J Laryngol Otol.

[41] Robiony M, Tenani G, Bellini P, Salgarelli AC (2012). Intraoral approach for aesthetic restoration of posttraumatic zygomatic arch deformities. J Craniofac Surg.

[42] Krishnan B, El Sheikh MH (2008). Dental forceps reduction of depressed zygomatic arch fractures. J Craniofac Surg.

[43] Lock R (2010). Managing the difficult airway in craniomaxillofacial trauma. Craniomaxillofac Trauma Reconstr.

[44] Lee SS, Huang SH, Wu SH, Sun IF, Chu KS, Lai CS, Chen YL (2009). A review of intraoperative airway management for midface facial bone fracture patients. Ann Plast Surg.

[45] Lima SM, Asprino L, Moreira RW, de Moraes M (2011). A retrospective analysis of submental intubation in maxillofacial trauma patients. J Oral Maxillofac Surg.

[46] Jundt JS, Cattano D, Hagberg CA, Wilson JW (2012). Submental intubation: a literature review. Int J Oral Maxillofac Surg.

[47] Gelesko S, Markiewicz MR, Bell RB (2013). Responsible and prudent imaging in the diagnosis and management of facial fractures. Oral Maxillofac Surg Clin North Am.

[48] Evans GR, Clark N, Manson PN, Leipziger LS (1995). Role of mini- and microplate fixation in fractures of the midface and mandible. Ann Plast Surg.

[49] Meslemani D, Kellman RM (2012). Recent advances in fixation of the craniomaxillofacial skeleton. Curr Opin Otolaryngol Head Neck Surg.

[50] Avashia YJ, Sastry A, Fan KL, Mir HS, Thaller SR (2012). Materials used for reconstruction after orbital floor fracture. J Craniofac Surg.

[51] Turvey TA, Proffit WP, Phillips C (2011). Biodegradable fixation for craniomaxillofacial surgery: a 10-year experience involving 761 operations and 745 patients. Int J Oral Maxillofac Surg.

[52] Dingman RO, Grabb WC, Oneal RM (1969). Management of injuries of the naso-orbital complex. Arch Surg.

[53] Reichwein A, Schicho K, Moser D, Seemann R, Poeschl P, Baumann A, Ewers R (2009). Clinical experiences with resorbable ultrasonic-guided, angle-stable osteosynthesis in the panfacial region. J Oral Maxillofac Surg.

[54] Cornelius CP, Gellrich N, Hillerup S, Kusumoto K, Schubert W, Fusetti S (2013). AO Surgery Reference.

[55] (2012). Perioperative Antibiotikaprophylaxe. AWMF Registernummer: 029/022 (Klasse S1 + IDA). AWMF Leitlinie.

[56] (2006). Antimicrobial prophylaxis for surgery. Treat Guidel Med Lett.

[57] Classen DC, Evans RS, Pestotnik SL, Horn SD, Menlove RL, Burke JP (1992). The timing of prophylactic administration of antibiotics and the risk of surgical-wound infection. N Engl J Med.

[58] Lauder A, Jalisi S, Spiegel J, Stram J, Devaiah A (2010). Antibiotic prophylaxis in the management of complex midface and frontal sinus trauma. Laryngoscope.

[59] Hauser CJ, Adams CA, Eachempati SR, Council of the Surgical Infection Society (2006). Surgical Infection Society guideline: prophylactic antibiotic use in open fractures: an evidence-based guideline. Surg Infect (Larchmt).

[60] Sandner A, Kern CB, Bloching MB (2006). Erfahrungen mit dem subfrontalen Zugang zur Rekonstruktion ausgedehnter Frakturen im Bereich der Frontobasis. Laryngorhinootologie.

[61] Raveh J, Vuillemin T (1988). The surgical one-stage management of combined cranio-maxillo-facial and frontobasal fractures. Advantages of the subcranial approach in 374 cases. J Craniomaxillofac Surg.

[62] Hosemann W, Schroeder HW, Kaduk W, Augst D, Friedrich J (2005). Interdisziplinäres Management von Mittelgesichtsverletzungen. HNO.

[63] Han DS, Han YS, Park JH (2011). A new approach to the treatment of nasal bone fracture: radiologic classification of nasal bone fractures and its clinical application. J Oral Maxillofac Surg.

[64] Atighechi S, Karimi G (2009). Serial nasal bone reduction: a new approach to the management of nasal bone fracture. J Craniofac Surg.

[65] Wright RJ, Murakami CS, Ambro BT (2011). Pediatric nasal injuries and management. Facial Plast Surg.

[66] Gharehdaghi J, Samadi Rad B, Ghatreh Samani V, Kolahi F, Khatami Zonoozian A, Marashian SM (2013). Comparison of physical examination and conventional radiography in diagnosis of nasal fracture. Indian J Otolaryngol Head Neck Surg.

[67] Rhee SC, Kim YK, Cha JH, Kang SR, Park HS (2004). Septal fracture in simple nasal bone fracture. Plast Reconstr Surg.

[68] Strutz J, Mann W (2010). Praxis der HNO-Heilkunde, Kopf- und Halschirurgie.

[69] Alvarez H, Osorio J, De Diego JI, Prim MP, De La Torre C, Gavilan J (2000). Sequelae after nasal septum injuries in children. Auris Nasus Larynx.

[70] Dennis SC, den Herder C, Shandilya M, Nolst Trenité GJ (2007). Open rhinoplasty in children. Facial Plast Surg.

[71] Mayorga O, Steele N, Fried MP (2006). Nasal fracture reduction. Medscape.

[72] Smith JE, Perez CL (2009). Nasal Fractures.

[73] Desrosiers AE, Thaller SR (2011). Pediatric nasal fractures: evaluation and management. J Craniofac Surg.

[74] Ernst A, Herzog M, Saidl RO (2004). Traumatologie des Kopf-Hals-Bereiches.

[75] Bell RB (2010). Computer planning and intraoperative navigation in cranio-maxillofacial surgery. Oral Maxillofac Surg Clin North Am.

[76] Schiel S, Smolka W, Leiggener C, Kaeppler G, Cornelius CP (2012). „Open book“ – Frakturen des Mandibularbogens: Bilaterale Gelenkfortsatzfrakturen in Kombination mit Paramedian-/Medianfrakturen des Unterkieferscases. Operative Behandlungsstrategien. OP-Journal.

[77] Vestergaard V, Astrand R, Romner B (2014). A survey of the management of paediatric minor head injury. Acta Neurol Scand.

[78] Hawley CA, Ward AB, Magnay AR, Long J (2002). Children's brain injury: a postal follow-up of 525 children from one health region in the UK. Brain Inj.

[79] Ginsburg CM, Leach JL (1995). Infected nasal septal hematoma. Pediatr Infect Dis J.

[80] Canty PA, Berkowitz RG (1996). Hematoma and abscess of the nasal septum in children. Arch Otolaryngol Head Neck Surg.

[81] Oluwasanmi AF, Pinto AL (2000). Management of nasal trauma--widespread misuse of radiographs. Clin Perform Qual Health Care.

[82] Ellis E, Figari M, Aniceto GS, Shimozato K AO Surgery Reference: Online Reference in clinical life.

[83] Verwoerd CD, Verwoerd-Verhoef HL (2007). Rhinosurgery in children: basic concepts. Facial Plast Surg.

[84] Schwenzer N, Ehrenfeld M (2011). Zahn-Mund-Kiefer-Heilkunde. Mund-Kiefer-Gesichtschirurgie.

[85] Sargent LA (2007). Nasoethmoid orbital fractures: diagnosis and treatment. Plast Reconstr Surg.

[86] Elbarbary AS, Ali A (2014). Medial canthopexy of old unrepaired naso-orbito-ethmoidal (noe) traumatic telecanthus. J Craniomaxillofac Surg.

[87] Kharkar VR, Rudagi BM, Kini Y (2010). Modification of the hemicoronal approach to treat fractures of the zygomatic complex. J Maxillofac Oral Surg.

[88] Rosenberger E, Kriet JD, Humphrey C (2013). Management of nasoethmoid fractures. Curr Opin Otolaryngol Head Neck Surg.

[89] Herford AS, Ying T, Brown B (2005). Outcomes of severely comminuted (type III) nasoorbitoethmoid fractures. J Oral Maxillofac Surg.

[90] Morrison AD, Gregoire CE (2013). Management of fractures of the nasofrontal complex. Oral Maxillofac Surg Clin North Am.

[91] Markowitz BL, Manson PN, Sargent L, Vander Kolk CA, Yaremchuk M, Glassman D, Crawley WA (1991). Management of the medial canthal tendon in nasoethmoid orbital fractures: the importance of the central fragment in classification and treatment. Plast Reconstr Surg.

[92] Yoshioka N, Tomita S, Nishikawa H, Arakawa A, Sesaki S (2014). Medial maxillary fractures revisited. J Plast Reconstr Aesthet Surg.

[93] Fedok FG (1995). Comprehensive management of nasoethmoid-orbital injuries. J Craniomaxillofac Trauma.

[94] He D, Zhang Y, Ellis E (2007). Panfacial fractures: analysis of 33 cases treated late. J Oral Maxillofac Surg.

[95] Hwang K, Kim HJ (2012). Making a malleable awl using a Steinmann pin for transnasal medial canthopexy. Ophthal Plast Reconstr Surg.

[96] Vora NM, Fedok FG (2000). Management of the central nasal support complex in naso-orbital ethmoid fractures. Facial Plast Surg.

[97] Engelstad ME, Bastodkar P, Markiewicz MR (2012). Medial canthopexy using transcaruncular barb and miniplate: technique and cadaver study. Int J Oral Maxillofac Surg.

[98] Papadopoulos H, Salib NK (2009). Management of naso-orbital-ethmoidal fractures. Oral Maxillofac Surg Clin North Am.

[99] Stranc MF, Robertson GA (1979). A classification of injuries of the nasal skeleton. Ann Plast Surg.

[100] Kim DW, Shah AR, Toriumi DM (2006). Concentric and eccentric carved costal cartilage: a comparison of warping. Arch Facial Plast Surg.

[101] Vaca EE, Mundinger GS, Kelamis JA, Dorafshar AH, Christy MR, Manson PN, Rodriguez ED (2013). Facial fractures with concomitant open globe injury: mechanisms and fracture patterns associated with blindness. Plast Reconstr Surg.

[102] Soong PL, Schaller B, Zix J, Iizuka T, Mottini M, Lieger O (2014). The role of postoperative prophylactic antibiotics in the treatment of facial fractures: a randomised, double-blind, placebo-controlled pilot clinical study. Part 3: Le Fort and zygomatic fractures in 94 patients. Br J Oral Maxillofac Surg.

[103] Molendijk J, van der Wal KG, Koudstaal MJ (2012). Surgical treatment of frontal sinus fractures: the simple percutaneous reduction revised. Int J Oral Maxillofac Surg.

[104] Weber R, Kühnel T, Graf J, Hosemann W (2014). Aerosinusitis. Teil 2: Diagnostik, Therapie und Wiederaufnahme der Flugtätigkeit. HNO.

[105] Kloss FR, Stigler RG, Brandstätter A, Tuli T, Rasse M, Laimer K, Hächl OL, Gassner R (2011). Complications related to midfacial fractures: operative versus non-surgical treatment. Int J Oral Maxillofac Surg.

[106] Kurita M, Okazaki M, Ozaki M, Tanaka Y, Tsuji N, Takushima A, Harii K (2010). Patient satisfaction after open reduction and internal fixation of zygomatic bone fractures. J Craniofac Surg.

[107] Lang J (1988). Klinische Anatomie der Nase, Nasenhöhle und Nebenhöhlen.

[108] Cornelius CP, Ehrenfeld M (2010). The Use of MMF Screws: Surgical Technique, Indications, Contraindications, and Common Problems in Review of the Literature. Craniomaxillofac Trauma Reconstr.

[109] Dalla Torre D, Burtscher D, Kloss-Brandstätter A, Rasse M, Kloss F (2014). Management of frontal sinus fractures--treatment decision based on metric dislocation extent. J Craniomaxillofac Surg.

[110] Sivori LA, de Leeuw R, Morgan I, Cunningham LL (2010). Complications of frontal sinus fractures with emphasis on chronic craniofacial pain and its treatment: a review of 43 cases. J Oral Maxillofac Surg.

[111] Salentijn EG, van den Bergh B, Forouzanfar T (2013). A ten-year analysis of midfacial fractures. J Craniomaxillofac Surg.

[112] Pollock RA, Hill JL, Davenport DL, Snow DC, Vasconez HC (2013). Cranialization in a cohort of 154 consecutive patients with frontal sinus fractures (1987-2007): review and update of a compelling procedure in the selected patient. Ann Plast Surg.

[113] Hollier LH, Thornton J, Pazmino P, Stal S (2003). The management of orbitozygomatic fractures. Plast Reconstr Surg.

[114] Chen W, Lu X (1999). Diagnosis and restitution of nasal bone fracture. Lin Chuang Er Bi Yan Hou Ke Za Zhi (=Journal of clinical otorhinolaryngology).

[115] Egemen O, Özkaya Ö, Aksan T, Bingöl D, Akan M (2013). Endoscopic repair of isolated anterior table frontal sinus fractures without fixation. J Craniofac Surg.

[116] Rodriguez ED, Stanwix MG, Nam AJ, St Hilaire H, Simmons OP, Christy MR, Grant MP, Manson PN (2008). Twenty-six-year experience treating frontal sinus fractures: a novel algorithm based on anatomical fracture pattern and failure of conventional techniques. Plast Reconstr Surg.

[117] Kahnberg KE, Göthberg KA (1987). Le Fort fractures (I). A study of frequency, etiology and treatment. Int J Oral Maxillofac Surg.

[118] Adeyemo WL, Taiwo OA, Ladeinde AL, Ogunlewe MO, Adeyemi MO, Adepoju AA (2012). Mid-facial fractures: a 5-year retrospective review in a Nigerian teaching hospital. Niger J Med.

[119] Hussain K, Wijetunge DB, Grubnic S, Jackson IT (1994). A comprehensive analysis of craniofacial trauma. J Trauma.

[120] Evans BG, Evans GR (2008). MOC-PSSM CME article: Zygomatic fractures. Plast Reconstr Surg.

[121] Weber A, May A, von Ilberg C, Klima A, Halbsguth A (1991). Die Computertomographie als Standarduntersuchungsverfahren zur Nasennebenhöhlendiagnostik aus der Sicht des Hals-Nasen-Ohren-Arztes. Ergebnisse. Laryngorhinootologie.

[122] Lund VJ, Savy L, Lloyd G, Howard D (2000). Optimum imaging and diagnosis of cerebrospinal fluid rhinorrhoea. J Laryngol Otol.

[123] Bush K, Huikeshoven M, Wong N (2013). Nasofrontal outflow tract visibility in computed tomography imaging of frontal sinus fractures. Craniomaxillofac Trauma Reconstr.

[124] Khan AM, Varvares MA (2006). Traditional approaches to the orbit. Otolaryngol Clin North Am.

[125] Gerbino G, Roccia F, Benech A, Caldarelli C (2000). Analysis of 158 frontal sinus fractures: current surgical management and complications. J Craniomaxillofac Surg.

[126] Strong EB, Kellman RM (2006). Endoscopic repair of anterior table--frontal sinus fractures. Facial Plast Surg Clin North Am.

[127] Olate S, Lima SM, Sawazaki R, Moreira RW, de Moraes M (2010). Surgical approaches and fixation patterns in zygomatic complex fractures. J Craniofac Surg.

[128] Strong EB (2009). Frontal sinus fractures: current concepts. Craniomaxillofac Trauma Reconstr.

[129] Xie C, Mehendale N, Barrett D, Bui CJ, Metzinger SE (2000). 30-year retrospective review of frontal sinus fractures: The Charity Hospital experience. J Craniomaxillofac Trauma.

[130] Wilson BC, Davidson B, Corey JP, Haydon RC (1988). Comparison of complications following frontal sinus fractures managed with exploration with or without obliteration over 10 years. Laryngoscope.

[131] Kalavrezos N (2004). Current trends in the management of frontal sinus fractures. Injury.

[132] Kim KS, Kim ES, Hwang JH, Lee SY (2010). Transcutaneous transfrontal approach through a small peri-eyebrow incision for the reduction of closed anterior table frontal sinus fractures. J Plast Reconstr Aesthet Surg.

[133] Rao J, Blackburn TK, Clark S, Musgrove BT (2013). Upper eyelid incision and use of a 90° screwdriver for osteosynthesis of fractures of the anterior table of the frontal sinus. Br J Oral Maxillofac Surg.

[134] Brasileiro BF, Cortez AL, Asprino L, Passeri LA, De Moraes M, Mazzonetto R, Moreira RW (2005). Traumatic subcutaneous emphysema of the face associated with paranasal sinus fractures: a prospective study. J Oral Maxillofac Surg.

[135] Kim DW, Yoon ES, Lee BI, Dhong ES, Park SH (2012). Fracture depth and delayed contour deformity in frontal sinus anterior wall fracture. J Craniofac Surg.

[136] Thaller SR, Donald P (1994). The use of pericranial flaps in frontal sinus fractures. Ann Plast Surg.

[137] Yavuzer R, Sari A, Kelly CP, Tuncer S, Latifoglu O, Celebi MC, Jackson IT (2005). Management of frontal sinus fractures. Plast Reconstr Surg.

[138] Maturo SC, Weitzel EK, Cowhart J, Brennan J (2008). Isolated posterior table frontal sinus fractures do not form mucoceles in a goat model. Otolaryngol Head Neck Surg.

[139] Pohlenz P, Blessmann M, Blake F, Heinrich S, Schmelzle R, Heiland M (2007). Clinical indications and perspectives for intraoperative cone-beam computed tomography in oral and maxillofacial surgery. Oral Surg Oral Med Oral Pathol Oral Radiol Endod.

[140] Ting JY, Wu A, Metson R (2014). Frontal sinus drillout (modified Lothrop procedure): long-term results in 204 patients. Laryngoscope.

[141] Rohrich RJ, Hollier L (1996). The role of the nasofrontal duct in frontal sinus fracture management. J Craniomaxillofac Trauma.

[142] Gerber B, Kiwanuka P, Dhariwal D (2013). Orbital fractures in children: a review of outcomes. Br J Oral Maxillofac Surg.

[143] Day TA, Meehan R, Stucker FJ, Nanda A (1998). Management of frontal sinus fractures with posterior table involvement: a retrospective study. J Craniomaxillofac Trauma.

[144] Yakirevitch A, Bedrin L, Alon EE, Yoffe T, Wolf M, Yahalom R (2013). Relation between preoperative computed tomographic criteria of injury to the nasofrontal outflow tract and operative findings in fractures of the frontal sinus. Br J Oral Maxillofac Surg.

[145] Magarakis M, Mundinger GS, Kelamis JA, Dorafshar AH, Bojovic B, Rodriguez ED (2012). Ocular injury, visual impairment, and blindness associated with facial fractures: a systematic literature review. Plast Reconstr Surg.

[146] Rodriguez ED, Stanwix MG, Nam AJ, St Hilaire H, Simmons OP, Manson PN (2009). Definitive treatment of persistent frontal sinus infections: elimination of dead space and sinonasal communication. Plast Reconstr Surg.

[147] Moore I (1920). Left Frontal Sinus Suppuration; Radical (Killian) Operation. Proc R Soc Med.

[148] Tilley H (1914). Discussion on the Intranasal Operative Treatment of Frontal Sinus: (II) Introductory Paper. Proc R Soc Med.

[149] Cho RI, Davies BW (2013). Combined orbital floor and medial wall fractures involving the inferomedial strut: repair technique and case series using preshaped porous polyethylene/titanium implants. Craniomaxillofac Trauma Reconstr.

[150] Kim JH, Lee JH, Hong SM, Park CH (2012). The effectiveness of 1-point fixation for zygomaticomaxillary complex fractures. Arch Otolaryngol Head Neck Surg.

[151] Donald PJ (1982). Frontal sinus ablation by cranialization. Report of 21 cases. Arch Otolaryngol.

[152] Carter KB, Poetker DM, Rhee JS (2010). Sinus preservation management for frontal sinus fractures in the endoscopic sinus surgery era: a systematic review. Craniomaxillofac Trauma Reconstr.

[153] Guy WM, Brissett AE (2013). Contemporary management of traumatic fractures of the frontal sinus. Otolaryngol Clin North Am.

[154] Jamal BT, Pfahler SM, Lane KA, Bilyk JR, Pribitkin EA, Diecidue RJ, Taub DI (2009). Ophthalmic injuries in patients with zygomaticomaxillary complex fractures requiring surgical repair. J Oral Maxillofac Surg.

[155] Metzinger SE, Metzinger RC (2009). Complications of frontal sinus fractures. Craniomaxillofac Trauma Reconstr.

[156] Nakib S, Schön R (2014). Mittelgesichtsfrakturen. MKG-Chirurg.

[157] Kharkar VR, Rudagi BM, Halli R, Kini Y (2010). Comparison of the modified lateral orbitotomy approach and modified hemicoronal approach in the treatment of unstable malunions of zygomatic complex fractures. Oral Surg Oral Med Oral Pathol Oral Radiol Endod.

[158] Weber R, Draf W, Keerl R, Constantinidis J (1995). Aspekte zur Stimmhöhlenchirurgie. Teil III: Indikation und Ergebnisse der osteoplastischen Stimmhöhlenoperation. HNO.

[159] Jones GM, Speculand B (1986). A splint for the unstable zygomatic arch fracture. Br J Oral Maxillofac Surg.

[160] Hwang K, Lee SI (1999). Reduction of zygomatic arch fracture using a towel clip. J Craniofac Surg.

[161] Pohlenz P, Blake F, Blessmann M, Smeets R, Habermann C, Begemann P, Schmelzle R, Heiland M (2009). Intraoperative cone-beam computed tomography in oral and maxillofacial surgery using a C-arm prototype: first clinical experiences after treatment of zygomaticomaxillary complex fractures. J Oral Maxillofac Surg.

[162] Vironneau P, Coste A, Prulière-Escabasse V (2014). Frontal sinus obliteration with autologous calvarial bone graft: indications and results. Eur Arch Otorhinolaryngol.

[163] Chidzonga MM (1989). Reduction of the isolated fracture of the zygomatic arch using a bone hook. Odontostomatol Trop.

[164] Kelley P, Hopper R, Gruss J (2007). Evaluation and treatment of zygomatic fractures. Plast Reconstr Surg.

[165] Weathers WM, Wolfswinkel EM, Hatef DA, Lee EI, Brown RH, Hollier LH (2013). Frontal sinus fractures: a conservative shift. Craniomaxillofac Trauma Reconstr.

[166] Kuhn FA (2006). An integrated approach to frontal sinus surgery. Otolaryngol Clin North Am.

[167] Davis WE (2005). Growing obsolescence of the frontal sinus obliteration procedure. Arch Otolaryngol Head Neck Surg.

[168] Smith TL, Han JK, Loehrl TA, Rhee JS (2002). Endoscopic management of the frontal recess in frontal sinus fractures: a shift in the paradigm?. Laryngoscope.

[169] Litschel R, Tasman AJ (2009). Stirnhöhlenfraktur: aktuelle Kontroversen zur Therapie. Laryngorhinootologie.

[170] Bell RB, Dierks EJ, Brar P, Potter JK, Potter BE (2007). A protocol for the management of frontal sinus fractures emphasizing sinus preservation. J Oral Maxillofac Surg.

[171] Hegazy HM, Carrau RL, Snyderman CH, Kassam A, Zweig J (2000). Transnasal endoscopic repair of cerebrospinal fluid rhinorrhea: a meta-analysis. Laryngoscope.

[172] van Issum C, Courvoisier DS, Scolozzi P (2013). Posttraumatic orbital emphysema: incidence, topographic classification and possible pathophysiologic mechanisms. A retrospective study of 137 patients. Oral Surg Oral Med Oral Pathol Oral Radiol.

[173] Hosemann W, Gottsauner A, Leuwer A, Farmand M, Wenning W, Göde U, Stenglein C, von Glass W (1993). Untersuchungen zur Frakturheilung im Siebbein--Ein Beitrag zur rhinologischen Versorgung nasoethmoidaler Verletzungen. Laryngorhinootologie.

[174] Gjuric M, Goede U, Keimer H, Wigand ME (1996). Endonasal endoscopic closure of cerebrospinal fluid fistulas at the anterior cranial base. Ann Otol Rhinol Laryngol.

[175] Yeo NK, Cho GS, Kim CJ, Lim GC, Jang YJ, Lee BJ, Chung YS (2013). The effectiveness of lumbar drainage in the conservative and surgical treatment of traumatic cerebrospinal fluid rhinorrhea. Acta Otolaryngol.

[176] O'Brien MD, Reade PC (1984). The management of dural tear resulting from mid-facial fracture. Head Neck Surg.

[177] Dagi TF, GE G, Schmidek HH (1988). The management of cerebrospinal fluid leaks. Operative neurosurgical techniques.

[178] Neidhardt O (2002). Kraniofaziale und frontobasale Schädelverletzungen. Retrospektive Nachuntersuchung.

[179] Meco C, Oberascher G (2004). Comprehensive algorithm for skull base dural lesion and cerebrospinal fluid fistula diagnosis. Laryngoscope.

[180] Lund VJ, Stammberger H, Fokkens WJ, Beale T, Bernal-Sprekelsen M, Eloy P, Georgalas C, Gerstenberger C, Hellings P, Herman P, Hosemann WG, Jankowski R, Jones N, Jorissen M, Leunig A, Onerci M, Rimmer J, Rombaux P, Simmen D, Tomazic PV, Tschabitscherr M, Welge-Luessen A (2014). European position paper on the anatomical terminology of the internal nose and paranasal sinuses. Rhinol Suppl.

[181] Vajda L, Zahn W, Bonorden S (1987). Vergleichende Untersuchung zwischen computertomographischen Befunden und dem Operationssitus bei fronto-basalen Frakturen. Fortschr Kiefer Gesichtschir.

[182] Dietrich U, Feldges A, Sievers K, Kocks W (1993). Lokalisation von frontobasalen traumatischen Liquorfisteln. Ein Vergleich von radiologischem Befund und Operationsbefund. Zentralbl Neurochir.

[183] Kastenbauer E, Tardy ME, Naumann HH (1995). Gesicht, Nase und Gesichtsschädel. Kopf- und Halschirurgie.

[184] Ziu M, Savage JG, Jimenez DF (2012). Diagnosis and treatment of cerebrospinal fluid rhinorrhea following accidental traumatic anterior skull base fractures. Neurosurg Focus.

[185] Fangman B, Penna KJ (2012). Pneumomediastinum, pneumopericardium, orbital subcutaneous emphysema as consequence of low energy impact facial trauma. N Y State Dent J.

[186] Simmen D, Bischoff T (1998). Rhinochirurgisches Konzept zur Versorgung von Frontobasisdefekten mit Rhinoliquorrhoe. Laryngorhinootologie.

[187] Schlosser RJ, Bolger WE (2004). Nasal cerebrospinal fluid leaks: critical review and surgical considerations. Laryngoscope.

[188] Perheentupa U, Mäkitie AA, Kinnunen I (2014). Subcranial craniotomy approach for frontobasal fracture correction. J Craniomaxillofac Surg.

[189] Shetty PG, Shroff MM, Sahani DV, Kirtane MV (1998). Evaluation of high-resolution CT and MR cisternography in the diagnosis of cerebrospinal fluid fistula. AJNR Am J Neuroradiol.

[190] Sillers MJ, Morgan CE, el Gammal T (1997). Magnetic resonance cisternography and thin coronal computerized tomography in the evaluation of cerebrospinal fluid rhinorrhea. Am J Rhinol.

[191] Lantz EJ, Forbes GS, Brown ML, Laws ER (1980). Radiology of cerebrospinal fluid rhinorrhea. AJR Am J Roentgenol.

[192] Eljamel MS, Foy PM (1990). Acute traumatic CSF fistulae: the risk of intracranial infection. Br J Neurosurg.

[193] Kirchner FR, Proud GO (1960). Method for the identification and localization of cerebrospinal fluid, rhinorrhea and otorrhea. Laryngoscope.

[194] Seth R, Rajasekaran K, Benninger MS, Batra PS (2010). The utility of intrathecal fluorescein in cerebrospinal fluid leak repair. Otolaryngol Head Neck Surg.

[195] Demarco RC, Tamashiro E, Valera FC, Anselmo-Lima WT (2007). Use of a hypodense sodium fluorescein solution for the endoscopic repair of rhinogenic cerebrospinal fluid fistulae. Am J Rhinol.

[196] Keerl R, Weber RK, Draf W, Wienke A, Schaefer SD (2004). Use of sodium fluorescein solution for detection of cerebrospinal fluid fistulas: an analysis of 420 administrations and reported complications in Europe and the United States. Laryngoscope.

[197] Lewin W (1964). Cerebrospinal fluid rhinorrhea in nonmissile head injuries. Clin Neurosurg.

[198] Bell RB, Dierks EJ, Homer L, Potter BE (2004). Management of cerebrospinal fluid leak associated with craniomaxillofacial trauma. J Oral Maxillofac Surg.

[199] Mincy JE (1966). Posttraumatic cerebrospinal fluid fistula of the frontal fossa. J Trauma.

[200] Yilmazlar S, Arslan E, Kocaeli H, Dogan S, Aksoy K, Korfali E, Doygun M (2006). Cerebrospinal fluid leakage complicating skull base fractures: analysis of 81 cases. Neurosurg Rev.

[201] Hertel V, Schick B (2012). Diagnostik und Therapie von frontobasalen Liquorfisteln. Laryngorhinootologie.

[202] Lanza DC, O'Brien DA, Kennedy DW (1996). Endoscopic repair of cerebrospinal fluid fistulae and encephaloceles. Laryngoscope.

[203] Pease M, Marquez Y, Tuchman A, Markarian A, Zada G (2013). Diagnosis and surgical management of traumatic cerebrospinal fluid oculorrhea: case report and systematic review of the literature. J Neurol Surg Rep.

[204] Psaltis AJ, Schlosser RJ, Banks CA, Yawn J, Soler ZM (2012). A systematic review of the endoscopic repair of cerebrospinal fluid leaks. Otolaryngol Head Neck Surg.

[205] Schoentgen C, Henaux PL, Godey B, Jegoux F (2013). Management of post-traumatic cerebrospinal fluid (CSF) leak of anterior skull base: 10 years experience. Acta Otolaryngol.

[206] Solyar AY, Fried MP, Goldberg AN, Kennedy DW, Lanza DC (2011). Pedicled nasoseptal flap is not the standard of care for skull base defects. Laryngoscope.

[207] Illing E, Chaaban MR, Riley KO, Woodworth BA (2013). Porcine small intestine submucosal graft for endoscopic skull base reconstruction. Int Forum Allergy Rhinol.

[208] Shi JB, Chen FH, Fu QL, Xu R, Wen WP, Hou WJ, Guo JB, Zhang XM, Xu G (2010). Frontal sinus cerebrospinal fluid leaks: repair in 15 patients using an endoscopic surgical approach. ORL J Otorhinolaryngol Relat Spec.

[209] Cantini Ardila JE, Mendoza MÁ, Ortega VG (2013). Sphenoid sinus and sphenoid bone fractures in patients with craniomaxillofacial trauma. Craniomaxillofac Trauma Reconstr.

[210] Wormald PJ, McDonogh M (1997). 'Bath-plug' technique for the endoscopic management of cerebrospinal fluid leaks. J Laryngol Otol.

[211] Leng LZ, Brown S, Anand VK, Schwartz TH (2008). "Gasket-seal" watertight closure in minimal-access endoscopic cranial base surgery. Neurosurgery.

[212] Hadad G, Bassagasteguy L, Carrau RL, Mataza JC, Kassam A, Snyderman CH, Mintz A (2006). A novel reconstructive technique after endoscopic expanded endonasal approaches: vascular pedicle nasoseptal flap. Laryngoscope.

[213] Caicedo-Granados E, Carrau R, Snyderman CH, Prevedello D, Fernandez-Miranda J, Gardner P, Kassam A (2010). Reverse rotation flap for reconstruction of donor site after vascular pedicled nasoseptal flap in skull base surgery. Laryngoscope.

[214] Wormald PJ, McDonogh M (2003). The bath-plug closure of anterior skull base cerebrospinal fluid leaks. Am J Rhinol.

[215] Raoul G, Gwénaël R, Vanlergerghe B, Benoît V, Ferri J, Joël F (2010). Massive pneumomediastinum and subcutaneous emphysema secondary to isolated zygomaticomaxillary complex fracture. J Craniofac Surg.

[216] Gassner HG, Ponikau JU, Sherris DA, Kern EB (1999). CSF rhinorrhea: 95 consecutive surgical cases with long term follow-up at the Mayo Clinic. Am J Rhinol.

[217] Raveh J, Vuillemin T (1988). Advantages of an additional subcranial approach in the correction of craniofacial deformities. J Craniomaxillofac Surg.

[218] Schaller B (2005). Subcranial approach in the surgical treatment of anterior skull base trauma. Acta Neurochir (Wien).

[219] Schwartz MS, Cohen JI, Meltzer T, Wheatley MJ, McMenomey SO, Horgan MA, Kellogg JX, Delashaw JB (1999). Use of the radial forearm microvascular free-flap graft for cranial base reconstruction. J Neurosurg.

[220] Bolger WE (2005). Endoscopic transpterygoid approach to the lateral sphenoid recess: surgical approach and clinical experience. Otolaryngol Head Neck Surg.

[221] Forer B, Sethi DS (2010). Endoscopic repair of cerebrospinal fluid leaks in the lateral sphenoid sinus recess. J Neurosurg.

[222] Huai RC, Yi CL, Ru LB, Chen GH, Guo HH, Luo L (2008). Traumatic carotid cavernous fistula concomitant with pseudoaneurysm in the sphenoid sinus. Interv Neuroradiol.

[223] Casiano RR, Jassir D (1999). Endoscopic cerebrospinal fluid rhinorrhea repair: is a lumbar drain necessary?. Otolaryngol Head Neck Surg.

[224] Bullock R, Teasdale G (1990). ABC of major trauma. Head injuries--II. BMJ.

[225] (1994). Antimicrobial prophylaxis in neurosurgery and after head injury. Infection in Neurosurgery Working Party of the British Society for Antimicrobial Chemotherapy. Lancet.

[226] Owen P (2002). Prophylaxis for early onset group B streptococcal sepsis is not so effective in practice. BMJ.

[227] Eljamel MS (1993). Antibiotic prophylaxis in unrepaired CSF fistulae. Br J Neurosurg.

[228] Carrion E, Hertzog JH, Medlock MD, Hauser GJ, Dalton HJ (2001). Use of acetazolamide to decrease cerebrospinal fluid production in chronically ventilated patients with ventriculopleural shunts. Arch Dis Child.

[229] Deutsche Gesellschaft für Mund-, Kiefer-und Gesichtschirurgie (DGMKG) (2013). Rekonstruktion von Orbitadefekten. S2e-Leitlinie. AWMF-Register Nr. 007/09. AWMF online.

[230] Joseph JM, Glavas IP (2011). Orbital fractures: a review. Clin Ophthalmol.

[231] Perry M (2008). Acute proptosis in trauma: retrobulbar hemorrhage or orbital compartment syndrome--does it really matter? J Oral Maxillofac Surg. http://dx.doi.org/10.1016/j.joms.2008.04.012.

[232] Burnstine MA (2003). Clinical recommendations for repair of orbital facial fractures. Curr Opin Ophthalmol.

[233] Marinho RO, Freire-Maia B (2013). Management of fractures of the zygomaticomaxillary complex. Oral Maxillofac Surg Clin North Am.

[234] Hatton MP, Rubin PA (2007). Management of orbital compartment syndrome. Arch Ophthalmol.

[235] Lima V, Burt B, Leibovitch I, Prabhakaran V, Goldberg RA, Selva D (2009). Orbital compartment syndrome: the ophthalmic surgical emergency. Surv Ophthalmol.

[236] Davidson TM, Olesen RM, Nahum AM (1975). Medial orbital wall fracture with rectus entrapment. Arch Otolaryngol.

[237] Reim M (Stuttgart). Augenheilkunde.

[238] Erdurman FC, Erdurman FC, Sobaci G, Acikel CH, Ceylan MO, Durukan AH, Hurmeric V (2011). Anatomical and functional outcomes in contusion injuries of posterior segment. Eye (Lond).

[239] Collet S, Grulois V, Bertrand B, Rombaux P (2009). Post-traumatic olfactory dysfunction: a cohort study and update. B-ENT.

[240] Coello AF, Canals AG, Gonzalez JM, Martín JJ (2010). Cranial nerve injury after minor head trauma. J Neurosurg.

[241] Haxel BR, Grant L, Mackay-Sim A (2008). Olfactory dysfunction after head injury. J Head Trauma Rehabil.

[242] Doty RL, Yousem DM, Pham LT, Kreshak AA, Geckle R, Lee WW (1997). Olfactory dysfunction in patients with head trauma. Arch Neurol.

[243] Schriever VA, Studt F, Smitka M, Grosser K, Hummel T (2014). Olfactory function after mild head injury in children. Chem Senses.

[244] London B, Nabet B, Fisher AR, White B, Sammel MD, Doty RL (2008). Predictors of prognosis in patients with olfactory disturbance. Ann Neurol.

[245] Najafi MR, Mehrbod N (2012). Isolated third nerve palsy from mild closed head trauma. Arch Iran Med.

[246] Kim E, Chang H (2013). Isolated oculomotor nerve palsy following minor head trauma : case illustration and literature review. J Korean Neurosurg Soc.

[247] Chen CC, Pai YM, Wang RF, Wang TL, Chong CF (2005). Isolated oculomotor nerve palsy from minor head trauma. Br J Sports Med.

[248] Liguoro D, Viejo-Fuertes D, San Galli F, Dautheribes M, Guérin J (1998). Paralysies oculomotrices et traumatismes crâniens. Neurochirurgie.

[249] Sebag J, Sadun AA (1983). Aberrant regeneration of the third nerve following orbital trauma. Synkinesis of the iris sphincter. Arch Neurol.

[250] Brent BD, May DR (1990). Orbital apex syndrome after penetrating orbital trauma. Ann Ophthalmol.

[251] Jacobson DM, Warner JJ, Choucair AK, Ptacek LJ (1988). Trochlear nerve palsy following minor head trauma. A sign of structural disorder. J Clin Neuroophthalmol.

[252] Goubran GF (1978). Traumatic bilateral abducent nerve palsies. Br J Oral Surg.

[253] Antoniades K, Karakasis D, Taskos N (1993). Abducent nerve palsy following transverse fracture of the middle cranial fossa. J Craniomaxillofac Surg.

[254] Nayil K, Laharwal M, Dhar A, Wani A, Ramzan A, Arif S (2012). Vertex epidural hematoma with bilateral abducent nerve palsy: case report and literature review. Turk Neurosurg.

[255] Warner N, Eggenberger E (2010). Traumatic optic neuropathy: a review of the current literature. Curr Opin Ophthalmol.

[256] Wang DH, Zheng CQ, Qian J, Barr JJ, Anderson AG (2008). Endoscopic optic nerve decompression for the treatment of traumatic optic nerve neuropathy. ORL J Otorhinolaryngol Relat Spec.

[257] Ansari MH (2005). Blindness after facial fractures: a 19-year retrospective study. J Oral Maxillofac Surg.

[258] Magarakis M, Mundinger GS, Kelamis JA, Dorafshar AH, Bojovic B, Rodriguez ED (2012). Ocular injury, visual impairment, and blindness associated with facial fractures: a systematic literature review. Plast Reconstr Surg.

[259] Steinsapir KD, Goldberg RA (1994). Traumatic optic neuropathy. Surv Ophthalmol.

[260] Luxenberger W, Stammberger H, Jebeles JA, Walch C (1998). Endoscopic optic nerve decompression: the Graz experience. Laryngoscope.

[261] von Lanz T, Wachsmuth W (1979). Praktische Anatomie.

[262] Levin LA, Beck RW, Joseph MP, Seiff S, Kraker R (1999). The treatment of traumatic optic neuropathy: the International Optic Nerve Trauma Study. Ophthalmology.

[263] Walsh FB (1966). Pathological-clinical correlations. I. Indirect trauma to the optic nerves and chiasm. II. Certain cerebral involvements associated with defective blood supply. Invest Ophthalmol.

[264] Gellrich NC (1999). Kontroversen und aktueller Stand der Therapie von Sehnervenschäden in der kraniofazialen Traumatologie und Chirurgie. Mund Kiefer Gesichtschir.

[265] Carta A, Ferrigno L, Salvo M, Bianchi-Marzoli S, Boschi A, Carta F (2003). Visual prognosis after indirect traumatic optic neuropathy. J Neurol Neurosurg Psychiatry.

[266] Panja S, Gupta A, Gupta A, Prabhaker S (2010). Determinants of outcome in traumatic optic neuropathy. Otolaryngol Head Neck Surg.

[267] Gellrich NC, Zerfowski M, Eufinger H, Reinert S, Eysel UT (1998). Interdisziplinäre Diagnostik und Therapie der traumatischen Sehnervenschädigung. Mund Kiefer Gesichtschir.

[268] Bach M, Kellner U (2000). Elektrophysiologische Diagnostik in der Ophthalmologie. Ophthalmologe.

[269] Odom JV, Bach M, Barber C, Brigell M, Marmor MF, Tormene AP (2004). Visual evoked potentials standard (2004). Doc Ophthalmol.

[270] Maurer K, Lang N, Eckert J (2005). Visuell evozierte Potentiale (VEP). Praxis der evozierten Potentiale: SEP, AEP, MEP, VEP.

[271] Mahapatra AK (1991). Visual evoked potentials in optic nerve injury--does it merit to be mentioned?. Indian J Ophthalmol.

[272] Tuan LTQ (2006). P30.31 The role of pattern visual evoked potential in the diagnosis of traumatic optic neuropathy. Clin Neurophysiol.

[273] Cerovski B, Sikić J, Juri J, Petrović J (2001). The role of visual evoked potentials in the diagnosis of optic nerve injury as a result of mild head trauma. Coll Antropol.

[274] Villarreal PM, de Vicente JC, Junquera LM (2000). Traumatic optic neuropathy. A case report. Int J Oral Maxillofac Surg.

[275] Kuehnel T (2012). Management of Acute Trauma to the Optic Nerve.

[276] Yu Wai Man P, Griffiths PG (2005). Surgery for traumatic optic neuropathy. Cochrane Database Syst Rev.

[277] Schmidbauer JM, Müller E, Höh H, Robinson E (1998). Transsphenoidale Frühdekompression bei indirekter traumatischer Optikusneuropathie. HNO.

[278] Goodall KL, Brahma A, Bates A, Leatherbarrow B (1999). Lateral canthotomy and inferior cantholysis: an effective method of urgent orbital decompression for sight threatening acute retrobulbar haemorrhage. Injury.

[279] Gerbino G, Ramieri GA, Nasi A (2005). Diagnosis and treatment of retrobulbar haematomas following blunt orbital trauma: a description of eight cases. Int J Oral Maxillofac Surg.

[280] Haubner F, Jägle H, Nunes DP, Schleder S, Cvetkova N, Kühnel T, Gassner HG (2015). Orbital compartment: effects of emergent canthotomy and cantholysis. Eur Arch Otorhinolaryngol.

[281] Sun MT, Chan WO, Selva D (2014). Traumatic orbital compartment syndrome: importance of the lateral canthomy and cantholysis. Emerg Med Australas.

[282] Vassallo S, Hartstein M, Howard D, Stetz J (2002). Traumatic retrobulbar hemorrhage: emergent decompression by lateral canthotomy and cantholysis. J Emerg Med.

[283] Thakar A, Mahapatra AK, Tandon DA (2003). Delayed optic nerve decompression for indirect optic nerve injury. Laryngoscope.

[284] Steinsapir KD, Goldberg RA (2011). Traumatic optic neuropathy: an evolving understanding. Am J Ophthalmol.

[285] Cook MW, Levin LA, Joseph MP, Pinczower EF (1996). Traumatic optic neuropathy. A meta-analysis. Arch Otolaryngol Head Neck Surg.

[286] Yang WG, Chen CT, Tsay PK, de Villa GH, Tsai YJ, Chen YR (2004). Outcome for traumatic optic neuropathy--surgical versus nonsurgical treatment. Ann Plast Surg.

[287] Yu-Wai-Man P, Griffiths PG (2007). Steroids for traumatic optic neuropathy. Cochrane Database Syst Rev.

[288] Anderson RL, Panje WR, Gross CE (1982). Optic nerve blindness following blunt forehead trauma. Ophthalmology.

[289] Young W (1990). NASCIS. National Acute Spinal Cord Injury Study. J Neurotrauma.

[290] Hurlbert RJ (2000). Methylprednisolone for acute spinal cord injury: an inappropriate standard of care. J Neurosurg.

[291] Coleman WP, Benzel D, Cahill DW, Ducker T, Geisler F, Green B, Gropper MR, Goffin J, Madsen PW, Maiman DJ, Ondra SL, Rosner M, Sasso RC, Trost GR, Zeidman S (2000). A critical appraisal of the reporting of the National Acute Spinal Cord Injury Studies (II and III) of methylprednisolone in acute spinal cord injury. J Spinal Disord.

[292] Sayer FT, Kronvall E, Nilsson OG (2006). Methylprednisolone treatment in acute spinal cord injury: the myth challenged through a structured analysis of published literature. Spine J.

[293] Entezari M, Rajavi Z, Sedighi N, Daftarian N, Sanagoo M (2007). High-dose intravenous methylprednisolone in recent traumatic optic neuropathy; a randomized double-masked placebo-controlled clinical trial. Graefes Arch Clin Exp Ophthalmol.

[294] Roberts I, Yates D, Sandercock P, Farrell B, Wasserberg J, Lomas G, Cottingham R, Svoboda P, Brayley N, Mazairac G, Laloë V, Muñoz-Sánchez A, Arango M, Hartzenberg B, Khamis H, Yutthakasemsunt S, Komolafe E, Olldashi F, Yadav Y, Murillo-Cabezas F, Shakur H, Edwards P (2004). Effect of intravenous corticosteroids on death within 14 days in 10008 adults with clinically significant head injury (MRC CRASH trial): randomised placebo-controlled trial. Lancet.

[295] Steinsapir KD, Goldberg RA, Sinha S, Hovda DA (2000). Methylprednisolone exacerbates axonal loss following optic nerve trauma in rats. Restor Neurol Neurosci.

[296] Bracken MB (2012). Steroids for acute spinal cord injury. Cochrane Database Syst Rev.

[297] Batzofin BM, Weiss YG, Ledot SF (2013). Do corticosteroids improve outcome for any critical illness? Curr Opin Anaesthesiol. http://dx.doi.org/10.1097/ACO.0b013e32835e820e.

[298] Kox M, Pickkers P (2013). "Less is more" in critically ill patients: not too intensive. JAMA Intern Med.

[299] Weber AJ, Harman CD (2013). BDNF treatment and extended recovery from optic nerve trauma in the cat. Invest Ophthalmol Vis Sci.

[300] Vega-Meléndez GS, Blagburn JM, Blanco RE (2014). Ciliary neurotrophic factor and fibroblast growth factor increase the speed and number of regenerating axons after optic nerve injury in adult Rana pipiens. J Neurosci Res.

[301] Zhong Y, Shen X, Liu X, Cheng Y (2010). The early effect of nerve growth factor in the management of serious optic nerve contusion. Clin Exp Optom.

[302] Gordon T (2009). The role of neurotrophic factors in nerve regeneration. Neurosurg Focus.

[303] Li KK, Meara JG, Joseph MP (1997). Reversal of blindness after facial fracture repair by prompt optic nerve decompression. J Oral Maxillofac Surg.

[304] Maurer J, Hinni M, Mann W, Pfeiffer N (1999). Optic nerve decompression in trauma and tumor patients. Eur Arch Otorhinolaryngol.

[305] Stoll W, Lübben B, Grenzebach U (2001). Erweiterte Indikationen zur Optikusdekompression: Eine differenzierte Analyse visueller Funktionseinschränkungen--auch bei bewusstlosen Patienten. Laryngorhinootologie.

[306] Jacquesson T, Abouaf L, Berhouma M, Jouanneau E (2014). How I do it: the endoscopic endonasal optic nerve and orbital apex decompression. Acta Neurochir (Wien).

[307] Kountakis SE, Maillard AAJ, Elharazi S, Urso R (1999). Management of traumatic optic neuropathy. Otolaryngol Head Neck Surg.

[308] Xu R, Chen F, Zuo K, Ye X, Yang Q, Shi J, Chen H, Li H (2014). Endoscopic optic nerve decompression for patients with traumatic optic neuropathy: is nerve sheath incision necessary?. ORL J Otorhinolaryngol Relat Spec.

[309] Thaker A, Tandon DA, Mahapatra AK (2009). Surgery for optic nerve injury: should nerve sheath incision supplement osseous decompression?. Skull Base.

[310] Beer-Furlan A, Evins AI, Rigante L, Burrell JC, Anichini G, Stieg PE, Bernardo A (2014). Endoscopic extradural anterior clinoidectomy and optic nerve decompression through a pterional port. J Clin Neurosci.

[311] Moe KS, Bergeron CM, Ellenbogen RG (2010). Transorbital neuroendoscopic surgery. Neurosurgery.

[312] Chen C, Selva D, Floreani S, Wormald PJ (2006). Endoscopic optic nerve decompression for traumatic optic neuropathy: an alternative. Otolaryngol Head Neck Surg.

[313] Dickersin K, Manheimer E, Li T (2006). Surgery for nonarteritic anterior ischemic optic neuropathy. Cochrane Database Syst Rev.

[314] Ohlsson M, Westerlund U, Langmoen IA, Svensson M (2004). Methylprednisolone treatment does not influence axonal regeneration or degeneration following optic nerve injury in the adult rat. J Neuroophthalmol.

[315] Septa D, Newaskar VP, Agrawal D, Tibra S (2014). Etiology, incidence and patterns of mid-face fractures and associated ocular injuries. J Maxillofac Oral Surg.

[316] Hwang K, Kim DH (2011). Analysis of zygomatic fractures. J Craniofac Surg.

[317] Azenha MR, Yamaji MA, Avelar RL, de Freitas QE, Laureano Filho JR, de Oliveira Neto PJ (2011). Retropharyngeal and cervicofacial subcutaneous emphysema after maxillofacial trauma. Oral Maxillofac Surg.

[318] Lee EI, Mohan K, Koshy JC, Hollier LH (2010). Optimizing the surgical management of zygomaticomaxillary complex fractures. Semin Plast Surg.

[319] Evans GR, Daniels M, Hewell L (2011). An evidence-based approach to zygomatic fractures. Plast Reconstr Surg.

[320] Bansagi ZC, Meyer DR (2000). Internal orbital fractures in the pediatric age group: characterization and management. Ophthalmology.

[321] Rana M, Warraich R, Tahir S, Iqbal A, von See C, Eckardt AM, Gellrich NC (2012). Surgical treatment of zygomatic bone fracture using two points fixation versus three point fixation--a randomised prospective clinical trial. Trials.

[322] Ridgway EB, Chen C, Colakoglu S, Gautam S, Lee BT (2009). The incidence of lower eyelid malposition after facial fracture repair: a retrospective study and meta-analysis comparing subtarsal, subciliary, and transconjunctival incisions. Plast Reconstr Surg.

[323] Ellis E, Zide MF, Ellis E, Zide MF (2005). Coronal approach. Surgical approaches to the facial skeleton.

[324] Jaquiéry C, Leiggener C, Cornelius CP, Kunz Ch (2013). Aktuelle Behandlungsstrategien von knöchernen Verletzungen der Orbitae. OP-Journal.

[325] Hanken H, Lohse C, Assaf AT, Heiland M (2013). Intraoperative Bildgebung in der Mund-, Kiefer- und Gesichtschirurgie. OP-Journal.

[326] Xie L, Shao Y, Hu Y, Li H, Gao L, Hu H (2009). Modification of surgical technique in isolated zygomatic arch fracture repair: seven case studies. Int J Oral Maxillofac Surg.

[327] Curtis W, Horswell BB (2013). Panfacial fractures: an approach to management. Oral Maxillofac Surg Clin North Am.

[328] Pau M, Reinbacher KE, Feichtinger M, Navysany K, Kärcher H (2014). The mandibular symphysis as a starting point for the occlusal-level reconstruction of panfacial fractures with bicondylar fractures and interruption of the maxillary and mandibular arches: report of two cases. J Craniomaxillofac Surg.

[329] Babl FE, Lyttle MD, Bressan S, Borland M, Phillips N, Kochar A, Dalziel SR, Dalton S, Cheek JA, Furyk J, Gilhotra Y, Neutze J, Ward B, Donath S, Jachno K, Crowe L, Williams A, Oakley E, PREDICT research network (2014). A prospective observational study to assess the diagnostic accuracy of clinical decision rules for children presenting to emergency departments after head injuries (protocol): the Australasian Paediatric Head Injury Rules Study (APHIRST). BMC Pediatr.

[330] Massarelli O, Gobbi R, Soma D, Raho MT, Tullio A (2011). An aesthetically possible alternative approach for craniomaxillofacial trauma: the "pretrichial incision". Craniomaxillofac Trauma Reconstr.

[331] Eljamel MS (1998). Antibiotic prophylaxis in the management of CSF fistula. Surg Neurol.

[332] Carrim ZI, Anderson IW, Kyle PM (2007). Traumatic orbital compartment syndrome: importance of prompt recognition and management. Eur J Emerg Med.

[333] Edwards P, Arango M, Balica L, Cottingham R, El-Sayed H, Farrell B, Fernandes J, Gogichaisvili T, Golden N, Hartzenberg B, Husain M, Ulloa MI, Jerbi Z, Khamis H, Komolafe E, Laloë V, Lomas G, Ludwig S, Mazairac G, Muñoz Sanchéz Mde L, Nasi L, Olldashi F, Plunkett P, Roberts I, Sandercock P, Shakur H, Soler C, Stocker R, Svoboda P, Trenkler S, Venkataramana NK, Wasserberg J, Yates D, Yutthakasemsunt S (2005). Final results of MRC CRASH, a randomised placebo-controlled trial of intravenous corticosteroid in adults with head injury-outcomes at 6 months. Lancet.

[334] Koento T (2012). Current advances in sinus preservation for the management of frontal sinus fractures. Curr Opin Otolaryngol Head Neck Surg.

[335] Di Rocco F, Couloigner V, Dastoli P, Sainte-Rose C, Zerah M, Roger G (2010). Treatment of anterior skull base defects by a transnasal endoscopic approach in children. J Neurosurg Pediatr.

